# Discovery of (*E*)-1,3-Diphenyl-2-Propen-1-One Derivatives as Potent and Orally Active NLRP3 Inflammasome Inhibitors for Colitis

**DOI:** 10.3390/molecules30163340

**Published:** 2025-08-11

**Authors:** Liuzeng Chen, Xiaoyu Zheng, Jiahui Li, Bin Zhou, Min Tao, Yuetian Yang, Yi Wang, Hao Zhan, Guoping Zhang, Jingbo Shi, Xingxing Zhang, Banfeng Ruan

**Affiliations:** 1 School of Biology, Food and Environment, Hefei University, Hefei 230601, China; clz@hfuu.edu.cn (L.C.); 13133517200@163.com (X.Z.); 18130626743@163.com (J.L.); zhoubin00163@163.com (B.Z.); 13696519598@163.com (M.T.); 18326671092@163.com (Y.Y.); wangyi__2023@163.com (Y.W.); 15385372606@163.com (H.Z.); 2 School of Pharmacy, Anhui University of Chinese Medicine, Hefei 230601, China; sjbo616@126.com; 3 College of Chemistry and Materials Science, Huaibei Normal University, Huaibei 235000, China; hbzgp-1@163.com

**Keywords:** inflammation, inhibitor, chalcone derivatives, IBD

## Abstract

The pyrin domain-containing protein 3 (NLRP3) inflammasome may be a potential target for the treatment of inflammatory bowel disease (IBD), and inhibiting the activation of the NLRP3 inflammasome is of great significance for the treatment of IBD. In this study, 27 novel chalcone derivatives were designed and synthesized. Enzyme-linked immunosorbent assay (ELISA) analysis revealed that most of the compounds inhibited IL-1β secretion, with **F14** exhibiting the most significant activity, showing IC_50_ values of 0.74 μM (mouse bone marrow-derived macrophage, BMDM) and 0.88 μM (Tohoku Hospital Pediatrics-1, THP-1), respectively. Flow cytometry and immunofluorescence analysis revealed that **F14** had no effect on mitochondrial reactive oxygen species (ROS) production or mitochondrial damage, nor did it affect the expression of key protein components of the NLRP3 inflammasome. Western blot and computational docking studies suggested that **F14** may exert anti-inflammatory activity by targeting NLRP3 to block the oligomerization and speck formation of ASC protein. In vivo studies demonstrated that **F14** exhibited significant therapeutic effects on dextran sulfate sodium (DSS)-induced acute colitis in mice. Overall, this work provides candidate compounds for the development of NLRP3 inflammasome inhibitors and the treatment of inflammatory diseases caused by NLRP3 inflammasome activation.

## 1. Introduction

Inflammatory bowel disease (IBD) is a complex disease characterized by chronic, relapsing gastrointestinal inflammation [1]. With changes in human dietary patterns, increased environmental risk factors, and frequent population movement, the epidemiological characteristics of IBD have undergone significant changes [1,2]. Its incidence has not only continued to rise in Western countries, but in recent years, it has also shown a rapid growth trend in Asia [3]. Clinically, IBD mainly includes Ulcerative Colitis (UC) and Crohn’s Disease (CD) [4]. The pathogenesis of IBD is complex, involving various factors such as environment, immunity, genetics, microbiota, and psychology [5].

At present, the treatment of IBD in clinical practice mainly focuses on controlling inflammation and maintaining remission [6]. The existing IBD treatment drugs mainly include aminosalicylates (such as sulfasalazine), corticosteroids (such as prednisone), and immunosuppressants (such as azathioprine) [7,8]. Although these drugs can alleviate some symptoms, they still have limitations in efficacy, high recurrence rates, and significant long-term side effects [9,10]. In recent years, emerging biological inhibitors have gradually become the mainstream treatment for IBD [11]. Biologics, including tumor necrosis factor (TNF) inhibitors (e.g., infliximab, adalimumab) and interleukin inhibitors, have significantly improved the treatment landscape for IBD [12]. However, their long-term use still faces challenges such as drug resistance, secondary infections, and potential cancer risks [12,13]. It is of great scientific significance and value to search for novel therapeutic targets and develop small-molecule drugs with definite efficacy, as existing clinical drugs cannot meet the urgent needs of IBD patients. The NLRP3 inflammasome in the NOD-like receptor family is a potential therapeutic target [14].

In the pathological progression of IBD, the excessive activation or imbalance of the NLRP3 inflammasome is a key factor contributing to the persistent inflammatory state [15]. The NLRP3 inflammasome, as a crucial component of the innate immune system, is significantly activated during this process [16]. Under inflammatory conditions, NLRP3 interacts with the CARD domain of ASC through its pyrin domain, subsequently recruiting pro-caspase-1 to form a functional inflammasome complex [17]. Activated caspase-1 not only mediates the maturation and secretion of pro-IL-1β and pro-IL-18 but also induces pyroptosis by cleaving Gasdermin D (GSDMD) protein [17]. IL-1β, as a potent pro-inflammatory factor, recruits neutrophils and monocytes, significantly enhancing the inflammatory response [18], while IL-18 exacerbates the imbalance in intestinal immune responses by promoting the differentiation of Th1 and Th17 cells [19]. Clinical studies show that the secretion levels of IL-1β in the colon tissue and macrophages of IBD patients are significantly positively correlated with disease severity [20,21]. Preclinical studies have also confirmed that IL-18 and pyroptosis play significant roles in the occurrence and development of colitis [22]. The excessive activation of the NLRP3 inflammasome triggers pyroptosis, leading to the death of intestinal epithelial cells, disrupting the intestinal barrier function, and increasing intestinal permeabilit, making it easier for bacteria and their metabolic products to enter the submucosa, further activating the immune system and forming a vicious cycle [23]. Animal experiments have shown that NLRP3^−/−^, ASC^−/−^, and caspase-1^−/−^ mice exhibit significant protective effects in the DSS-induced colitis model [24]. These findings confirm the significant role of the NLRP3 inflammasome in the pathological process of IBD.

**Figure 1:**
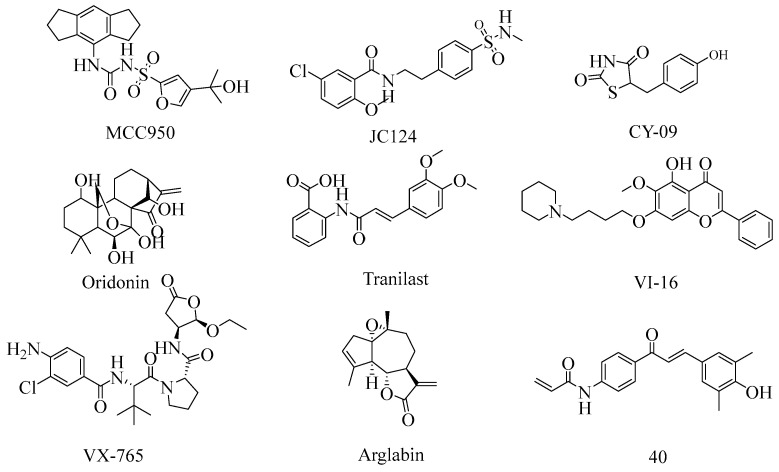
Representative NLRP3 inflammasome inhibitor.

Even though dozens of small-molecule compounds have entered clinical research stages worldwide, no drugs have yet been successfully approved for marketing due to factors such as inherent toxicity and pharmacokinetic deficiencies (Figure 1) [25]. MCC950, a highly selective NLRP3 inhibitor, demonstrated significant anti-inflammatory effects in preclinical studies but was discontinued in Phase II clinical trials due to inducing unacceptable hepatic toxicity [26,27]. The sulfonamide derivative JC124 enhanced its targeted binding affinity for NLRP3 through structural optimization [28]. However, its druggability suffers from significant defects, such as low bioavailability and unstable in vivo metabolism, preventing it from achieving effective therapeutic concentrations and limiting further translational research [29]. Although CY-09 and andrographolide have shown clear inhibitory effects, they exhibit strong cytotoxicity and have a narrow therapeutic window [30]. Although tunicamycin has been confirmed to have inhibitory activity, its clinical use requires high-dose administration, which results in significant off-target effects and an increased incidence of side effects. Oridonin, tranilast, VI-16, VX-765, and arglabin as NLRP3 inflammasome inhibitors have also failed to become viable drugs due to their inherent limitations [31,32,33,34,35]. For example, oridonin has poor water solubility and high doses can cause liver toxicity; the high dosage of tranilast may affect other immune responses; kidney problems occur at high doses of arginine; VI-16 and VX-765 are currently in preclinical research. Based on this, the discovery of novel structural scaffolds is of great importance for the development of NLRP3 inflammasome inhibitors.

Natural products, due to their unique chemical structures and diverse biological activities, exhibit great potential in drug development. They not only possess advantages such as structural diversity and low toxicity, but also provide a rich library of lead compounds for modern drug discovery due to their wide availability and long history of traditional use [36]. Chalcones, a class of natural products widely found in plants, have attracted considerable attention due to their significant anti-inflammatory, antioxidant, and immunomodulatory activities [37]. Studies have shown that structural optimization based on the chalcone scaffold exhibits strong inhibitory effects on NLRP3 inflammasome activation (compound **40**) [38]; however, the mechanism of action and the specific target of the compound remain unclear. Our research group previously established a high-content screening model at the cellular level [39], and through screening a natural product library, we found that chalcones exhibit a certain inhibitory effect on NLRP3 inflammasome activation. Based on the excellent pharmacological properties of the chalcone skeleton, it exhibits significant skeletal advantages in drug discovery. This project, therefore, uses the chalcone scaffold as the core structure, conducting a series of structural optimizations and modifications. Through repeated synthesis and activity evaluations, a series of chalcone derivatives was designed, and compound **F14** was found to exhibit good biological activity.

## 2. Results and Discussion

### 2.1. Subsection

**Figure 2:**
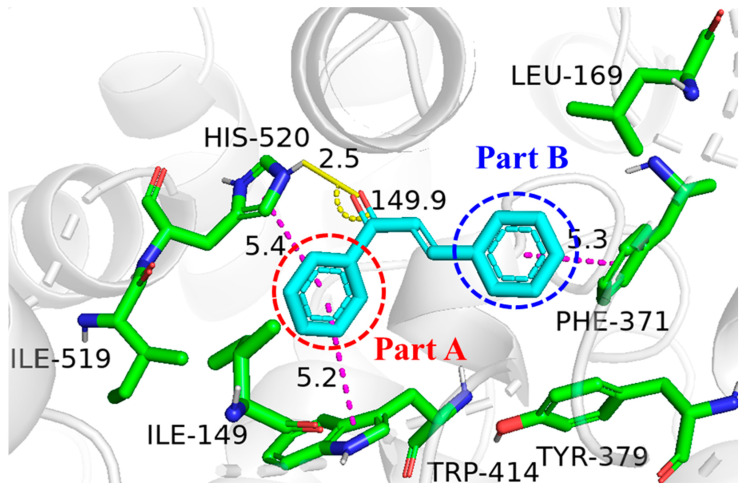
Binding mode of chalcone with the active site of NLRP3 (PDB: 6NPY). NLRP3 is shown in gray ribbons with selected residues colored green. Hydrogen bonds are drawn as yellow dashed lines, and pi-pi stacking is drawn as magenta dashed lines. Chalcone is shown with a blue stick. The illustration was generated using PyMOL.

It has been reported that chalcone could serve as a potential scaffold for the aggregation of NLRP3 inhibitors [40,41]. And its derivative licochalcone B has been reported to be able to specifically inhibit the NLRP3 inflammasome by disrupting NEK7-NLRP3 interaction [42]. Therefore, in this study, the eutectic complex NEK7-NLRP3 (PDB: 6NPY) was selected as the receptor, and the previously screened chalcone was chosen as the ligand for molecular docking. The binding mode of chalcone with the active site of NLRP3 was analyzed, as shown in Figure 2. Not only does chalcone form one hydrogen bond with His520, but it also forms pi-pi stacking interactions with His520, Trp414, and Phe379, respectively. The activity of pi-pi stacking interaction is closely related to the electric abundance of the electron cloud and the central position of the electron cloud. Therefore, the substituents on Part A and Part B of the benzene ring have important effects on the activity of the compounds. Importantly, we noticed that there is a large space around the cavity where the benzene rings of part A and part B are located, which could be occupied by substituents to enhance the pi-pi stacking interaction and improve the activity of the compounds.

### 2.2. Synthesis of Compounds

**Scheme 1:**
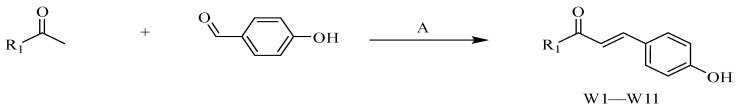
Reagents and conditions: (A) NaOH, Ethanol, 12 h, 25 °C.

**Scheme 2:**
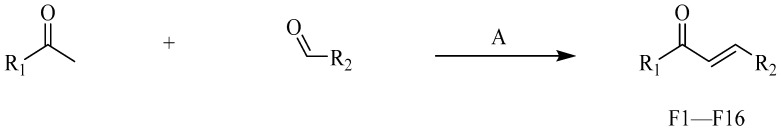
Reagents and conditions: (A) NaOH, Ethanol, 12 h, 25 °C.

Under alkaline conditions, addition reactions occur between different substituted methyl ketones and 4-hydroxybenzaldehyde, followed by further dehydration to obtain unsaturated olefins (Scheme 1 and Scheme 2), ultimately resulting in different substituted 4-hydroxybenzophenone compounds **W1**–**W11** (Table 1) and **F1**–**F16** (Table 2).

### 2.3. SAR Study

The MTT assay revealed that the majority of compounds exhibited good safety profiles (IC_50_ > 50 μM) (Table 3 and Table 4). The in vitro model was used to assess the secretion of IL-1β in LPS/nigericin-treated BMDM cells by W series compounds. As shown in Table 3, when the concentration of **W** series compounds was 20 μM, the compounds exhibited significant anti-inflammatory activity. Structure-activity relationship (SAR) analysis revealed that introducing a methoxy group at the para position or a methyl group at the ortho-para position at site A significantly enhanced activity, as seen in **W2** and **W8**. A comparison of compounds **W1** and **W11** indicated that the introduction of thiophene and naphthalene rings at site A showed better activity than a benzene ring, indirectly suggesting that increasing the spatial volume at site B may enhance the anti-inflammatory activity of the **W** series compounds.

The results of testing the **F** series compounds indicate that this series of compounds exhibits significantly greater anti-inflammatory activity. Compared to the **W**-series compounds, it was found that the introduction of hydroxyl groups at the ortho or meta positions in the B-ring led to a substantial increase in compound activity. Moreover, ortho-hydroxyl groups exhibited higher activity than meta-hydroxyl groups, as demonstrated by compounds **F2**, **F3**, **F7**, and **F15**. This suggests that modifications to the hydroxyl group in the B-ring have a greater impact on the biological activity of the compounds, possibly due to the improved solubility of ortho-meta hydroxyl compounds. Compound **F14** stands out the most; it introduces ortho-hydroxyl groups in both the A and B rings and converts the unsaturated double bond in the C-ring to a single bond. At a concentration of 20 μM, **F14** inhibits IL-1β by 95.44%. In summary, we have selected the highly active and low-toxicity compound **F14** as the target compound for further activity and mechanism studies.

### 2.4. The Anti-Inflammatory Activity of ***F14***

**Figure 3:**
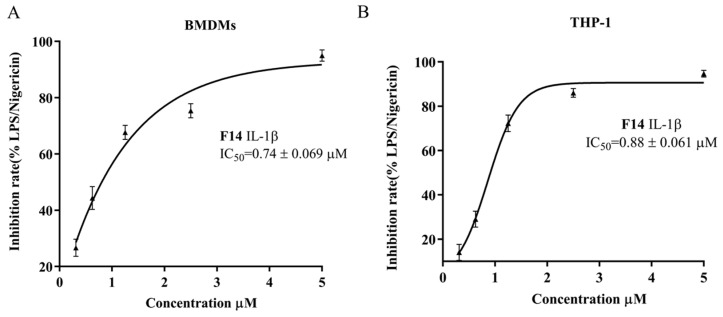
**F14** inhibits the secretion of IL-1β. (**A**) Detection of **F14** inhibition of IL-1 β secretion in BMDM cells; (**B**) Detection of **F14** inhibition of IL-1 β secretion in THP-1 cells. IC_50_ calculation and dose-response curve fitting were performed using GraphPad Prism (8.0) software. All data were obtained from three independent experiments.

Through SAR analysis, we identified compound **F14** as the target compound for further investigation. To further validate its activity, the half-inhibitory concentration (IC_50_) of compound **F14** for IL-1β release was determined in the BMDM model. The results showed that compound **F14** had an IC_50_ value of 0.74 μM in BMDMs (Figure 3A). To more comprehensively assess the potential of compound **F14**, we further evaluated its inhibition of IL-1β in THP-1 cells. Experimental results demonstrated that compound **F14** also exhibited significant IL-1β inhibition in THP-1 cells, with an IC_50_ value of 0.88 μM (Figure 3B). In conclusion, we believe that **F14** strongly inhibits the activation of the NLRP3 inflammasome, warranting further investigation into its anti-inflammatory mechanisms.

### 2.5. Compound ***F14*** Inhibits Cell Pyroptosis Caused by NLRP3 Inflammasome Activation

**Figure 4:**
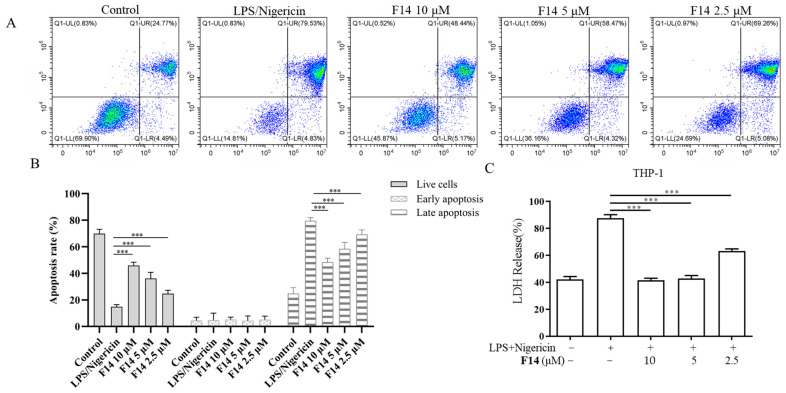
**F14** inhibits NLRP3 inflammasome activation, leading to cell pyroptosis. (**A**,**B**) Apoptosis assay kit is used to detect the level of cell apoptosis and analyze the effect of **F14** on cell apoptosis; (**C**) LDH assay kit is used to detect the release level of lactate dehydrogenase (LDH). Data are presented as the mean ± SEM (*n* = 3), *** *p* < 0.001 vs. the Nigericin/LPS.

Pyroptosis is a form of programmed cell death, typically triggered by the activation of inflammasomes within the cell. It is characterized by the rupture of the cell membrane, releasing cellular contents (such as Lactate Dehydrogenase, LDH), which enhances the inflammatory response. Unlike apoptosis, pyroptosis is initiated through the activation of inflammasomes, involving distinct molecular mechanisms, and is often accompanied by cell swelling and rupture. In contrast, apoptosis is triggered by the activation of Caspase-3, leading to systemic degradation within the cell while maintaining the integrity of the cell membrane. The cell undergoes controlled self-degradation without provoking an immune response [43]. Inhibiting pyroptosis is crucial for alleviating inflammation and promoting tissue repair. To investigate the effect of compound **F14** on pyroptosis, we employed flow cytometry and lactate dehydrogenase (LDH) release assays for systematic analysis. Flow cytometry was used to assess the impact of **F14** on pyroptosis under inflammatory conditions. The results showed that, in the BMDM cells under inflammatory conditions, the LPS/Nigericin treatment group exhibited a significantly lower percentage of viable cells and a higher percentage of late apoptotic cells compared to the control group. However, upon treatment with **F14**, both the viable cell percentage and late apoptotic cell percentage were significantly improved, with more pronounced effects observed at higher concentrations (Figure 4A,B). The results of the LDH release assay indicated that, under NLRP3 inflammasome activation conditions, the LPS/Nigericin treatment group exhibited a significant increase in LDH release. After treatment with **F14**, the LDH release decreased in a concentration-dependent manner, demonstrating that **F14** protects cell membrane integrity (Figure 4C). These experimental results demonstrate that **F14** effectively inhibits pyroptosis induced by abnormal activation of the NLRP3 inflammasome.

### 2.6. Compound ***F14*** Inhibits NLRP3 Inflammasome Activation Induced by Multiple Agonists, Without Affecting AIM2 and NLRC4 Inflammasome Activation

**Figure 5:**
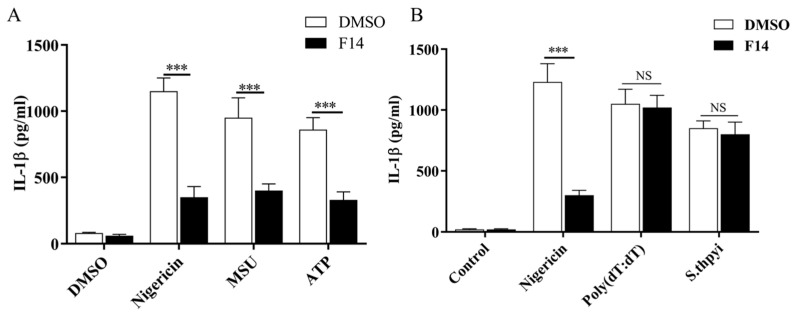
**F14** specifically inhibits NLRP3 inflammasome activation. The LPS concentration was 200 ng/mL to pretreat BMDM cells for 3 h, then **F14** (10 μM) was added to treat the cells for 30 min, and then each inducer—Nigericin (10 μM, 0.5 h), ATP (2.5 mM, 0.5 h), MSU (150 μg/mL, 4 h), S.typhi (4 h), and poly-(dA:dT) (0.5 μg/mL, 4 h)—were added for stimulation. (**A**) **F14** has a broad-spectrum inhibitory effect on NLRP3 inflammasome activation. (**B**) **F14** has a specific inhibitory effect on NLRP3 inflammasome activation. Data are presented as the mean ± SEM (*n* = 3). *** *p* < 0.001 vs. the model group. NS, not significant.

The NLRP3 inflammasome can be activated by various stimuli, such as microbial toxins and urate crystals. This process plays a central role in the pathogenesis of many inflammatory diseases. To assess whether **F14** exerts a broad-spectrum inhibitory effect on NLRP3 inflammasome activation, we used three different activators—ATP, MSU, and Nigericin to stimulate BMDMs. Elisa analysis revealed that in the **F14**-treated group, the protein expression levels of mature IL-1β were significantly reduced (Figure 5A). These findings suggest that **F14** is a broad-spectrum NLRP3 inhibitor. Among the inflammasome family, NLRC4 and AIM2 are also protein complexes involved in the innate immune response, playing key roles in inflammatory regulation during infection and tissue damage. Poly (dA:dT) was used to induce AIM2 activation, while S.typhi was employed to stimulate NLRC4 activation. The results showed that **F14** significantly inhibited the activation of the NLRP3 inflammasome, but had no effect on the activation of NLRC4 or AIM2 inflammasomes under their respective inducers (Figure 5B). In conclusion, **F14** is a specific and broad-spectrum NLRP3 inflammasome inhibitor.

### 2.7. The Effect of ***F14*** on the Expression of Major Protein Components of NLRP3 Inflammasome

**Figure 6:**
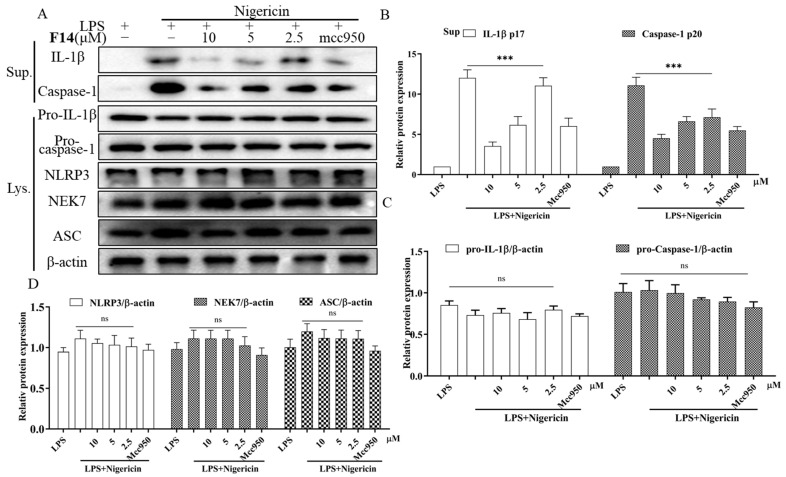
**F14** inhibits NLRP3 inflammasome activation. Western blot analysis of IL-1β, caspase-1, pro-IL-1β, pro-caspase-1, NEK7, NLRP3, and ASC in BMDMs cell. (**A**) Western blot results of the expression levels of proteins. (**B**–**D**) Statistical analysis of the expression levels. The data shown are the mean ± SD of three independent experiments. Data are presented as the mean ± SEM (*n* = 3). ns, not significant, *** *p* < 0.001 vs. the Nigericin/LPS.

To further investigate the potential molecular mechanisms of compound **F14**, an in vitro inflammation model was constructed using BMDM cells. Western blot analysis showed that after treatment with LPS and Nigericin, the protein levels of IL-1β and caspase-1 in the cell supernatant significantly increased. However, in the **F14**-treated group, a concentration-dependent inhibitory effect was observed, indicating that **F14** effectively blocked Nigericin-induced activation of the NLRP3 inflammasome (Figure 6). Further research found that **F14** had no significant effect on the protein expression levels of NLRP3 inflammasome-related components (including pro-IL-1β, pro-caspase-1, ASC, NEK7, and NLRP3), indicating that its inhibitory effect was not achieved by affecting the expression of major proteins.

### 2.8. ***F14*** Does Not Affect Mitochondrial Damage and Reactive Oxygen Species Production During the NLPR3 Inflammasome Activation Process

**Figure 7:**
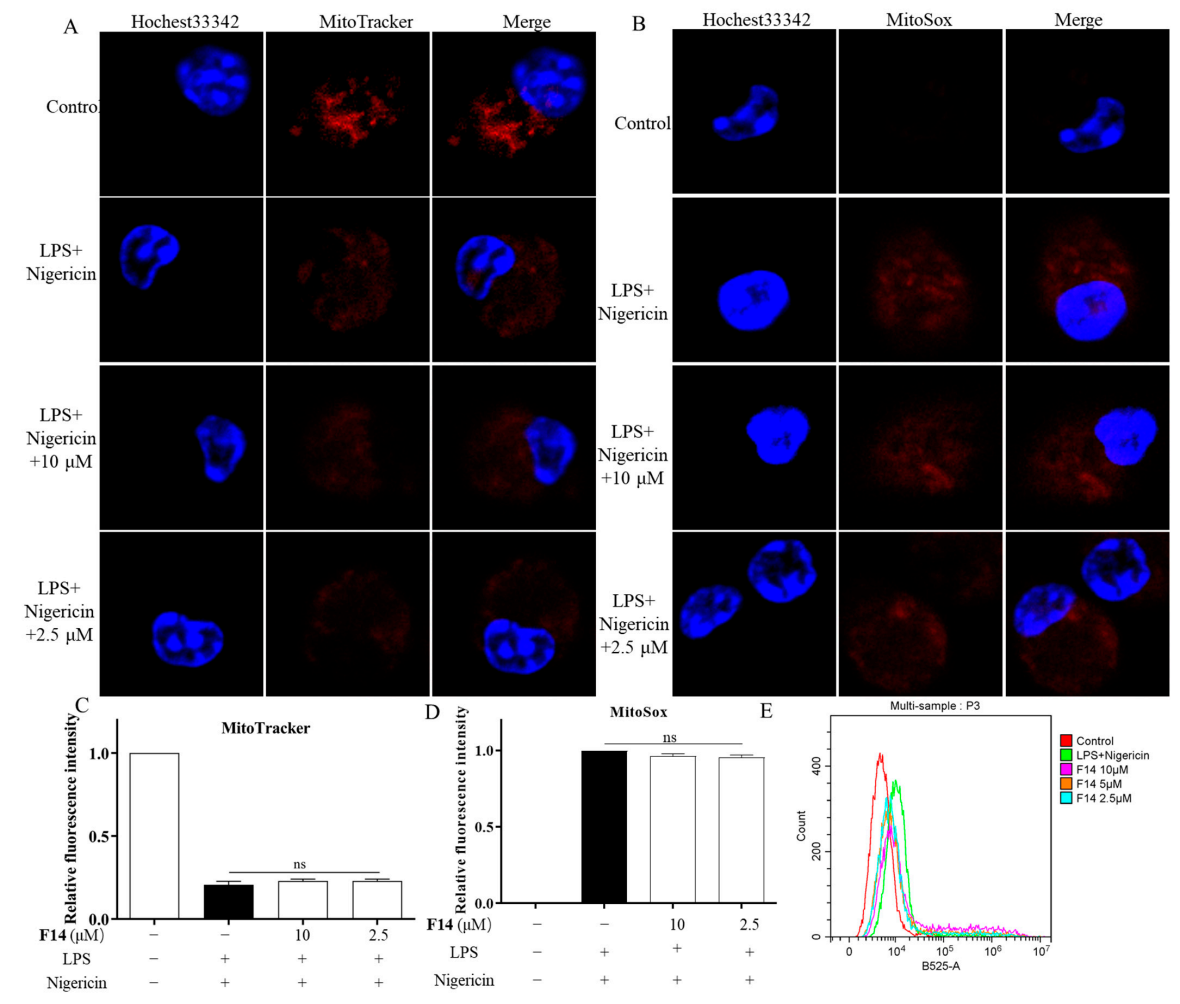
**F14** does not affect ROS and mitochondrial damage. (**A**) MitoTracker staining; (**B**) MitoSOX staining; (**C**,**D**) Statistical analysis and plotting of staining results using ImagJ and GraphPad Prism software; (**E**) ROS detection kit for testing. Data are presented as the mean ± SEM (*n* = 3). ns, not significant.

Mitochondrial dysfunction and excessive production of reactive oxygen species (ROS) are key upstream signaling events in the activation of the NLRP3 inflammasome. To investigate whether compound **F14** affects mitochondrial damage and ROS production, we performed staining experiments using MitoTracker and MitoSOX, followed by imaging and analysis using a confocal microscope. Experimental results showed that treatment with LPS and Nigericin led to a significant decrease in mitochondrial membrane potential and a substantial increase in mitochondrial superoxide levels. However, in the **F14**-treated group, no significant improvement in mitochondrial membrane potential or fluorescence intensity of mitochondrial superoxide anions was observed (Figure 7A–D). This result suggests that compound **F14** does not affect mitochondrial damage during NLRP3 inflammasome activation. To further verify the effect of **F14** on ROS levels, we used flow cytometry to measure intracellular ROS levels in BMDM cells stimulated with Nigericin. The results showed that total ROS levels significantly increased in the LPS and Nigericin-treated groups, while no significant change in total ROS levels was observed in the **F14**-treated group (Figure 7E). This further confirms that **F14** does not affect the production of ROS under inflammatory conditions. In summary, compound **F14** does not intervene in mitochondrial damage or affect ROS generation during NLRP3 inflammasome activation, indicating that compound **F14** does not affect the upstream signaling of NLRP3 inflammasome activation.

### 2.9. ***F14*** Inhibits ASC Polymerization During the NLRP3 Inflammasome Activation Process

**Figure 8:**
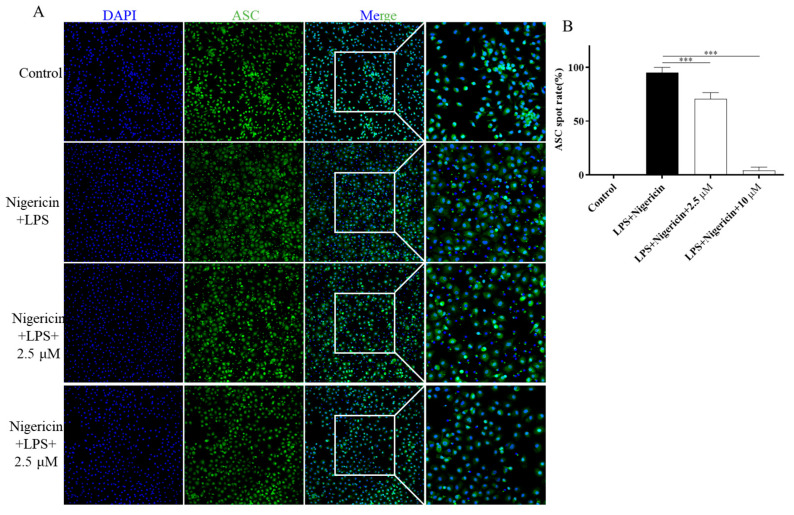
**F14** inhibits ASC polymerization formation. (**A**) Immunofluorescence detection of ASC spots; (**B**) ASC immunofluorescence spot count. Data are presented as the mean ± SEM (*n* = 3). *** *p* < 0.001 vs. the Nigericin/LPS.

The formation of ASC oligomers is a key hallmark of inflammasome activation. ASC forms interactions with other ASC molecules through its pyrin domain (PYD), which leads to the aggregation of these molecules, resulting in the formation of a speck-like structure (ASC specks). This process involves not only the aggregation of proteins but also the assembly of a highly ordered structure in the cytoplasm. Inhibition of ASC oligomerization can effectively block NLRP3 inflammasome activation. Based on previous experimental results, we found that **F14** can inhibit NLRP3 inflammasome activation without affecting the expression of related proteins or upstream signaling. Therefore, we hypothesize that **F14** may inhibit caspase-1 activation by interfering with ASC oligomerization or ASC speck formation. To validate this hypothesis, we conducted immunofluorescence experiments in BMDM cells under inflammatory conditions. Confocal microscopy observations revealed a large number of ASC green fluorescence speckles in the LPS and Nigericin-treated groups, while the **F14**-treated group showed a significant reduction in ASC speckles, with this inhibitory effect being concentration-dependent (Figure 8). In conclusion, **F14** blocks NLRP3 inflammasome activation by inhibiting the formation of ASC oligomers.

### 2.10. ***F14*** Exerts Anti-Inflammatory Activity by Affecting NLRP3 Inflammasome Assembly

**Figure 9:**
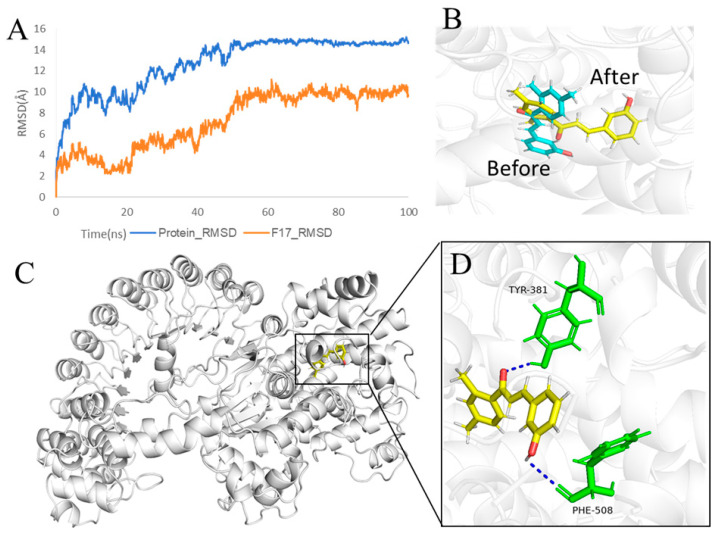
Docking analysis of compound **F14** targeting the NLRP3 protein. (**A**) The RMSDs of all the atoms of the **F14** complex with respect to its initial structure as a function of time. (**B**–**D**) Simulate and predict the binding mode between compound **F14** and NLRP3.

Summarizing the above experimental results, it was found that compound **F14** does not affect the upstream signal of NLRP3 inflammasome activation, but affects the formation of ASC spots downstream of NLRP3 inflammasome activation; and it has no effect on the expression of major proteins. We hypothesize that compound **F14** may target the NLRP3 protein to exert its anti-inflammatory activity. To further analyze the binding mode of **F14** with the NLRP3 protein, molecular docking was used to predict its binding with the NLRP3 complex, followed by molecular dynamics for conformational optimization. Based on the simulation results, the binding free energy between **F14** and NLRP3 was calculated to be −8.5 ± 1.5 kcal/mol, indicating a good binding interaction between the compound and NLRP3. Using the first frame structure in the simulated trajectory as a reference, calculate the corresponding RMSD value (Figure 9A). In the early stages of the simulation, the protein conformation underwent significant changes, likely due to the presence of flexible chains in the large molecular structure of NLRP3. On the other hand, the RMSD value of the small molecule also showed significant changes in the early stages of the simulation, suggesting that its conformation was being optimized. In the later stages of the simulation, the RMSD values of the components in the system stabilized, indicating that equilibrium had been reached. Additionally, we analyzed the RMSF changes of **F14** and NLRP3 protein (Supplementary Figure S1). The results indicated that the phenyl ring connected to the hydroxyl group exhibited significant fluctuations, likely due to the interactions between the hydroxyl group and protein residues, which cause pulling forces. Furthermore, the two methyl groups attached to the other phenyl ring also exhibited considerable changes, suggesting they may have an important impact on activity. Finally, the RMSF changes in the protein amino acids remained relatively stable, except for the PYD domain, which exhibited significant fluctuations due to its higher flexibility. Based on the above analysis, the overall system reached a relatively stable state in the later stages of the simulation, and the final frame structure was extracted from the simulation trajectory for further analysis. By comparing the conformational changes of the small molecule before and after the simulation, it was found that its structure underwent a flip (Figure 9B), with **F14** inserting directly into the active pocket. The carbonyl and hydroxyl groups of its structure form hydrogen bonds with TYR381 and PHE-508, respectively, maintaining conformational stability (Figure 9C,D). Overall, compound **F14** may exert anti-inflammatory activity by targeting the NLRP3 protein, and the amino acids TYR381 and PHE-508 in NLRP3 may be key residues influencing the molecule’s activity.

### 2.11. In Vivo Biological Activity of ***F14***

**Figure 10:**
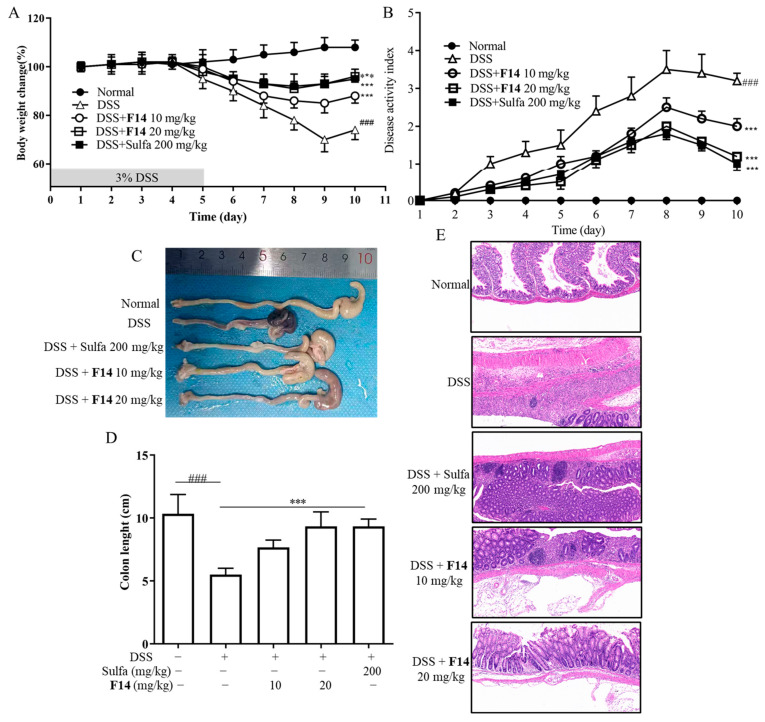
The therapeutic effect of **F14** on DSS-induced acute colitis in mice. (**A**) Changes in body weight of mice; (**B**) Mouse DAI score; (**C**) Macroscopic changes of the colon in mice induced by DSS. (**D**) Mouse colon length. (**E**) HE staining analysis of colon tissue. Data are presented as the mean ± SEM (*n* = 5). ^###^ *p* < 0.001 compared with the Normal group; *** *p* < 0.001 vs. DSS.

To further validate the in vivo biological activity of compound **F14**, we used the DSS-induced mouse colitis model to assess its anti-inflammatory effects. The experimental design was as follows: mice were divided into the normal group, model group (DSS), positive control group (DSS + sulfasalazine), low-dose group (DSS + 10 mg/kg), and high-dose group (DSS + 20 mg/kg). Mice in the normal group were given regular water with no treatment; the positive control group received sulfasalazine as the positive drug at a daily dose of 200 mg/kg; the low-dose and high-dose groups were administered 10 mg/kg and 20 mg/kg of **F14**, respectively, each day. During the experiment, acute colitis in mice was induced by drinking water containing 3% DSS for 10 days. The mice’s body weight changes, fecal blood presence, and disease activity index (DAI) were recorded daily. The results indicate that compared to the model group, **F14** significantly alleviates the weight loss in mice and inhibits the increase in the disease activity index (Figure 10A,B), demonstrating its marked anti-inflammatory effects. Dissecting mice revealed that compound **F14** effectively alleviated colonic adhesion in mice (Figure 10C,D). Goblet cells are a type of colon epithelial cell, and their primary function is to secrete mucus, which helps maintain the stability of the intestinal environment. During the pathological process of colitis, the number of goblet cells decreased, and their mucus-secreting function was impaired. In histological examinations, the number and secretion function of goblet cells are important indicators of colitis. Histological analysis of mouse colon tissue indicates that compound **F14** inhibits the reduction of goblet cells, suggesting its protective effect on the colonic mucosal barrier (Figure 10E). In conclusion, compound **F14** exhibits significant in vivo anti-inflammatory activity in the DSS-induced mouse colitis model, alleviating colitis symptoms and protecting colonic tissue.

## 3. Discussion

In this study, we synthesized a series of novel chalcone derivatives and evaluated their in vivo and in vitro activities. Our results show that most of the compounds were effective in inhibiting IL-1β secretion, with compound **F14** standing out as the most potent inhibitor, exhibiting IC_50_ values of 0.74 μM in BMDM and 0.88 μM in THP-1 cells. These findings suggest that **F14** may be a promising candidate for further development as an anti-inflammatory agent.

The mechanism of action for **F14** was explored through various assays, which revealed that **F14** did not affect mitochondrial ROS production or mitochondrial damage, indicating that its activity does not rely on mitochondrial dysfunction. Furthermore, **F14** did not affect the expression of key protein components of the NLRP3 inflammasome, which suggests that its anti-inflammatory effects might not involve direct modulation of inflammasome protein expression. Further research has found that **F14** may inhibit the activation of NLRP3 inflammasome by targeting the NLRP3 protein to block ASC oligomerization and spot formation. The mechanism of action of **F14** is similar to previously reported licochalcone B, but **F14** has stronger activity [42]. The in vivo data further support **F14**’s therapeutic potential, as it demonstrated significant therapeutic effects in a mouse model of DSS-induced acute colitis.

Based on the molecular structure of **F14**, we can anticipate favorable drug-likeness features. Chalcone derivatives, in general, tend to exhibit good oral bioavailability due to their relatively small size, planar structure, and the presence of functional groups that could facilitate absorption. Additionally, **F14**’s moderate molecular weight and lipophilicity suggest it may have adequate permeability for cellular uptake. However, further in vitro and in vivo studies assessing its pharmacokinetic properties, such as solubility, metabolic stability, and half-life, would be necessary to fully evaluate its ADME profile. We believe **F14** shows promise as a lead compound for further optimization, and these additional studies will be pursued as part of future work.

## 4. Materials and Methods

### 4.1. Chemistry

The reaction progress was monitored by thin-layer chromatography (TLC) using pre-coated silica gel GF254 plates. ^1^H NMR and ^13^C NMR spectra were recorded on a Bruker AVANCE-III −400 MH_Z_ (^1^H, 400 MHz; ^13^C, 100 MHz) spectrometer, with DMSO-*d*_6_ as the solvent and tetramethylsilane (TMS) as the internal standard. Chemical shifts (*δ*) were reported in ppm, and coupling constants (*J*) were given in Hz. High-resolution mass spectrometry (HRMS) was obtained by using an Orbitrap Exploris 480 (Thermo Fisher Scientific, Waltham, MA, USA) mass spectrometer in electrospray ionization (ESI) mode. Compounds with high activity have their structures reconfirmed by infrared spectroscopy.

### 4.2. Chemical Synthesis

#### 4.2.1. Procedure for the Synthesis of **W1**–**W11**

Synthesis of compound **W1**: Benzaldehyde (4.09 mmol, 1.0 equivalent) was weighed and dissolved in 10 mL of anhydrous ethanol, and a magnetic stirrer was added for stirring. Phenylacetone (4.91 mmol, 1.2 equivalent) was then added slowly in multiple aliquots. After thorough mixing, NaOH (15 mmol, 4.15 equivalent) was added, and the reaction mixture was stirred for 30 min. The color of the solution changed from colorless to brownish-yellow, and new product spots were observed. After an overnight reaction at room temperature, the reaction was monitored by TLC, with product spots showing strong fluorescence under UV wavelengths of 256 and 365 nm. Upon completion of the reaction, the mixture was transferred to a separatory funnel, and dilute hydrochloric acid and ethyl acetate were added and shaken to induce phase separation. The organic phase was then washed twice with a saturated saline solution. Anhydrous sodium sulfate was added for drying, and the product was obtained by reduced-pressure distillation. The product was purified by silica gel column chromatography (EtOAc/PE = 1/6, *v*/*v* gradient elution), yielding a pale-yellow solid.

*(E)-3-(4-hydroxyphenyl)-1-phenylprop-2-en-1-one* **(W1)** white solid; yield 74.1%; ^1^H NMR (400 MHz, DMSO-*d*_6_) *δ* 10.13 (s, 1H), 8.17–8.09 (m, 2H), 7.74 (dd, *J* = 14.2, 7.5 Hz, 4H), 7.70–7.61 (m, 1H), 7.57 (t, *J* = 7.6 Hz, 2H), 6.85 (d, *J* = 8.3 Hz, 2H). ^13^C NMR (101 MHz, DMSO-*d*_6_) *δ* 189.45, 160.64, 145.01, 138.41, 133.30, 131.53, 129.20, 128.81, 126.23, 118.92, 116.29. HR-MS(ESI): calcd for C_15_H_12_O_2_[M + H]^+^, 229.03304, found, 230.0837.

The synthesis route of compounds **W2**–**W11** is the same as **W1**.

*(E)-3-(4-hydroxyphenyl)-1-(3-methoxyphenyl)propyl-2-en-1-one* **(W2)** yellow solid; yield 72.5%; 1H NMR (400 MHz, DMSO-*d*_6_) *δ* 10.17 (s, 1H), 7.80 (d, *J* = 8.5 Hz, 3H), 7.78–7.74 (m, 2H), 7.64 (t, *J* = 2.0 Hz, 1H), 7.52 (t, *J* = 7.9 Hz, 1H), 7.27 (dd, *J* = 8.1, 2.6 Hz, 1H), 6.89 (d, *J* = 8.3 Hz, 2H), 3.90 (s, 3H). ^13^C NMR (101 MHz, DMSO-*d*_6_) *δ* 189.16, 160.65, 159.98, 145.09, 139.89, 131.59, 130.32, 126.23, 121.32, 119.35, 118.96, 116.31, 116.28, 113.30, 55.81. HR-MS(ESI): calcd for C_16_H_13_O_3_[M + H]^+^, 253.08702, found, 254.0943.
*(E)-1-(3-chlorophenyl)-3-(4-hydroxyphenyl)propyl-2-en-1-one* **(W3)** light yellow solid; yield 64.5%; ^1^H NMR (400 MHz, DMSO-*d*_6_) *δ* 10.18 (s, 1H), 8.17 (t, *J* = 1.9 Hz, 1H), 8.09 (dt, *J* = 7.8, 1.4 Hz, 1H), 7.82–7.77 (m, 2H), 7.74 (d, *J* = 4.7 Hz, 2H), 7.73–7.69 (m, 1H), 7.60 (t, *J* = 7.9 Hz, 1H), 7.00–6.80 (m, 2H). ^13^C NMR (101 MHz, DMSO-*d*_6_) *δ* 188.11, 160.86, 145.86, 140.26, 134.25, 133.02, 131.81, 131.17, 128.48, 127.44, 126.15, 118.48, 116.29. HR-MS(ESI): calcd for C_15_H_10_O_2_Cl[M]^+^, 257.03757, found, 257.0369.
*(E)-3-(4-hydroxyphenyl)-1-(4-methoxyphenyl)propyl-2-en-1-one* **(W4)** yellow solid; yield 69.3%; ^1^H NMR (400 MHz, DMSO-*d*_6_) *δ* 10.07 (s, 1H), 8.21–8.11 (m, 2H), 7.78–7.71 (m, 3H), 7.64 (d, *J* = 15.5 Hz, 1H), 7.15–7.02 (m, 2H), 6.90–6.71 (m, 2H), 3.87 (s, 3H). ^13^C NMR (101 MHz, DMSO-*d*_6_) *δ* 187.68, 163.45, 160.41, 144.09, 131.36, 131.23, 131.18, 126.38, 118.86, 116.24, 114.41, 56.00. HR-MS(ESI): calcd for C_16_H_13_O_3_[M + H]^+^, 253.08708, found, 254.0943.
*(E)-1-(2-chlorophenyl)-3-(4-hydroxyphenyl)propion-2-en-1-one* **(W5)** white solid; yield 55.4%; ^1^H NMR (400 MHz, DMSO-*d*_6_) *δ* 10.20 (s, 1H), 7.61 (d, *J* = 8.6 Hz, 2H), 7.59–7.53 (m, 2H), 7.52 (s, 1H), 7.50–7.45 (m, 1H), 7.29 (d, *J* = 16.0 Hz, 1H), 7.04 (d, *J* = 16.0 Hz, 1H), 6.81 (d, *J* = 8.6 Hz, 2H). ^13^C NMR (101 MHz, DMSO-*d*_6_) *δ* 193.65, 161.05, 147.50, 139.53, 131.96, 131.60, 130.46, 130.24, 129.54, 127.80, 125.52, 123.45, 116.42. HR-MS(ESI): calcd for C_15_H_10_O_2_Cl[M + H]^+^, 257.03757, found, 258.0448.
*(E)-1-(4-bromophenyl)-3-(4-hydroxyphenyl)propyl-2-en-1-one* **(W6)** yellow solid; yield 61.3%; ^1^H NMR (400 MHz, DMSO-*d*_6_) *δ* 10.16 (d, *J* = 6.0 Hz, 1H), 8.07 (t, *J* = 7.1 Hz, 2H), 7.77 (d, *J* = 6.5 Hz, 3H), 7.75–7.67 (m, 3H), 6.85 (t, *J* = 6.8 Hz, 2H). ^13^C NMR (101 MHz, DMSO-*d*_6_) *δ* 188.50, 160.78, 145.55, 137.39, 132.23, 131.66, 130.87, 127.40, 126.16, 118.55, 116.31. HR-MS(ESI): calcd for C_15_H_10_O_2_Br[M + H]^+^, 300.98709, found, 301.9942.
*(E)-1-(4-chlorophenyl)-3-(4-hydroxyphenyl)propyl-2-en-1-one* **(W7)** white solid; yield 67.5%; ^1^H NMR (400 MHz, DMSO-*d*_6_) *δ* 10.15 (s, 1H), 8.26–8.08 (m, 2H), 7.82–7.69 (m, 4H), 7.67–7.58 (m, 2H), 6.91–6.77 (m, 2H). ^13^C NMR (101 MHz, DMSO-*d*_6_) *δ* 188.30, 160.78, 145.51, 138.23, 137.07, 131.64, 130.73, 129.27, 126.17, 118.57, 116.31. HR-MS(ESI): calcd for C_15_H_10_O_2_Cl[M]^+^, 257.03763, found, 257.0369.
*(E)-1-(2,4-dimethylphenyl)-3-(4-hydroxyphenyl)propyl-2-en-1-one* **(W8)** yellow solid; yield 77.1%; ^1^H NMR (400 MHz, DMSO-*d*_6_) *δ* 10.12 (s, 1H), 7.77 (d, *J* = 8.6 Hz, 1H), 7.61 (d, *J* = 8.3 Hz, 2H), 7.51 (d, *J* = 8.2 Hz, 1H), 7.38 (d, *J* = 15.8 Hz, 1H), 7.17 (d, *J* = 15.9 Hz, 1H), 6.94 (d, *J* = 8.3 Hz, 1H), 6.82 (d, *J* = 8.3 Hz, 2H), 2.34 (s, 3H), 2.32 (s, 3H). ^13^C NMR (101 MHz, DMSO-*d*_6_) *δ* 194.95, 191.40, 163.79, 160.58, 145.41, 140.73, 136.97, 136.81, 132.57, 132.34, 131.24, 128.95, 128.89, 126.65, 125.94, 123.41, 116.33, 116.30, 21.36, 20.51. HR-MS(ESI): calcd for C_17_H_15_O_2_[M + H]^+^, 251.10783, found, 252.1150.
*(E)-1-(3,4-dimethylphenyl)-3-(4-hydroxyphenyl)propyl-2-en-1-one* **(W9)** white solid; yield 64.9%; ^1^H NMR (400 MHz, DMSO-*d*_6_) *δ* 10.46–10.19 (m, 1H), 8.08 (d, *J* = 15.7 Hz, 1H), 7.97–7.81 (m, 4H), 7.32 (dd, *J* = 20.9, 7.8 Hz, 2H), 7.03–6.87 (m, 2H), 2.35 (s, 3H), 2.33 (s, 3H). ^13^C NMR (101 MHz, DMSO-*d*_6_) *δ* 189.43, 157.66, 142.57, 139.38, 137.28, 136.21, 132.37, 130.26, 129.80, 129.03, 126.55, 121.95, 121.40, 119.82, 116.68, 20.06, 19.82. HR-MS(ESI): calcd for C_17_H_15_O_2_[M + H]^+^, 251.10785, found, 252.1150.
*(E)-1-(3-fluorophenyl)-3-(4-hydroxyphenyl)propyl-2-en-1-one* **(W10)** white solid; yield 78.5%; ^1^H NMR (400 MHz, DMSO-*d*_6_) *δ* 10.19 (s, 1H), 7.99 (dt, *J* = 7.7, 1.2 Hz, 1H), 7.96–7.90 (m, 1H), 7.82–7.75 (m, 2H), 7.74 (d, *J* = 1.7 Hz, 2H), 7.61 (td, *J* = 8.0, 5.8 Hz, 1H), 7.50 (td, *J* = 8.5, 2.4 Hz, 1H), 6.90–6.83 (m, 2H). ^13^C NMR (101 MHz, DMSO-*d*_6_) *δ* 188.15, 188.13, 164.02, 161.59, 160.85, 145.76, 140.72, 140.65, 131.75, 131.37, 131.29, 126.14, 124.98, 124.95, 120.28, 120.06, 118.51, 116.31, 115.46, 115.24. HR-MS(ESI): calcd for C_15_H_12_O_2_F[M + H]^+^, 241.06705, found, 242.0742.
*(E)-3-(4-hydroxyphenyl)-1-(naphthalene-1-yl)propyl-2-en-1-one* **(W11)** light yellow solid; yield 79.2%; ^1^H NMR (400 MHz, DMSO-*d*_6_) *δ* 10.18 (s, 1H), 8.30–8.21 (m, 1H), 8.12 (d, *J* = 8.2 Hz, 1H), 8.06–7.99 (m, 1H), 7.89 (dd, *J* = 7.1, 1.2 Hz, 1H), 7.64 (dt, *J* = 8.4, 2.5 Hz, 3H), 7.59 (ddd, *J* = 7.8, 3.5, 1.9 Hz, 2H), 7.52 (d, *J* = 15.8 Hz, 1H), 7.34 (d, *J* = 15.9 Hz, 1H), 6.89–6.79 (m, 2H). ^13^C NMR (101 MHz, DMSO-*d*_6_) *δ* 194.95, 160.80, 146.29, 137.33, 133.86, 131.72, 131.48, 130.35, 128.96, 127.80, 127.64, 126.88, 125.85, 125.73, 125.42, 123.91, 116.38. HR-MS(ESI): calcd for C_19_H_13_O_2_[M + H]^+^, 273.09216, found, 274.0994.

#### 4.2.2. Procedure for the Synthesis of **F1**–**F16**

Synthesis of compound **F1** series: Benzaldehyde (4.09 mmol, 1.0 equivalent) was weighed and dissolved in 10 mL of anhydrous ethanol, with a magnetic stirrer added for stirring. Phenylacetone (4.91 mmol, 1.2 equivalent) was then added slowly in multiple aliquots. After thorough mixing, NaOH (15 mmol, 4.15 equivalent) was added, and the reaction mixture was stirred for 30 min. The color of the solution changed from colorless to brownish-yellow, and new product spots were formed. After an overnight reaction at room temperature, TLC was performed, and product spots showed strong fluorescence under UV wavelengths of 256 and 365 nm. Upon completion of the reaction, the mixture was transferred to a separatory funnel, and dilute hydrochloric acid and ethyl acetate were added and shaken to induce phase separation. The organic phase was then washed twice with a saturated saline solution. Anhydrous sodium sulfate was added for drying, and the product was obtained by reduced-pressure distillation. The product was purified by silica gel column chromatography (EtOAc/PE = 1/6, *v*/*v* gradient elution), yielding a pale-yellow solid.

*(E)-3-(4-hydroxyphenyl)-1-phenylpropyl-2-en-1-one* **(F1)** white solid; yield 46.8%; ^1^H NMR (400 MHz, DMSO-*d*_6_) *δ* 10.30 (s, 1H), 8.20–7.97 (m, 3H), 7.97–7.76 (m, 2H), 7.62 (dt, *J* = 37.2, 7.4 Hz, 3H), 7.38–7.11 (m, 1H), 7.05–6.77 (m, 2H). ^13^C NMR (101 MHz, DMSO-*d*_6_) *δ* 189.95, 157.72, 139.99, 138.37, 133.39, 132.57, 129.26, 129.17, 128.82, 121.80, 121.35, 119.89, 116.69. HR-MS(ESI): calcd for C_15_H_11_O_2_[M + H]^+^, 223.07651, found,223.07651.

The synthesis route of compounds **F2**–**F16** is the same as **F1**.

*(E)-3-(2-hydroxyphenyl)-1-(3-methoxyphenyl)propyl-2-en-1-one* **(F2)** yellow solid; yield 70.2%; ^1^H NMR (400 MHz, DMSO-*d*_6_) *δ* 9.69 (s, 1H), 7.85 (d, *J* = 15.6 Hz, 1H), 7.78 (d, *J* = 7.6 Hz, 1H), 7.71–7.59 (m, 2H), 7.50 (t, *J* = 8.0 Hz, 1H), 7.35 (d, *J* = 7.7 Hz, 1H), 7.31–7.22 (m, 3H), 6.91 (dd, *J* = 7.8, 2.4 Hz, 1H), 3.87 (s, 3H). ^13^C NMR (101 MHz, DMSO-*d*_6_) *δ* 189.42, 160.01, 158.20, 144.85, 139.51, 136.39, 130.40, 130.35, 122.39, 121.51, 120.43, 119.67, 118.33, 115.80, 113.39, 55.82. HR-MS(ESI): calcd for C_16_H_13_O_3_[M + H]^+^, 253.08705, found, 254.0943.
*(E)-3-(3-hydroxyphenyl)-1-(3-methoxyphenyl)propyl-2-en-1-one* (**F3**) white solid; yield 68.5%; ^1^H NMR (400 MHz, DMSO-*d*_6_) *δ* 10.36 (s, 1H), 8.11 (d, *J* = 15.8 Hz, 1H), 7.97–7.85 (m, 2H), 7.76 (d, *J* = 7.6 Hz, 1H), 7.62 (t, *J* = 2.1 Hz, 1H), 7.54 (t, *J* = 7.9 Hz, 1H), 7.35–7.27 (m, 2H), 6.99 (d, *J* = 8.2 Hz, 1H), 6.93 (t, *J* = 7.5 Hz, 1H), 3.90 (s, 3H). ^13^C NMR (101 MHz, DMSO-*d*_6_) *δ* 189.67, 159.99, 157.72, 140.04, 139.83, 132.59, 130.40, 129.17, 121.80, 121.36, 121.33, 119.88, 119.44, 116.67, 113.29, 55.81. HR-MS(ESI): calcd for C_16_H_13_O_3_[M + H]^+^, 253.08711, found, 254.0943.
*(E)-3-(3-hydroxyphenyl)-1-phenylpropyl-2-en-1-one* (**F4**) white solid; yield 68.3%;^1^H NMR (400 MHz, DMSO-*d*_6_) *δ* 9.67 (s, 1H), 8.30–8.03 (m, 2H), 7.85 (d, *J* = 15.6 Hz, 1H), 7.78–7.54 (m, 4H), 7.41–7.12 (m, 3H), 6.89 (dd, *J* = 8.0, 2.4 Hz, 1H). ^13^C NMR (101 MHz, DMSO-*d*_6_) *δ* 189.68, 144.77, 138.04, 136.39, 133.61, 130.37, 129.27, 128.98, 122.35, 120.36, 118.31, 115.76. HR-MS(ESI): calcd for C_15_H_11_O_2_[M + H]^+^, 223.07675, found, 224.0837.
*(E)-3-(2-hydroxyphenyl)-1-(4-methoxyphenyl)propyl-2-en-1-one* (**F5**) white solid; yield 77.4%; ^1^H NMR (400 MHz, DMSO-*d*_6_) *δ* 10.25 (s, 1H), 8.25–7.95 (m, 3H), 7.97–7.79 (m, 2H), 7.41–7.05 (m, 3H), 7.00–6.82 (m, 2H), 3.87 (s, 3H). ^13^C NMR (101 MHz, DMSO-*d*_6_) *δ* 188.10, 163.51, 157.58, 139.06, 132.30, 131.19, 129.04, 121.97, 121.24, 119.85, 116.65, 114.47, 56.01. HR-MS(ESI): calcd for C_16_H_13_O_3_[M + H]^+^, 253.08720, found, 254.0943.
*(E)-3-(3-hydroxyphenyl)-1-(4-methoxyphenyl)propyl-2-en-1-one* (**F6**) yellow solid; yield 55.3%; ^1^H NMR (400 MHz, DMSO-*d*_6_) *δ* 9.64 (s, 1H), 8.27–8.08 (m, 2H), 7.85 (d, *J* = 15.6 Hz, 1H), 7.62 (d, *J* = 15.5 Hz, 1H), 7.37–7.21 (m, 3H), 7.15–6.81 (m, 3H), 3.87 (s, 3H). ^13^C NMR (101 MHz, DMSO-*d*_6_) *δ* 187.84, 163.68, 158.19, 143.89, 136.55, 131.40, 130.90, 130.33, 122.28, 120.24, 118.08, 115.68, 114.49, 56.04. HR-MS(ESI): calcd for C_16_H_13_O_3_S[M + H]^+^, 253.08720, found, 254.0943.
*(E)-1-(2-chlorophenyl)-3-(2-hydroxyphenyl)propyl-2-en-1-one* (**F7**) white solid; yield 43.5%; 1H NMR (400 MHz, DMSO-*d*_6_) *δ* 10.32 (s, 1H), 7.74–7.63 (m, 2H), 7.62–7.52 (m, 4H), 7.51–7.46 (m, 1H), 7.28 (td, *J* = 7.8, 7.3, 1.7 Hz, 1H), 7.23 (d, *J* = 16.3 Hz, 1H), 6.95–6.82 (m, 2H).^13^C NMR (101 MHz, DMSO-*d*_6_) *δ* 194.21, 157.67, 142.63, 139.45, 133.09, 132.06, 130.47, 130.25, 129.58, 129.36, 127.82, 125.95, 121.13, 120.03, 116.73. HR-MS(ESI): calcd for C_15_H_10_O_2_Cl[M + H]^+^, 257.03763, found, 258.0448.
*(E)-1-(4-chlorophenyl)-3-(2-hydroxyphenyl)propyl-2-en-1-one* (**F8**) yellow solid; yield 74.1%; ^1^H NMR (400 MHz, DMSO-*d*_6_) *δ* 10.34 (s, 1H), 8.24–8.00 (m, 3H), 7.92–7.83 (m, 2H), 7.68–7.61 (m, 2H), 7.29 (td, *J* = 7.8, 7.3, 1.7 Hz, 1H), 6.97–6.85 (m, 2H). ^13^C NMR (101 MHz, DMSO-*d*_6_) *δ* 188.78, 157.83, 140.42, 137.01, 132.74, 130.76, 129.36, 129.20, 121.72, 120.92, 119.87, 116.71. HR-MS(ESI): calcd for C_15_H_10_O_2_Cl[M + H]^+^, 257.03769, found, 258.0448.
*(E)-1-(4-bromophenyl)-3-(2-hydroxyphenyl)propyl-2-en-1-one* (**F9**) white solid; yield 48.9%;^1^H NMR (400 MHz, DMSO-*d*_6_) *δ* 10.34 (s, 1H), 8.27–8.02 (m, 3H), 8.02–7.71 (m, 4H), 7.30 (ddd, *J* = 8.6, 7.2, 1.7 Hz, 1H), 7.10–6.78 (m, 2H). ^13^C NMR (101 MHz, DMSO-*d*_6_) *δ* 188.98, 157.82, 140.45, 137.33, 132.74, 132.30, 130.87, 129.21, 127.50, 121.72, 120.91, 119.88, 116.71. HR-MS(ESI): calcd for C_15_H_10_O_2_Br[M + H]^+^, 300.98712, found, 301.9942.
*(E)-1-(4-bromophenyl)-3-(3-hydroxyphenyl)propyl-2-en-1-one* (**F10**) white solid; yield 71.2%; ^1^H NMR (400 MHz, DMSO-*d*_6_) *δ* 9.66 (s, 1H), 8.09 (d, *J* = 8.3 Hz, 2H), 7.91–7.74 (m, 3H), 7.67 (d, *J* = 15.6 Hz, 1H), 7.39–7.21 (m, 3H), 6.89 (dd, *J* = 7.9, 2.4 Hz, 1H). ^13^C NMR (101 MHz, DMSO-*d*_6_) *δ* 188.79, 158.21, 145.27, 137.02, 136.31, 132.31, 131.02, 130.36, 127.76, 122.01, 120.47, 118.45, 115.83. HR-MS(ESI): calcd for C_15_H_10_O_2_Br[M + H]^+^, 300.98727, found, 301.9942.
*(E)-1-(2,4-dimethylphenyl)-3-(2-hydroxyphenyl)propyl-2-en-1-one* (**F11**) white solid; yield 58.4%; ^1^H NMR (400 MHz, DMSO-*d*_6_) *δ* 10.26 (s, 1H), 7.82–7.69 (m, 2H), 7.50 (d, *J* = 8.2 Hz, 1H), 7.39–7.21 (m, 2H), 7.12 (d, *J* = 5.5 Hz, 2H), 6.94 (d, *J* = 8.2 Hz, 1H), 6.85 (t, *J* = 7.5 Hz, 1H), 2.35 (s, 3H), 2.32 (s, 3H). ^13^C NMR (101 MHz, DMSO-*d*_6_) *δ* 195.48, 157.51, 140.82, 140.64, 137.01, 136.74, 132.45, 132.35, 129.10, 128.96, 126.65, 125.96, 121.62, 119.93, 116.67, 21.35, 20.52. HR-MS(ESI): calcd for C_17_H_15_O_2_[M + H]^+^, 251.10788, found, 252.1150.
*(E)-1-(3,4-dimethylphenyl)-3-(3-hydroxyphenyl)propyl-2-en-1-one* (**F12**) white solid; yield 64.5%; ^1^H NMR (400 MHz, DMSO-*d*_6_) *δ* 9.66 (s, 1H), 7.95 (d, *J* = 1.9 Hz, 1H), 7.92–7.78 (m, 2H), 7.63 (d, *J* = 15.6 Hz, 1H), 7.39–7.30 (m, 2H), 7.29–7.20 (m, 2H), 6.93–6.78 (m, 1H), 2.32 (s, 3H), 2.30 (s, 3H). ^13^C NMR (101 MHz, DMSO-*d*_6_) *δ* 189.13, 158.20, 144.19, 142.87, 137.36, 136.50, 135.88, 130.33, 130.30, 129.95, 126.71, 122.38, 120.32, 118.17, 115.67, 20.09, 19.79. HR-MS(ESI): calcd for C_17_H_15_O_2_[M + H]^+^, 251.10782, found, 252.1150.
*(E)-1-(3-fluorophenyl)-3-(3-hydroxyphenyl)propyl-2-en-1-one* (**F13**) yellow solid; yield 73.1%; ^1^H NMR (400 MHz, DMSO-*d*_6_) *δ* 10.34 (s, 1H), 8.11 (d, *J* = 15.8 Hz, 1H), 7.97 (d, *J* = 7.7 Hz, 1H), 7.95–7.82 (m, 3H), 7.63 (td, *J* = 7.9, 5.7 Hz, 1H), 7.52 (td, *J* = 8.5, 2.6 Hz, 1H), 7.35–7.26 (m, 1H), 7.00–6.85 (m, 2H). ^13^C NMR (101 MHz, DMSO-*d*_6_) *δ* 188.65, 188.62, 164.02, 161.58, 157.83, 140.55, 132.82, 131.46, 131.38, 129.11, 125.02, 124.99, 121.69, 120.82, 119.87, 116.70, 115.45, 115.23. HR-MS(ESI): calcd for C_15_H_10_O_2_F[M + H]^+^, 241.06702, found, 242.0743.
*(E)-1-(2,4-dimethylphenyl)-3-(3-hydroxyphenyl)propyl-2-en-1-one* (**F14**) white solid; yield 61.9%; ^1^H NMR (400 MHz, DMSO-*d*_6_) *δ* 9.66 (s, 1H), 7.59 (d, *J* = 8.3 Hz, 1H), 7.35 (q, *J* = 15.9 Hz, 2H), 7.22 (dt, *J* = 14.9, 7.8 Hz, 2H), 7.17–7.10 (m, 3H), 6.87 (dt, *J* = 8.0, 1.8 Hz, 1H), 2.37 (s, 3H), 2.33 (s, 3H). ^13^C NMR (101 MHz, DMSO-*d*_6_) *δ* 194.58, 158.21, 144.89, 141.28, 137.44, 136.23, 136.19, 132.52, 130.42, 129.38, 126.75, 126.40, 120.18, 118.27, 115.36. HR-MS(ESI): calcd for C_17_H_15_O_2_[M + H]^+^, 251.10789, found, 252.1150.
*(E)-3-(2-hydroxyphenyl)-1-(naphthale-1-yl)propyl-2-en-1-one* (**F15**) white solid; yield 46.5%; ^1^H NMR (400 MHz, DMSO-*d*_6_) *δ* 10.31 (s, 1H), 8.30–8.22 (m, 1H), 8.13 (d, *J* = 8.2 Hz, 1H), 8.08–8.00 (m, 1H), 7.92–7.83 (m, 2H), 7.76 (dd, *J* = 7.9, 1.7 Hz, 1H), 7.61 (qd, *J* = 7.5, 6.5, 2.9 Hz, 3H), 7.50 (s, 1H), 7.28 (ddd, *J* = 8.4, 7.3, 1.6 Hz, 1H), 6.94 (d, *J* = 8.2 Hz, 1H), 6.87 (t, *J* = 7.3 Hz, 1H). ^13^C NMR (101 MHz, DMSO-*d*_6_) *δ* 195.45, 157.65, 141.43, 137.28, 133.85, 132.75, 131.81, 130.34, 129.30, 128.99, 127.86, 127.68, 126.93, 126.44, 125.72, 125.40, 121.49, 119.98, 116.71. HR-MS(ESI): calcd for C_19_H_13_O_2_[M + H]^+^, 273.09229, found, 274.0994.
*(E)-3-(2-hydroxyphenyl)-1-(thiophene-2-yl)propion-2-en-1-one* (**F16**) yellow solid; yield 61.1%; ^1^H NMR (400 MHz, DMSO-*d*_6_) *δ* 10.25 (s, 1H), 8.18 (d, *J* = 3.8 Hz, 1H), 8.04–7.95 (m, 2H), 7.82 (dd, *J* = 7.8, 1.7 Hz, 1H), 7.74 (d, *J* = 15.7 Hz, 1H), 7.28–7.18 (m, 2H), 6.88 (d, *J* = 8.2 Hz, 1H), 6.82 (t, *J* = 7.5 Hz, 1H). ^13^C NMR (101 MHz, DMSO-*d*_6_) *δ* 182.25, 157.72, 146.18, 138.95, 135.65, 133.59, 132.63, 129.37, 128.99, 121.63, 121.02, 119.86, 116.71. HR-MS(ESI): calcd for C_13_H_9_O_2_S[M + H]^+^, 229.03304, found, 230.0402.

### 4.3. Cell Culture

L929, HepaRG, J774A.1, and THP-1 were all purchased from the Shanghai Cell Bank, Chinese Academy of Sciences. THP-1 cells were cultured in RPMI-1640 medium as the base medium, while the other cells were cultured in DMEM high-glucose medium (containing 10% fetal bovine serum and 1% penicillin-streptomycin). All cells were cultured in a cell incubator at 37 °C with 5% CO_2_.

### 4.4. Isolation, Extraction, and Cultivation of BMDMs

When L929 cells reach the logarithmic phase, collect the cell supernatant. Filter the supernatant using a 0.22 μm filter and store it at −20 °C for future use. The 7-week-old C57BL/6 mice were selected as the source of primary bone marrow macrophages. The mice were euthanized using the cervical dislocation method. The ends of the femurs were cut, and the bone marrow was flushed into a 15 mL centrifuge tube using a 5 mL syringe. Once all the bone marrow was collected, an appropriate amount of red blood cell lysis buffer was added to the centrifuge tube. The mixture was incubated for 3–5 min, with repeated pipetting to thoroughly lyse the red blood cells. The cells were then centrifuged and collected. Add 30–40% L929 cell supernatant or M-CSF stimulatory factor to the culture dish. Culture the cells in a cell incubator at 37 °C with 5% CO_2_. Observe the cell condition daily, and once most cells have adhered to the surface, proceed with subsequent experiments.

### 4.5. MTT Assay

HepaRG cells in the logarithmic growth phase (10^5^ cells/mL), inoculate them into a 96-well plate, and culture overnight in a 37 °C, 5% CO_2_ incubator. Add compounds at different concentrations and continue culturing for 24 h. Add 20 μL of MTT solution, incubate for 4 h, discard the supernatant, and add DMSO to dissolve. Measure the absorbance of each well at a wavelength of 492 nm using a microplate reader, and calculate the IC_50_ value using SPSS 17.0 software.

### 4.6. The Effect of ELISA Detection Compounds on IL-1β Secretion

Inoculate 1.0 × 10^5^ BMDMs cells per well into a 96 well plate and culture overnight. Stimulate the cells with lipopolysaccharide (LPS) at a concentration of 0.2 μg/mL for 3 h, then add different concentrations of the test compounds and incubate for 30 min. Finally, add Nigericin at a concentration of 10 μM to continue the induction for 30 min. Collect the cell supernatant and measure the IL-1β secretion level in the supernatant using an ELISA kit, following the instructions provided, and calculate the IC_50_ value based on the relevant calculations. To ensure the accuracy and reliability of the experimental results, all experiments were repeated three times, with each experiment performed independently.

### 4.7. Western Blot Operation Is Detailed in the Reference Literature [39]

Inoculate BMDMs cells into a 6-well plate with 4-6 × 10^6^ cells per well and culture overnight. Pre-treat with LPS (200 ng/mL) for 3 h, then treat with different concentrations of compound **F14** for 30 min, and add Nigericin (10 µM) to activate BMDMs for 30 min. Add RIPA (Beyotime Biotechnology, Shanghai, China) for lysis. Select SDS-PAGE with different concentrations for electrophoresis, and then transfer to PVDF membrane (Millipore Immobilon-P, Burlington, MA, USA). Incubate the antibody imprint overnight at 4°C, and then incubate with HRP conjugated secondary antibody for 1 h.

### 4.8. ASC Spot Experiment

Add the BMDM cells into a 24-well plate with a cell density of 3 × 10^5^ cells per well. Add LPS (200 ng/mL) to all wells except the blank wells to stimulate the cells for 3 h, then add the **F14** to the experimental group and incubate for 1 h, followed by Nigericin stimulation for 30 min. Add 500 μL of FAA fixation solution to each well, incubate with 1% TritonX-100 solution for 15 min, then block with 5% BSA solution. Add an appropriate amount of primary antibody and incubate overnight, wash, then add the secondary antibody for incubation. Place the coverslips with the cell side down on a glass slide pre-coated with DAPI mounting medium, and observe the samples using a laser confocal microscope.

### 4.9. ROS Detection

BMDMs were seeded at a density of 1.0 × 10^6^ cells per well in a 6-well plate, and the cell treatment was performed as described above. DCFH-DA probe was added to each well at a 1:1000 dilution (prepared in serum-free medium), and the cells were incubated at 37 °C in the dark for 20 min. The cells were gently mixed every 5 min to ensure uniform probe penetration. The cells were washed three times with PBS, and ROS levels were measured using flow cytometry (excitation/emission wavelengths: 488/525 nm).

### 4.10. LDH Detection

Count the THP-1 cells and inoculate them into a 96-well plate. Add 50 ng/mL PMA and culture the cells at 37 °C in a 5% CO_2_ incubator for 24 h. Add LPS-containing medium and stimulate the cells for 3 h to induce an inflammatory response. Add **F14** and incubate for 1 h, then add Nigericin and stimulate for 30 min to induce pyroptosis. Perform the assay according to the instructions of the LDH assay kit. Calculate the inhibition rate of the compounds on pyroptosis, and further calculate the IC_50_ value using SPSS 17.0 software.

### 4.11. Docking

Molecular docking was performed using Autodock Vina 1.1.2. First, the complex structure of NLRP3 and ATP (PDB ID:6npy) was downloaded from the PDB database and pretreated (the PDB source file removed water molecules/cofactors, and the protonation state was optimized through Chimera hydrogenation), and the 3D conformation of small molecules was generated using Open Babel. The docking box is defined based on the position of the original ligand ATP. The docking grid size is set to 20 × 20 × 20 Å^3^ to ensure sufficient sampling space. Specify the core parameters when running docking: search intensity (exhaustiveness = 32), output bound conformation number, and energy range = 5 kcal/mol. The docking results were sorted according to the binding energy, and the best conformational cluster was screened. The reliability of the binding mode was verified by ligand-receptor interaction analysis. Finally, PyMOL (3.1) was used to visualize the structure of the high score complex.

### 4.12. Molecular Dynamics Simulations

Molecular dynamics simulations of the NLRP3-**F14** complex were performed using GROMACS (v2024). The CHARMM36m force field is first adopted to describe the protein, the small molecule ligands are parameterized by GAFF2 force fields, and the compatible TIP3P water model is selected. The complex was placed in a dodecahedral solvent box, with the box boundary 1.0 nm from the protein surface, and NaCl ions were added to achieve system electrical neutrality. First, the steepest gradient descent and the L-BFGS algorithm were used to minimize the energy to resolve spatial conflicts. A two-step equilibration phase was then performed: (**1**) 100 ps NVT equilibration at 310 K with V-rescale thermostating and protein heavy-atom restraints, and (**2**) 1-ns NPT equilibration at 1 bar using the Parrinello-Rahman barostat. Finally, a 100 ns production dynamics simulation (nsteps = 50,000,000) was conducted, employing leap-frog integration under NPT ensemble conditions controlled by a Parrinello-Rahman barostat. Non-bonded interactions were handled using PME electrostatics with a 1.2-nm van der Waals cutoff. Bond constraints were maintained using the LINCS algorithm, and trajectories were saved every 10 ps.

### 4.13. In Vivo Pharmacological Experiments

Male C57BL/6 mice, 18–20 g, were purchased from Charles River (China). All experiments and animal care procedures were approved by the Animal Resource Center of Anhui Medical University by the National Institutes of Health Guide for the Care and Use of Laboratory Animals (20230875). This study used DSS induction to establish an acute colitis model in mice. Mice in the experimental group were given free access to a 3% DSS solution for 7 days to induce an intestinal inflammation phenotype. The positive control group (sulfasalazine, 200 mg/kg) and the treatment group were gavage administration daily, using a 0.5% carboxymethyl cellulose sodium (CMC-Na) solution as the drug vehicle. The normal control and model groups received an equal volume of blank CMC-Na solution. Daily monitoring of weight changes and clinical symptoms was performed during the experimental period, including fecal characteristics (formed/loose/watery stools) and the degree of rectal bleeding (occult blood/gross blood in feces). On day 10, at the end of the experiment, the colonic sections of the mice were carefully excised. The excised colon was examined, and the length of the colon was measured. Some colon tissues were fixed in 4% formaldehyde and used for HE staining and immunohistochemical analysis for subsequent evaluations.

## 5. Conclusions

**Figure 11:**
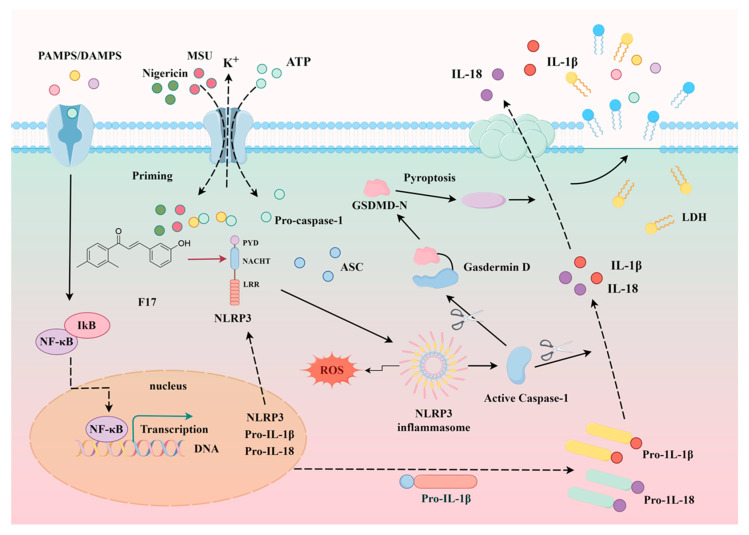
Mechanism diagram of compound **F14**.

In this study, a series of chalcone derivatives was designed and synthesized. After screening for activity, most of the compounds exhibited good anti-inflammatory activity, with **F14** showing the most prominent inhibition of IL-1β. In vitro studies revealed that compound **F14** exerts anti-inflammatory activity by targeting NLRP3 and inhibiting ASC speck formation (Figure 11). In vivo studies showed that **F14** alleviates DSS-induced acute colitis, reducing tissue damage and providing protection against colitis. In conclusion, **F14** exhibited significant anti-inflammatory activity in both in vitro cell models and in vivo animal models. These results support further chemical development of **F14** as a promising lead compound for NLRP3 inhibition and explore its therapeutic potential for inflammation and related diseases.

## Figures and Tables

**Table 1:** Synthesis of compounds **W1**–**W11**.

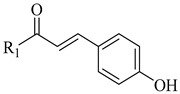				
Compounds	R_1_		Compounds	R_1_
**W1**	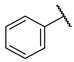		**W7**	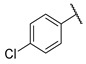
**W2**	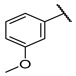		**W8**	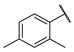
**W3**	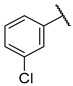		**W9**	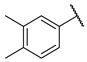
**W4**	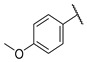		**W10**	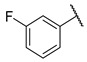
**W5**	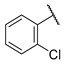		**W11**	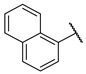
**W6**	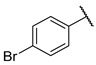			

**Table 2:** Synthesis of compounds **F1**–**F16**.

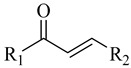					
Compounds	R_1_	R_2_	Compounds	R_1_	R_2_
**F1**	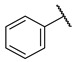	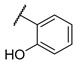	**F9**	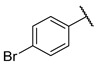	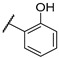
**F2**	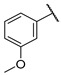	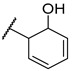	**F10**	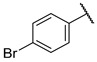	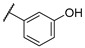
**F3**	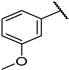	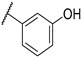	**F11**	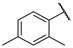	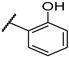
**F4**	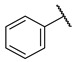	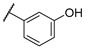	**F12**	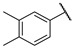	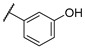
**F5**	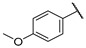	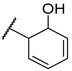	**F13**	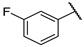	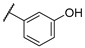
**F6**	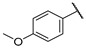	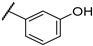	**F14**	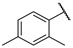	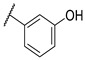
**F7**	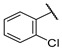	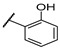	**F15**	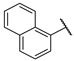	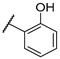
**F8**	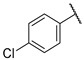	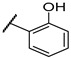	**F16**	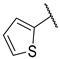	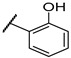

**Table 3:** Inhibition rate of **W** series compounds on IL-1β in BMDM cells.

Compound	IL-1β Inhibition Rate (%) ^a^	HepaRG Cytotoxicity ^a^
	20 µM	IC_50_ (µM)
**W1**	17.77 ± 3.45	>50
**W2**	67.03 ± 1.23	>50
**W3**	21.84 ± 9.56	>50
**W4**	6.89 ± 4.78	>50
**W5**	51.74 ± 2.23	>50
**W6**	27.65 ± 8.12	>50
**W7**	24.31 ± 3.67	>50
**W8**	88.86 ± 5.91	>50
**W9**	10.33 ± 6.43	>50
**W10**	33.65 ± 3.21	>50
**W11**	88.31 ± 7.01	>50
**MCC950**	99.47 ± 0.12	>50

^a^ Data are presented as the mean ± S.E.M. (*n* = 3).

**Table 4:** Inhibition rate of F-series compounds on IL-1β in BMDM cells.

Compound	IL-1β Inhibition Rate (%) ^a^	HepaRG Cytotoxicity ^a^
	20 µM	IC_50_ (µM)
**F1**	52.07 ± 3.72	>50
**F2**	92.01 ± 2.15	>50
**F3**	93.42 ± 2.98	>50
**F4**	25.58 ± 9.03	>50
**F5**	93.92 ± 4.61	>50
**F6**	25.08 ± 2.84	>50
**F7**	94.75 ± 3.29	>50
**F8**	45.93 ± 3.45	>50
**F9**	28.41 ± 5.76	>50
**F10**	24.16 ± 7.91	>50
**F11**	88.71 ± 9.22	>50
**F12**	15.45 ± 4.13	>50
**F13**	34.01 ± 8.74	>50
**F14**	95.44 ± 2.56	>50
**F15**	91.68 ± 5.89	>50
**F16**	92.64 ± 3.23	>50
**MCC950**	99.47 ± 0.12	>50

^a^ Data are presented as the mean ± S.E.M. (*n* = 3).

## Data Availability

Data are contained within the article and Supplementary Materials.

## Supplementary Materials

^1^H NMR, ^13^C NMR, and HR-MS spectra data are provided as Supporting Information and can be downloaded at: https://www.mdpi.com/article/10.3390/molecules30163340/s1, Figure S1: The RMSF changes of **F14** and NLRP3 protein.

## Abbreviations

THF, tetrahydrofuran; HPLC, high-performance liquid chromatography; IL-1β, interleukin-1β; IBD, Inflammatory bowel disease; IL-6, interleukin-6; IR, inhibitory rate; LPS, lipopolysaccharide; TNF-α, tumor necrosis factor alpha; IC_50_, half maximal inhibitory concentration; ^1^H NMR, proton nuclear magnetic resonance; ^13^C NMR, carbon nuclear magnetic resonance; HR-MS, high-resolution electron impact mass spectra; DMF, N, N-dimethylformamide; MeOH, methanol; EtOAc, ethyl acetate; HCl, hydrochloric acid; DMSO, dimethyl sulfoxide.
