# Supplementary material for: Discovery of (E)-1,3-Diphenyl-2-Propen-1-One Derivatives as Potent and Orally Active NLRP3 Inflammasome Inhibitors for Colitis
Source: Molecules. 2025 Aug 11;30(16):3340. doi: 10.3390/molecules30163340 (PMC12388496; doi:10.3390/molecules30163340)
Supplement: Supplementary file 1 [file molecules-30-03340-s001.zip › molecules-3792071-supplementary.pdf]

Discovery of (*E*)-1,3-Diphenyl-2-Propen-1-One Derivatives as Potent and Orally Active NLRP3 Inflammasome Inhibitors for Colitis

Liuzeng Chen [1,2], Xiaoyu Zheng [1], Jiahui Li [1], Bin Zhou [1], Min Tao [1], Yuetian Yang [1], Yi Wang [1], Hao Zhan [1], Guoping Zhang [3], Jingbo Shi [2], Xingxing Zhang [1,*] and Banfeng Ruan [1,*]

[1] School of Biology, Food and Environment, Hefei University, Hefei 230601, China; clz@hfuu.edu.cn (L.C.); 13133517200@163.com (X.Z.); 18130626743@163.com (J.L.); zhoubin00163@163.com (B.Z.); 13696519598@163.com (M.T.); 18326671092@163.com (Y.Y.); wangyi__2023@163.com (Y.W.); 15385372606@163.com (H.Z.)

[2] School of Pharmacy, Anhui University of Chinese Medicine, Hefei 230601, China; sjbo616@126.com

[3] College of Chemistry and Materials Science, Huaibei Normal University, Huaibei 235000, China; hbzgp-1@163.com

* Correspondence: zhangxx@hfuu.edu.cn (X.Z.); ruanbf@hfuu.edu.cn (B.R.)

1. Analysis of RMSF value changes of **F14** and NLRP3 during simulation……S2

2. ^{1}H NMR, ^{13}C NMR, and HR-MS spectra for compounds **W1–W11** and **F1–F16**.………………………………………………………………S3–S56

We analyzed the RMSF changes of **F14** and NLRP3 protein (Supplementary Figure S1). The results indicated that the phenyl ring connected to the hydroxyl group exhibited significant fluctuations, likely due to the interactions between the hydroxyl group and protein residues, which cause pulling forces.

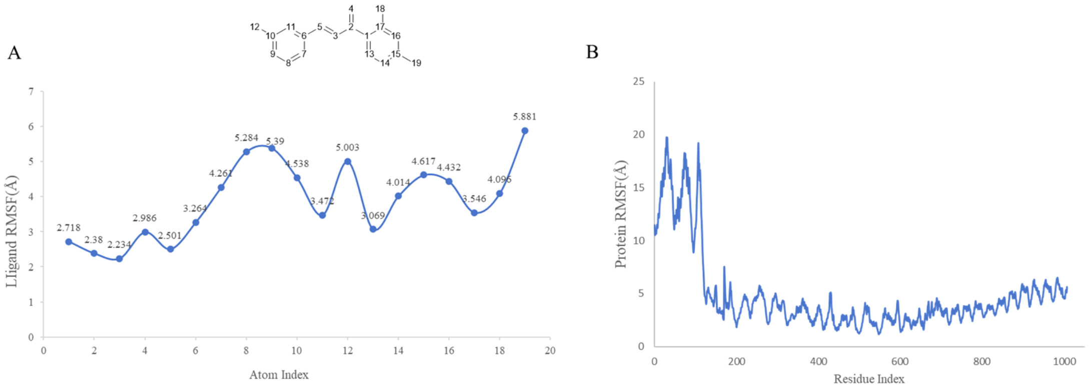

Supplementary Figure S1. The RMSF changes of **F14** and NLRP3 protein (**A**) RMSF changes of compound **F14; (B)** RMSF changes of NLRP3 protein.

(E)-3-(4-hydroxyphenyl)-1-phenylprop-2-en-1-one (W1)

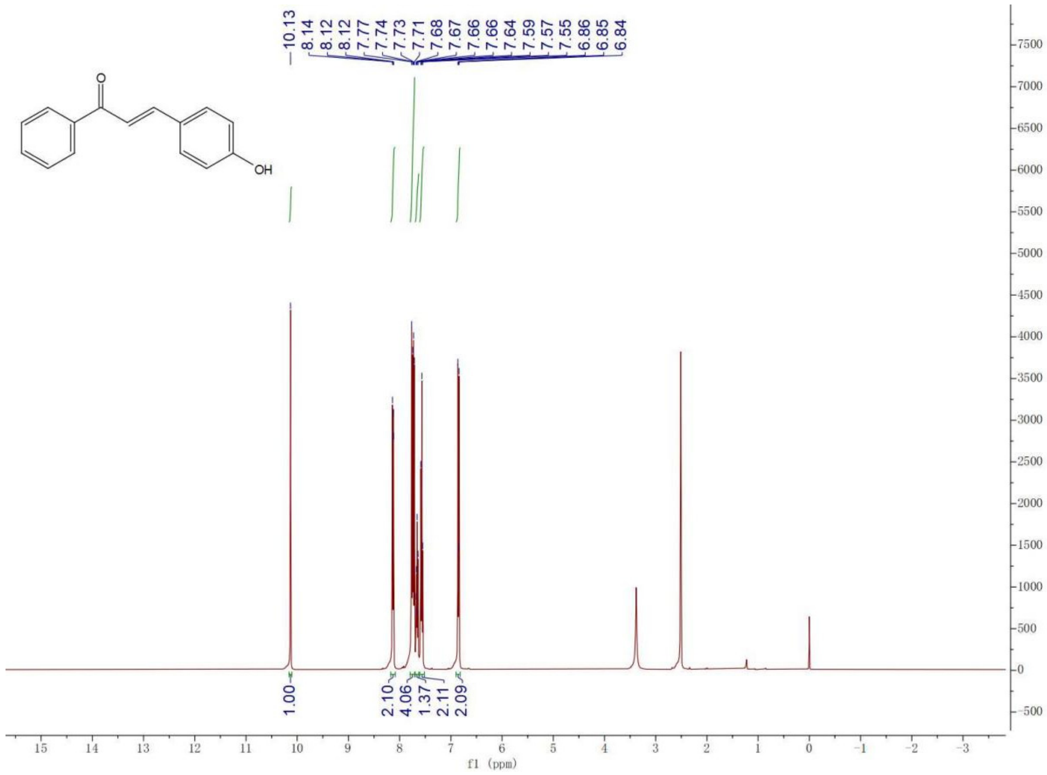

^{1}H NMR spectrum

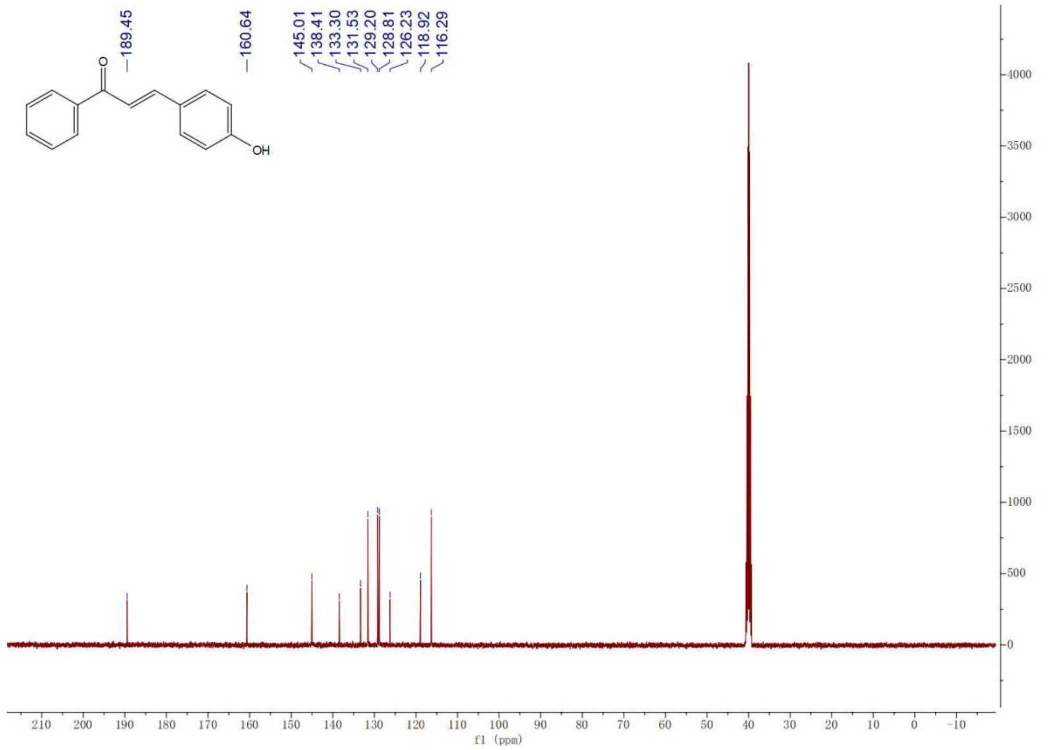

^{13}C NMR spectrum

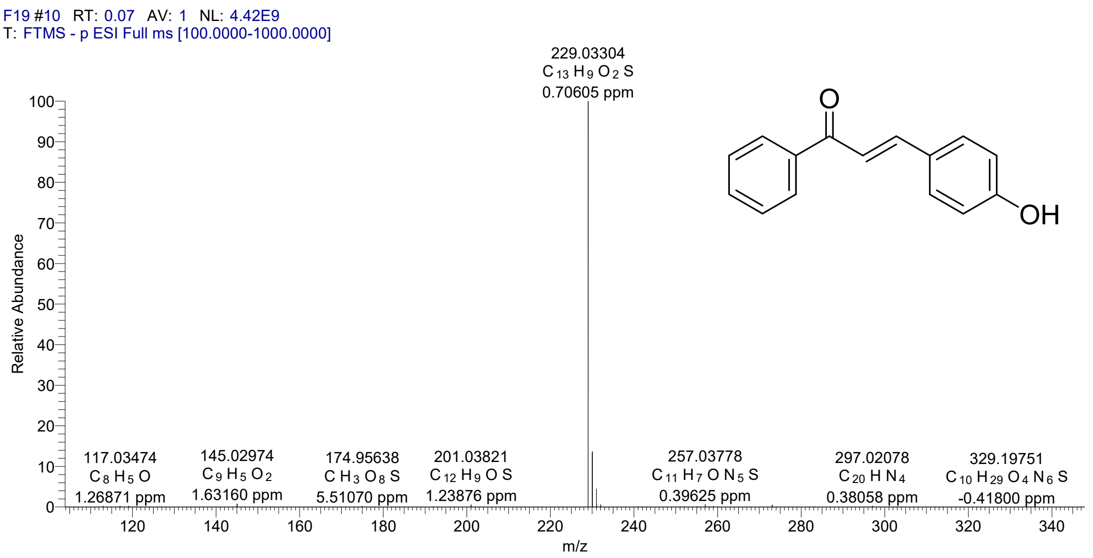

HRMS (ESI) spectrum

The C=O stretching vibration peak at 1649 cm^{-1} and the vibration peaks of O–H groups at 3225 cm^{-1}. Peaks at 1599, 1579, 1558, and 1511 cm^{-1} could be assigned to the stretching vibration of CH_2 in the aromatic nucleus. The peak at 833 cm^{-1} results from the out-of-plane bending vibration of C–H. The peaks at 1373 and 1322 cm^{-1} are ascribed to the stretching vibration of the C-C benzenoid ring. These observations confirmed that the structure is (*E*)-3-(4-hydroxyphenyl)-1-phenylprop-2-en-1-one (**W1**).

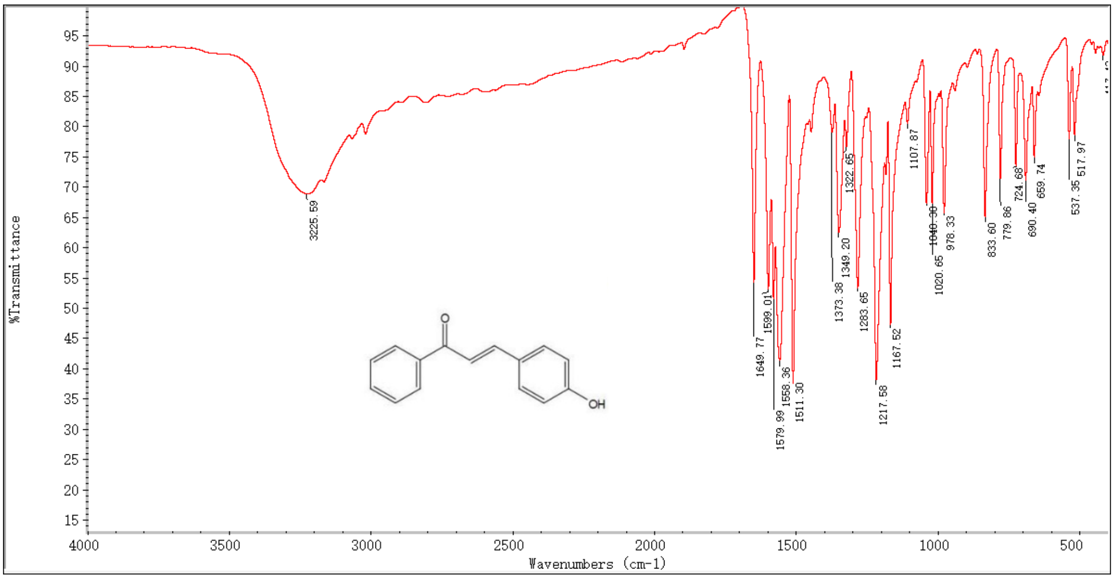

IR spectrum

(E)-3-(4-hydroxyphenyl)-1-(3-methoxyphenyl)propyl-2-en-1-one (W2)

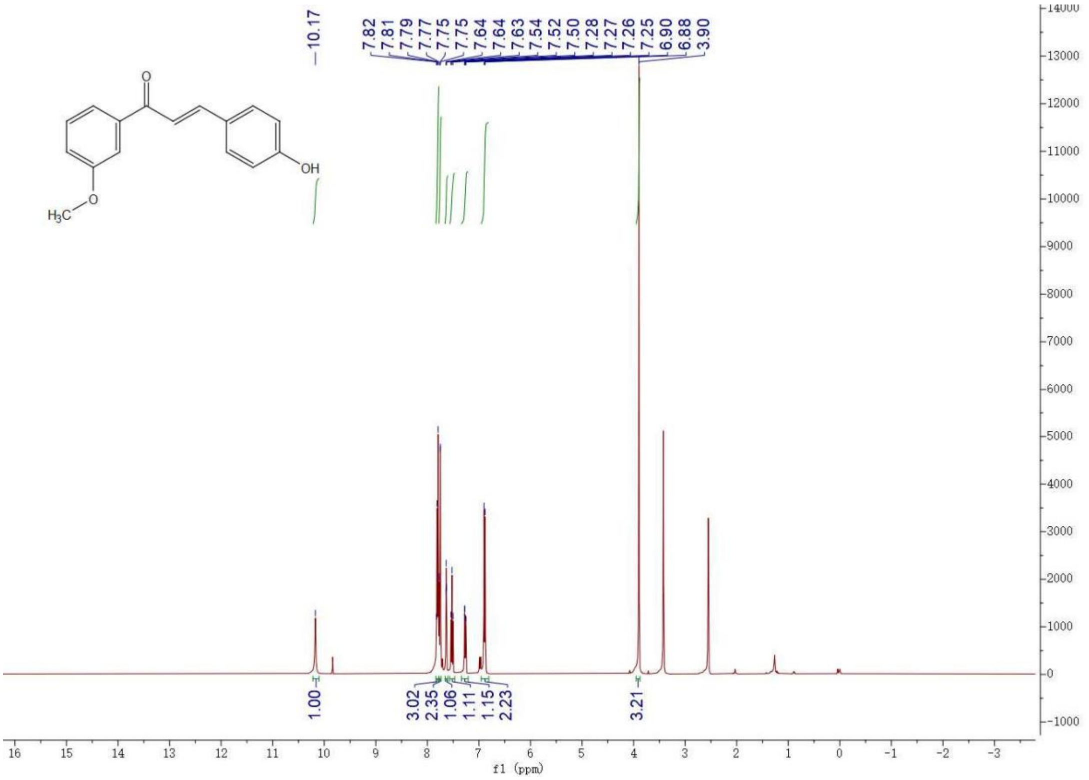

^1H NMR spectrum

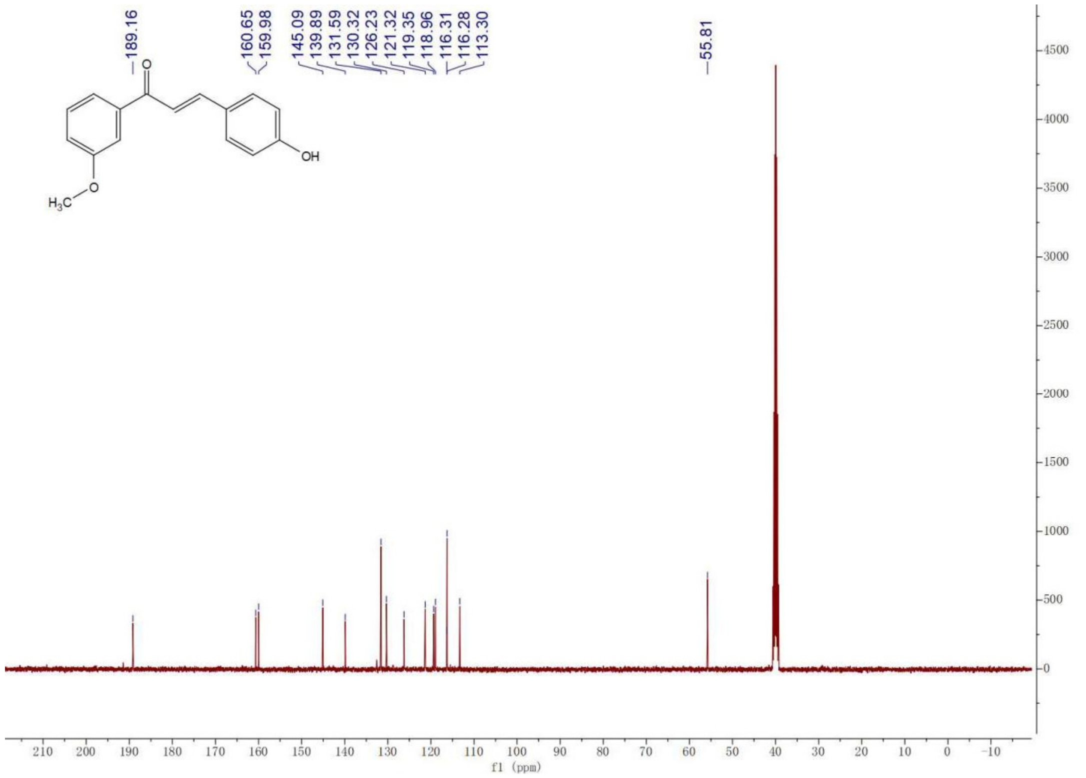

^{13}C NMR spectrum

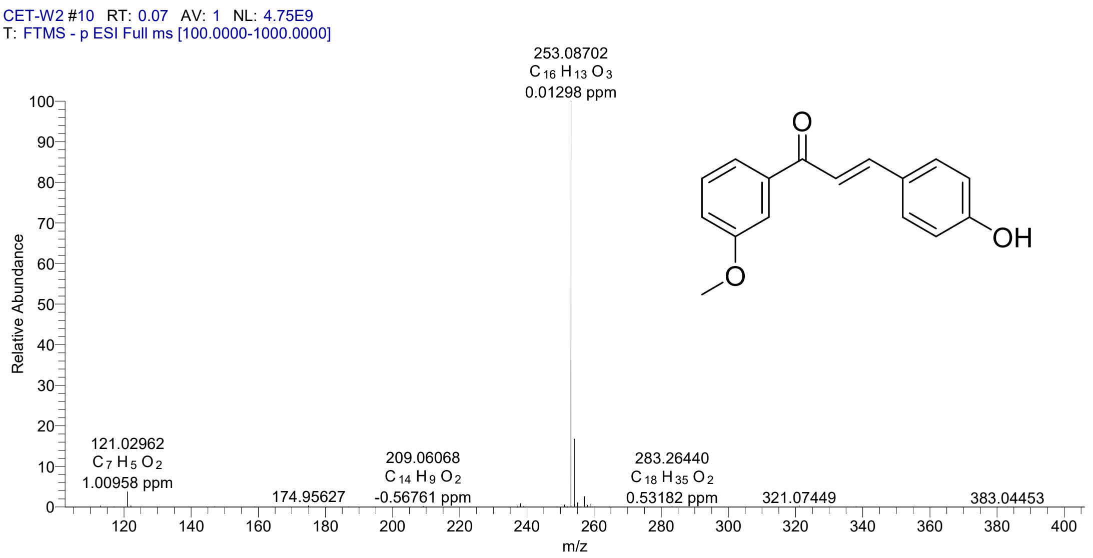

HRMS (ESI) spectrum

The C=O stretching vibration peak at 1651 cm^{-1} and the vibration peaks of O–H groups at 3314 cm^{-1}. The peaks at 3017 and 2967 cm^{-1} could be attributed to –OCH$_3$. Peaks at 1562, 1514, 1463, and 1435 cm^{-1} could be assigned to the stretching vibration of CH$_2$ in the aromatic nucleus. The peak at 830 cm^{-1} results from the out-of-plane bending vibration of C–H. The peaks at 1366 and 1335 cm^{-1} are ascribed to the stretching vibration of the C-C benzenoid ring. The peaks at 1103, 1064, and 1028 cm^{-1} are assigned to C–H of the methoxy group. These observations confirmed that the structure is (E)-3-(4-hydroxyphenyl)-1-(3-methoxyphenyl) propyl-2-en-1-one (**W2**).

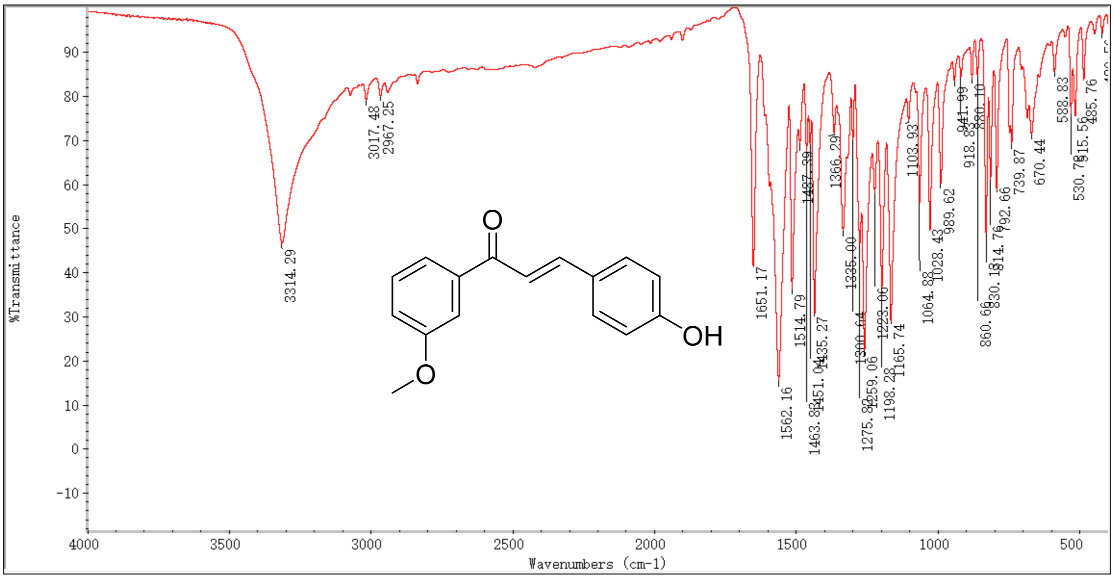

IR spectrum

***(E)-1-(3-chlorophenyl)-3-(4-hydroxyphenyl)propyl-2-en-1-one* (W3)**

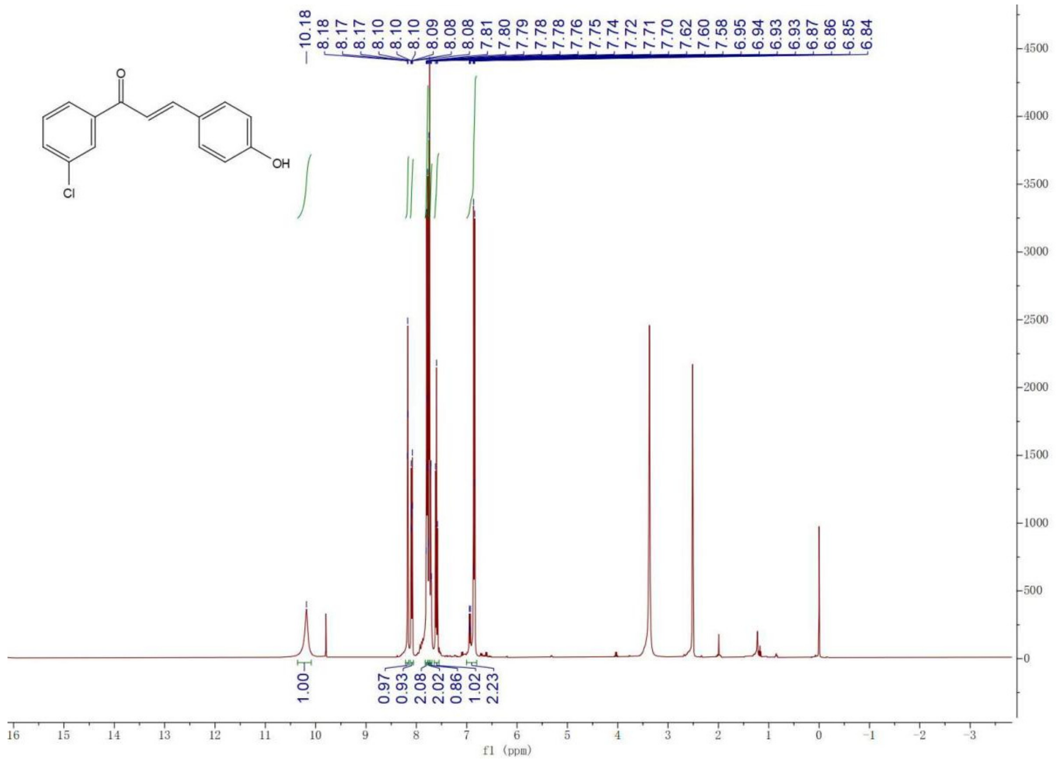

^{1}H NMR spectrum

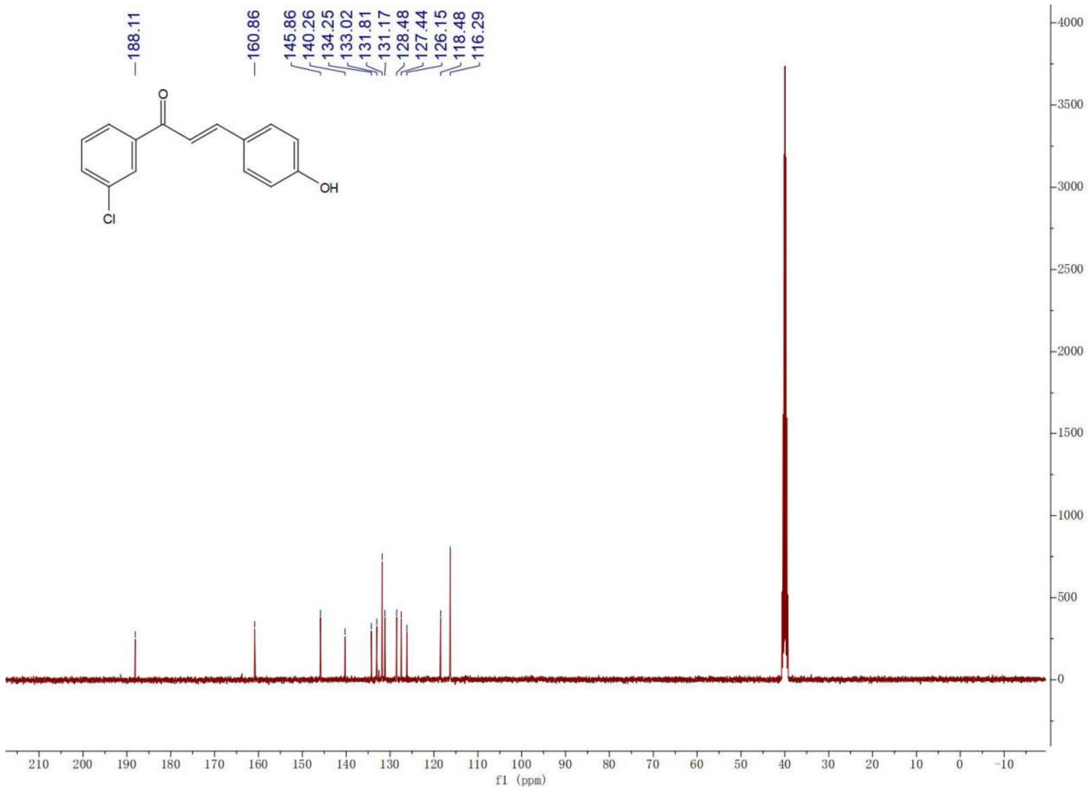

^{13}C NMR spectrum

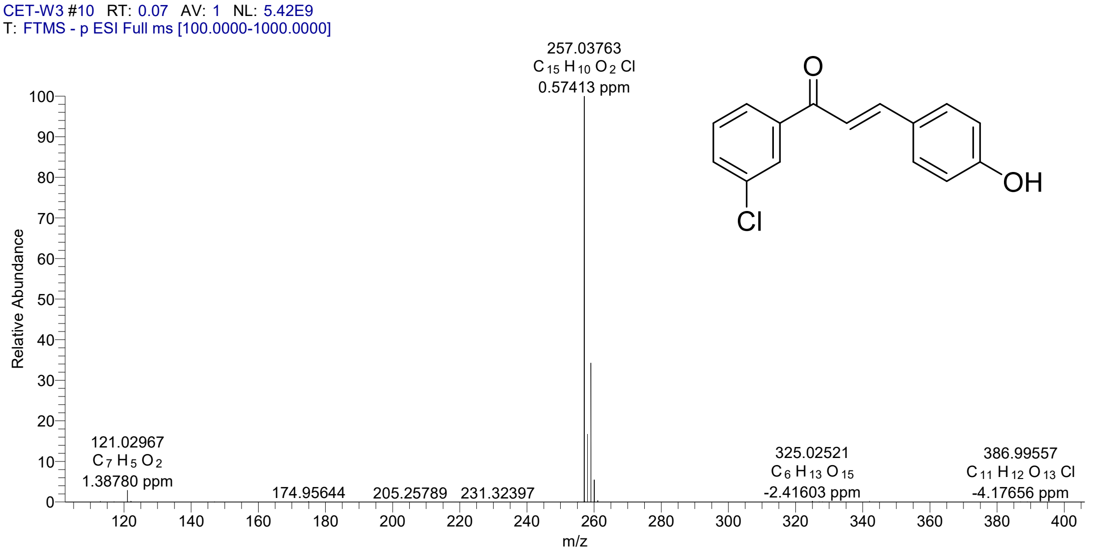

HRMS (ESI) spectrum

The C=O stretching vibration peak at 1660 cm^{-1} and the vibration peaks of O–H groups at 3372 cm^{-1}. Peaks at 1574, 1558, 1510, and 1440 cm^{-1} could be assigned to the stretching vibration of CH_2 in the aromatic nucleus. The peak at 826 cm^{-1} results from the out-of-plane bending vibration of C–H. The peaks at 1338 and 1299 cm^{-1} are ascribed to the stretching vibration of the C-C benzenoid ring. The peak at 792 cm^{-1} is assigned to the stretching vibration of the C-Cl. These observations confirmed that the structure is (E)-1-(3-chlorophenyl)-3-(4-hydroxyphenyl)propyl-2-en-1-one (**W3**).

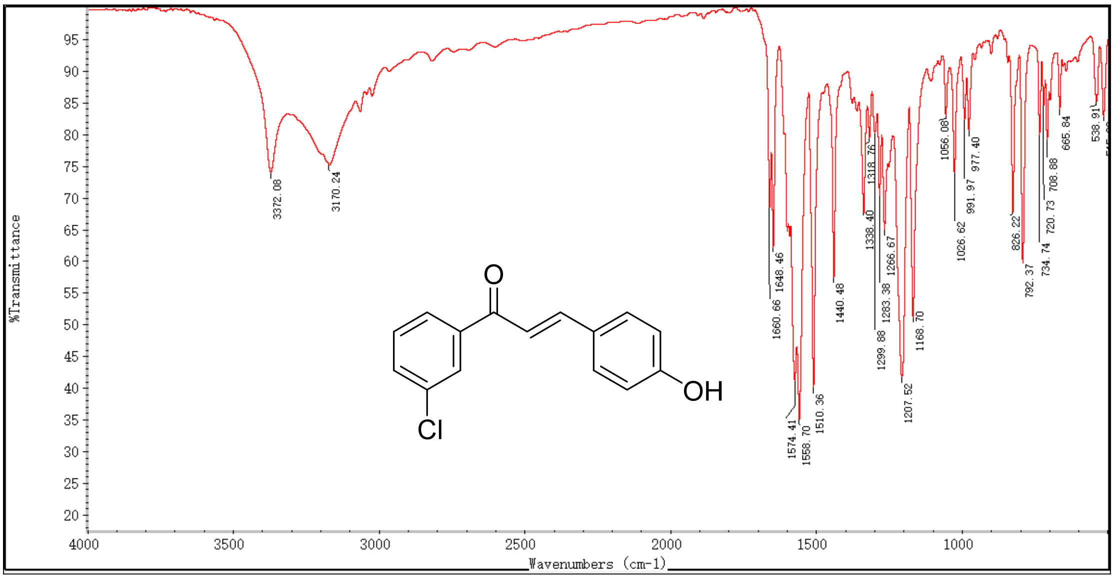

IR spectrum

(E)-3-(4-hydroxyphenyl)-1-(4-methoxyphenyl)propyl-2-en-1-one (W4)

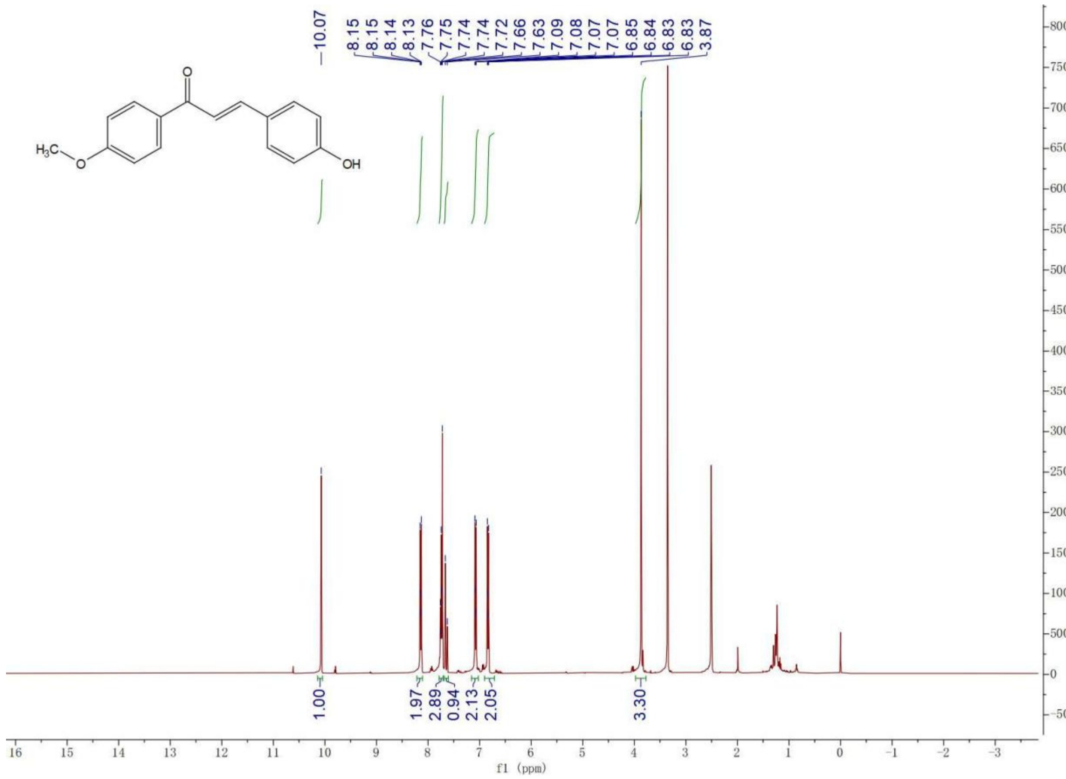

^{1}H NMR spectrum

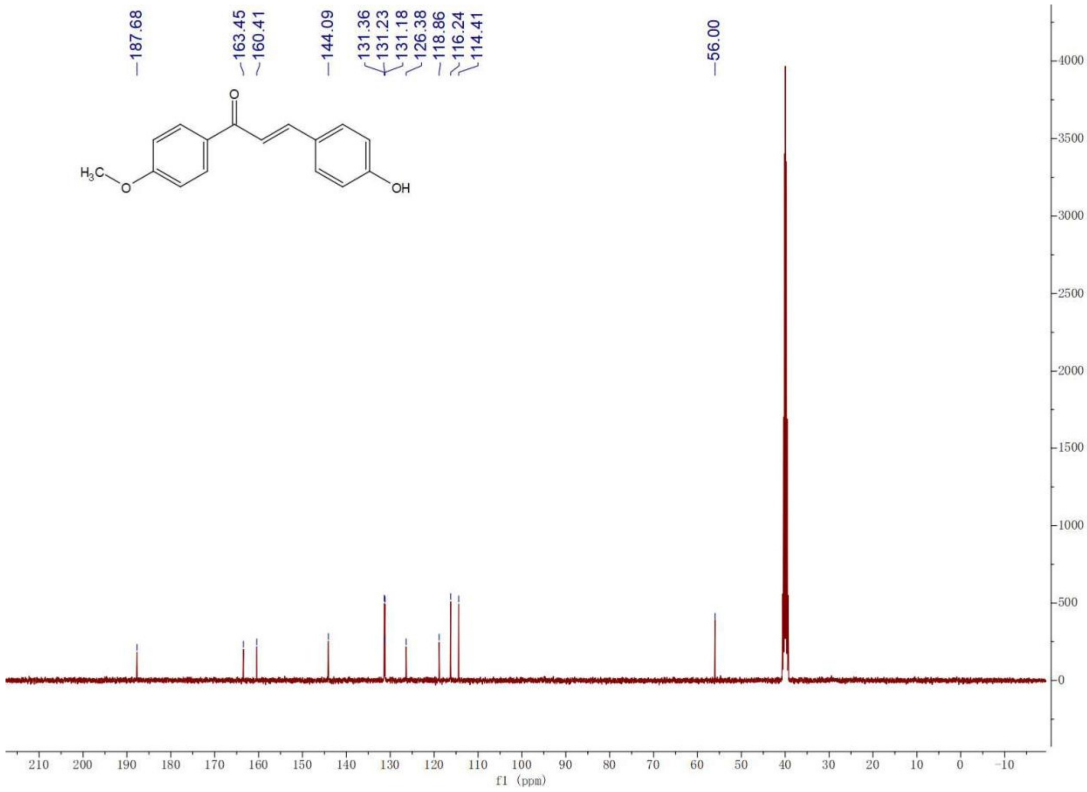

^{13}C NMR spectrum

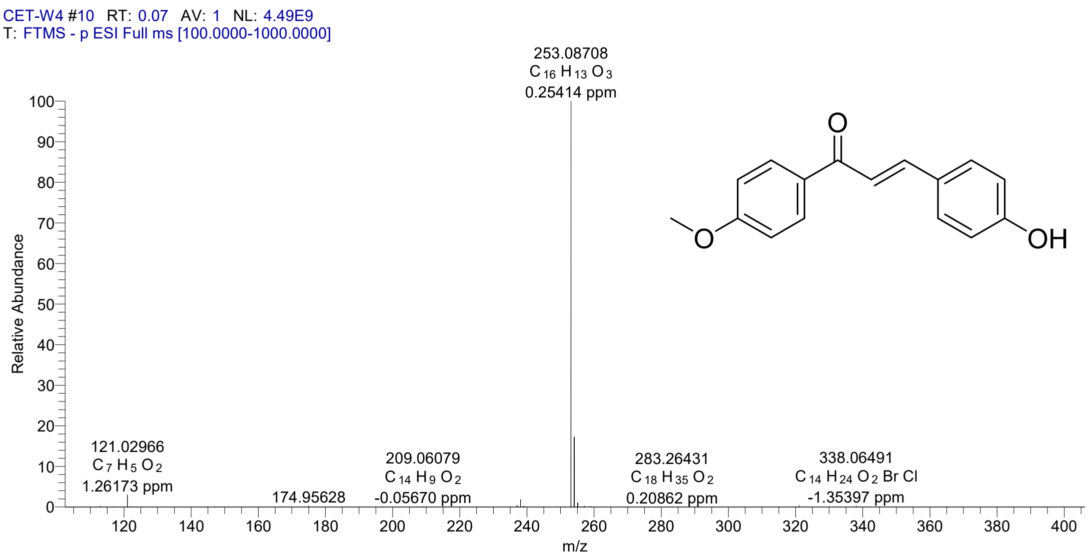

HRMS (ESI) spectrum

The C=O stretching vibration peak at 1643 cm^{-1} and the vibration peaks of O–H groups at 3158 cm^{-1}. The peaks at 2931 and 2835 cm^{-1} could be attributed to –OCH_3. Peaks at 1601, 1579, 1555, and 1513 cm^{-1} could be assigned to the stretching vibration of CH_2 in the aromatic nucleus. The peak at 830 cm^{-1} results from the out-of-plane bending vibration of C–H. The peaks at 1343 and 1322 cm^{-1} are ascribed to the stretching vibration of the C-C benzenoid ring. The peaks at 1036, 1021, and 977 cm^{-1} are assigned to C–H of the methoxy group. These observations confirmed that the structure is (E)-3-(4-hydroxyphenyl)-1-(4-methoxyphenyl)propyl-2-en-1-one (**W4**).

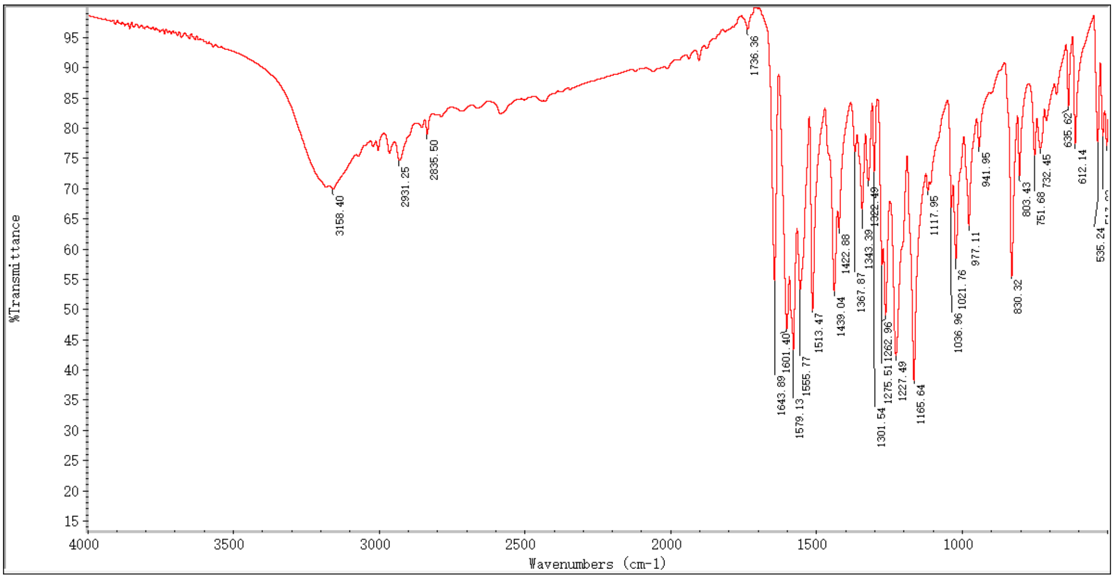

IR spectrum

(E)-1-(2-chlorophenyl)-3-(4-hydroxyphenyl)propion-2-en-1-one (W5)

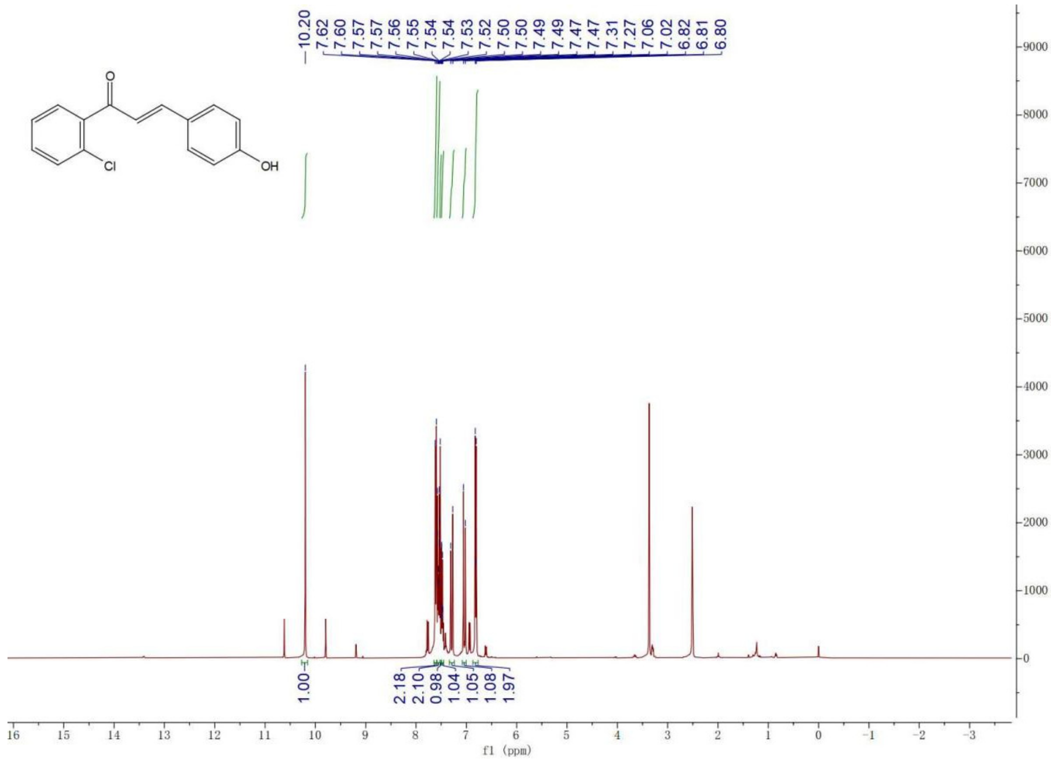

^{1}H NMR spectrum

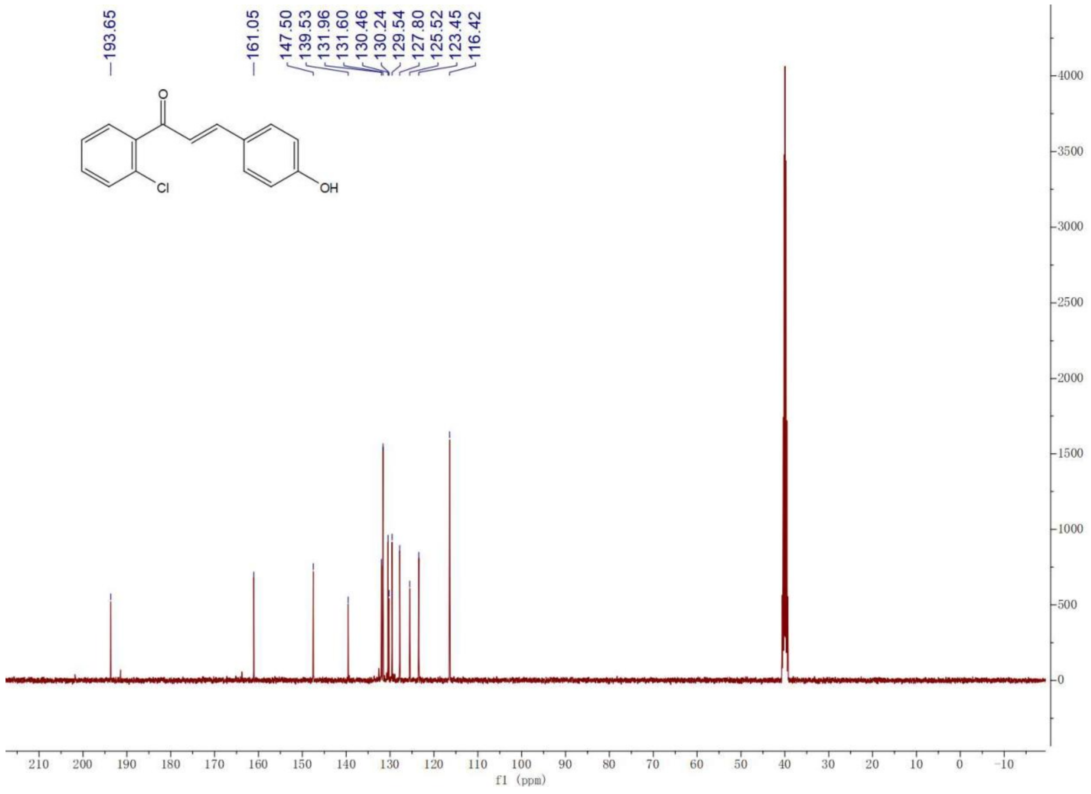

^{13}C NMR spectrum

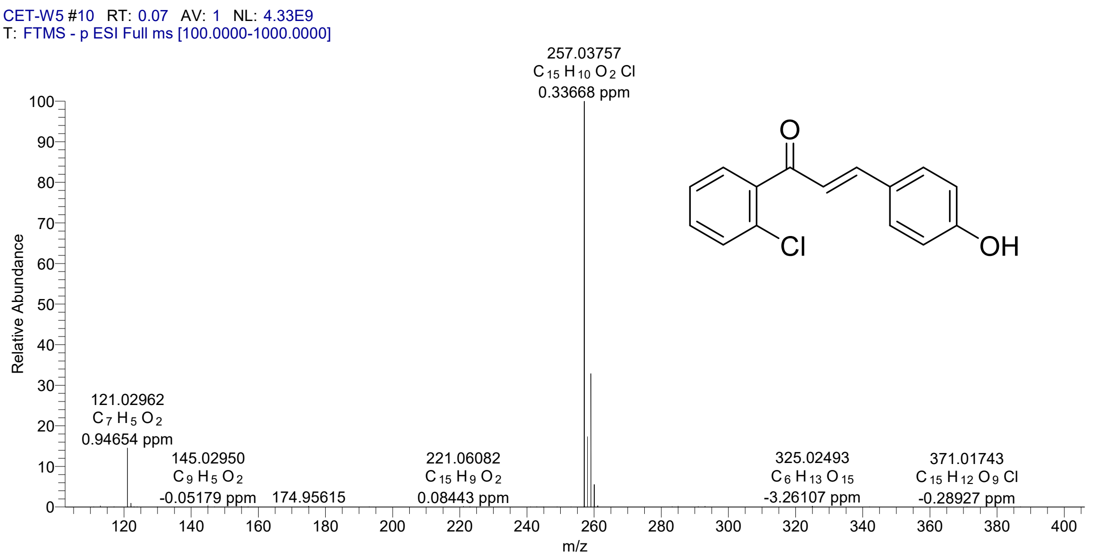

HRMS (ESI) spectrum

The C=O stretching vibration peak at 1650 cm-1 and the vibration peaks of O–H groups at 3361 cm-1. Peaks at 1592, 1554, 1512, and 1469 cm-1 could be assigned to the stretching vibration of CH_2 in the aromatic nucleus. The peak at 824 cm-1 results from the out-of-plane bending vibration of C–H. The peaks at 1372 and 1344 cm-1 are ascribed to the stretching vibration of the C-C benzenoid ring. The peak at 738 cm-1 is assigned to the stretching vibration of the C-Cl. These observations confirmed that the structure is (E)-1-(2-chlorophenyl)-3-(4-hydroxyphenyl)propion-2-en-1-one (**W5**).

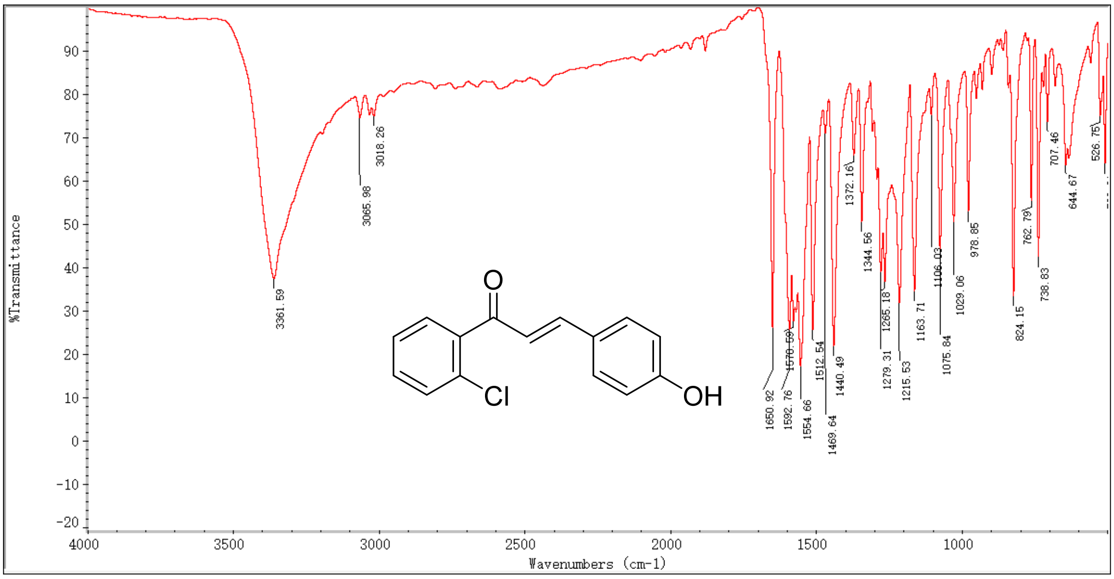

IR spectrum

(E)-1-(4-bromophenyl)-3-(4-hydroxyphenyl)propyl-2-en-1-one (W6)

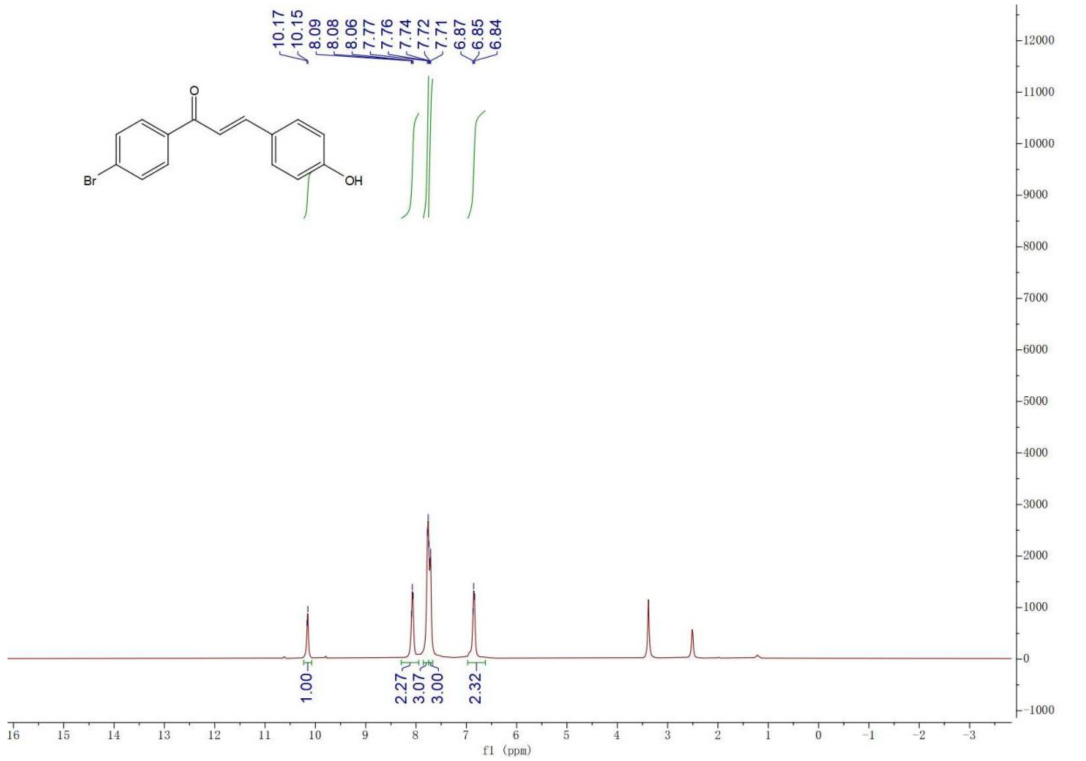

^{1}H NMR spectrum

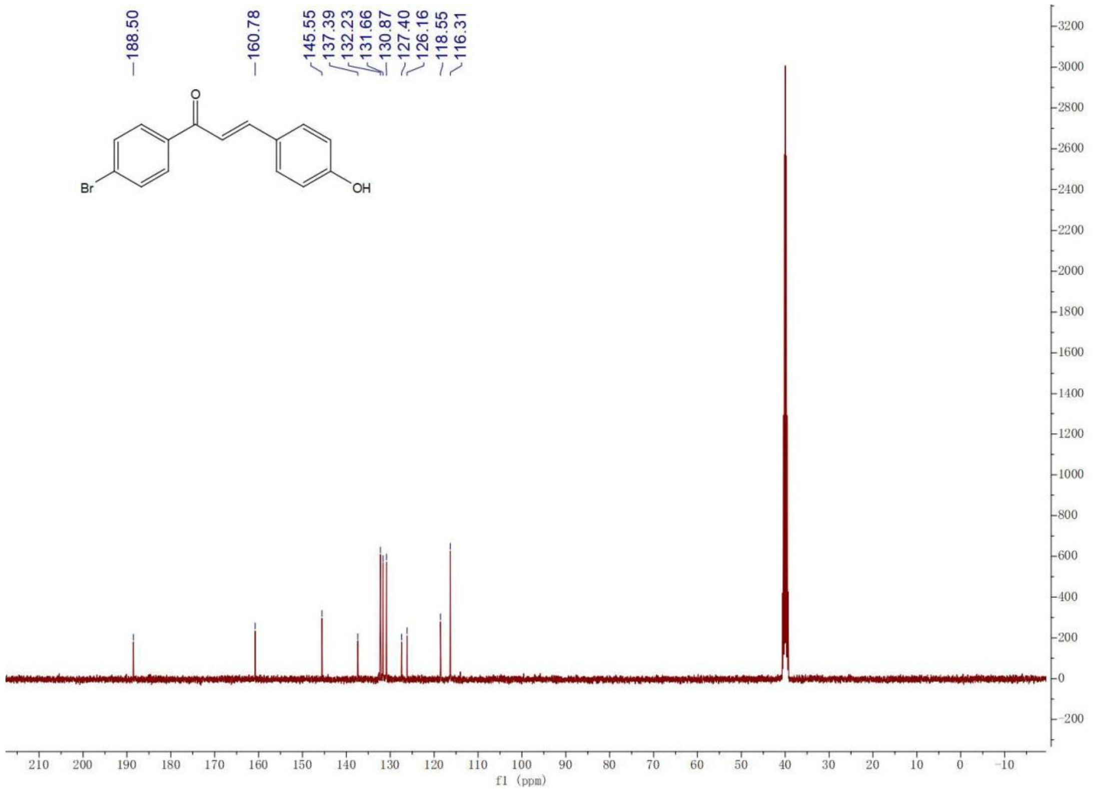

^{13}C NMR spectrum

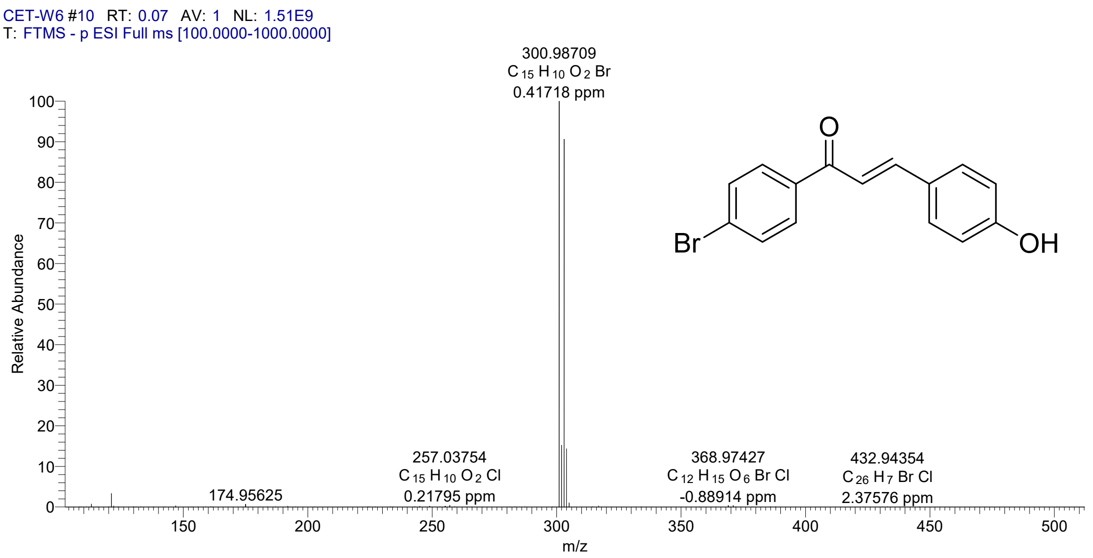

HRMS (ESI) spectrum

The C=O stretching vibration peak at 1645 cm^{-1} and the vibration peaks of O–H groups at 3260 cm^{-1}. Peaks at 1609, 1588, 1571, and 1552 cm^{-1} could be assigned to the stretching vibration of CH_2 in the aromatic nucleus. The peak at 811 cm^{-1} results from the out-of-plane bending vibration of C–H. The peaks at 1339 and 1300 cm^{-1} are ascribed to the stretching vibration of the C-C benzenoid ring. The peak at 739 cm^{-1} is assigned to the stretching vibration of the C-Br. These observations confirmed that the structure is (E)-1-(4-bromophenyl)-3-(4-hydroxyphenyl)propyl-2-en-1-one (**W6**).

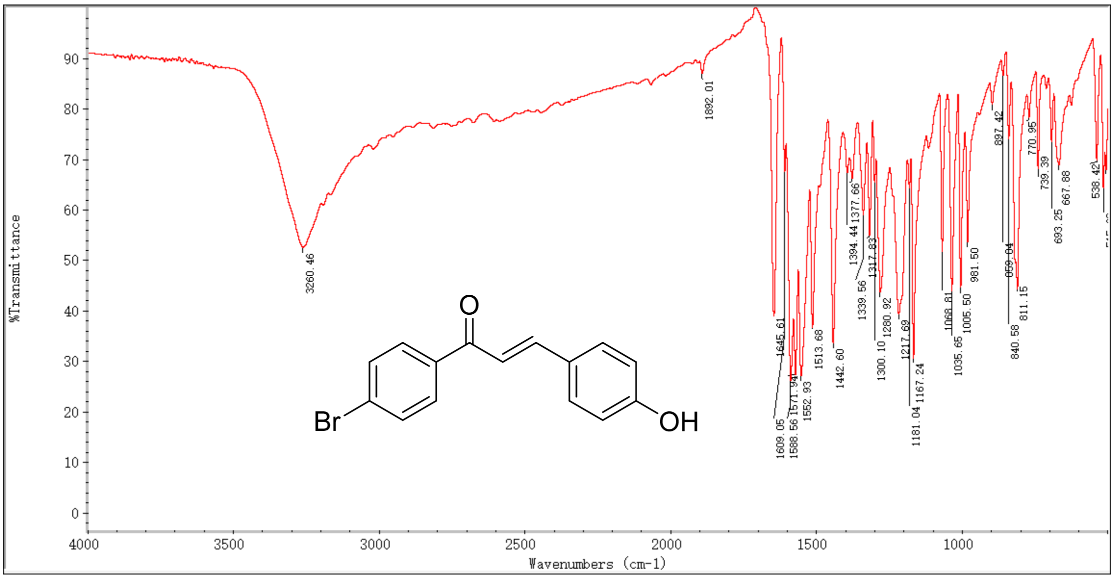

IR spectrum

(E)-1-(4-chlorophenyl)-3-(4-hydroxyphenyl)propyl-2-en-1-one (W7)

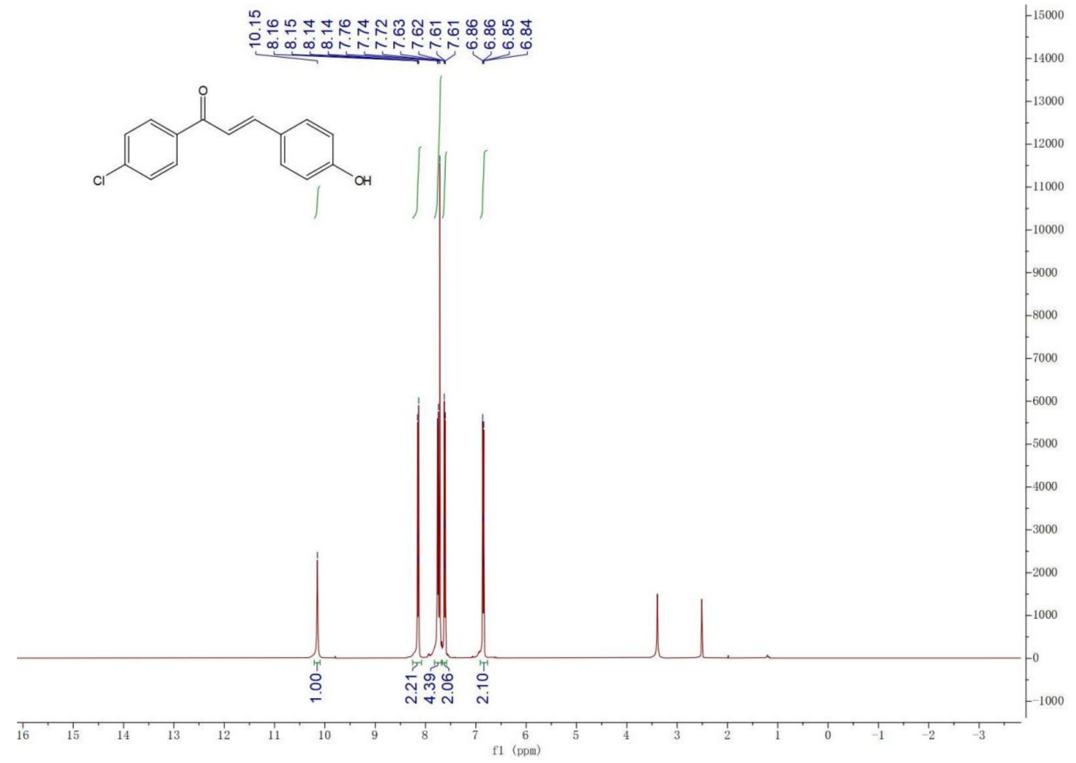

^{1}H NMR spectrum

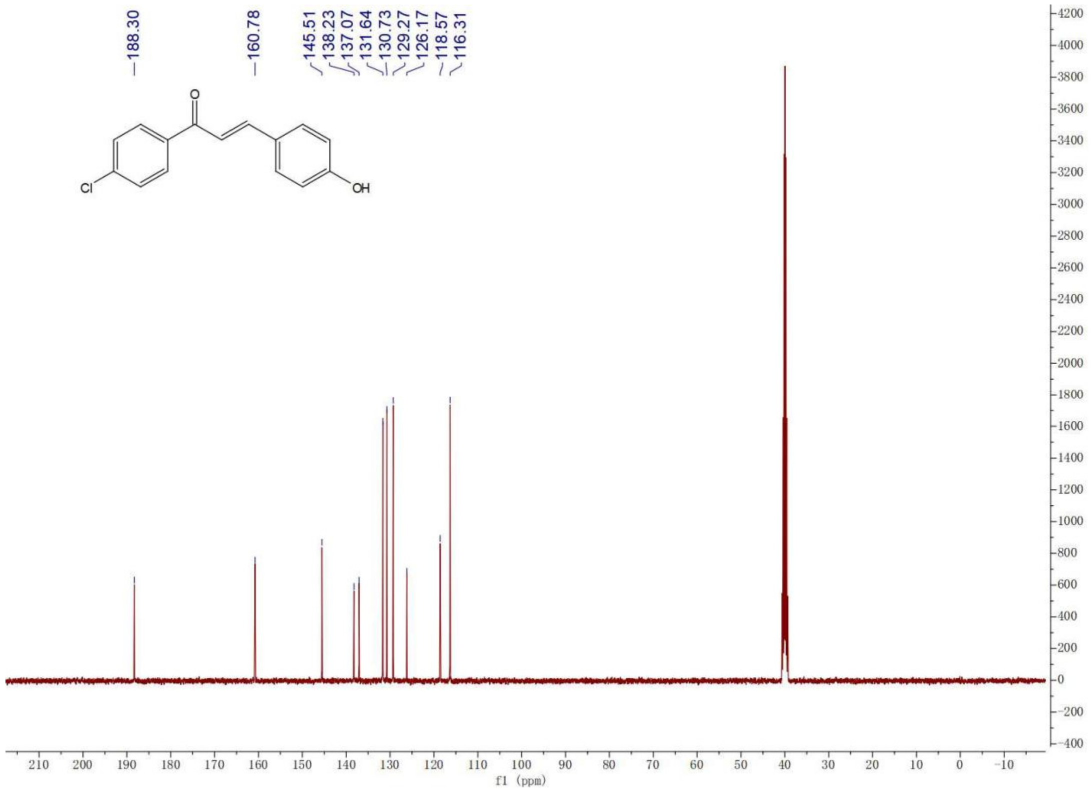

^{13}C NMR spectrum

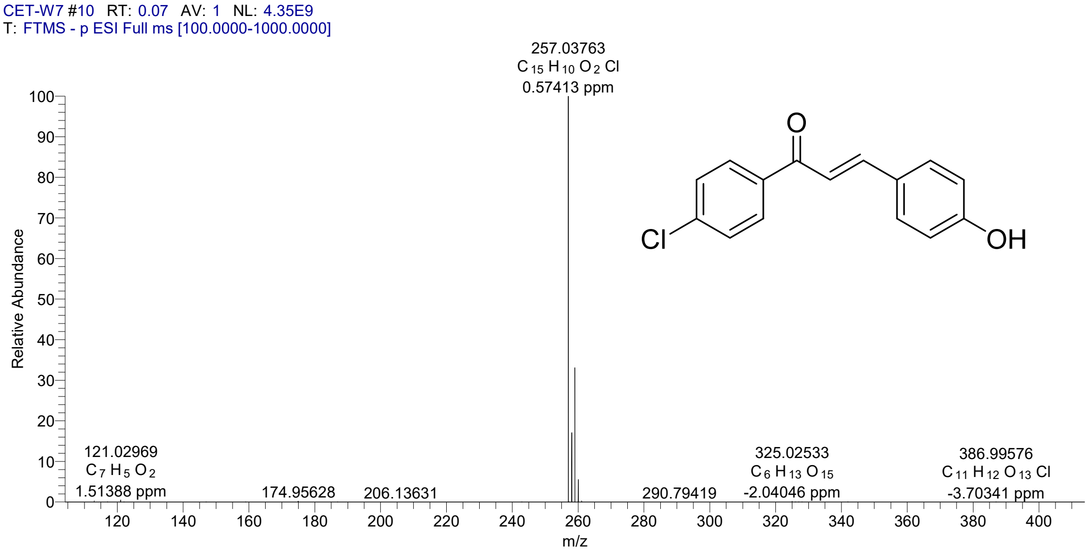

HRMS (ESI) spectrum

The C=O stretching vibration peak at 1651 cm-1 and the vibration peaks of O–H groups at 3350 cm-1. Peaks at 1594, 1575, 1560, and 1513 cm-1 could be assigned to the stretching vibration of CH_2 in the aromatic nucleus. The peak at 816 cm-1 results from the out-of-plane bending vibration of C–H. The peaks at 1397 and 1362 cm-1 are ascribed to the stretching vibration of the C-C benzenoid ring. The peak at 627 cm-1 is assigned to the stretching vibration of the C-Cl. These observations confirmed that the structure is (E)-1-(4-chlorophenyl)-3-(4-hydroxyphenyl)propyl-2-en-1-one (**W7**).

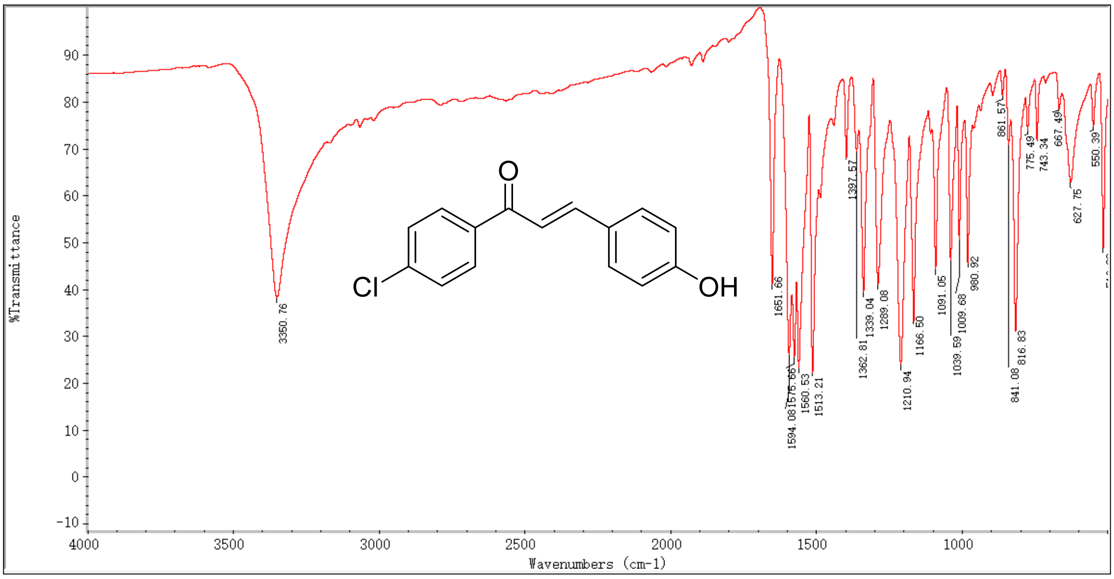

IR spectrum

(E)-1-(2,4-dimethylphenyl)-3-(4-hydroxyphenyl)propyl-2-en-1-one (W8)

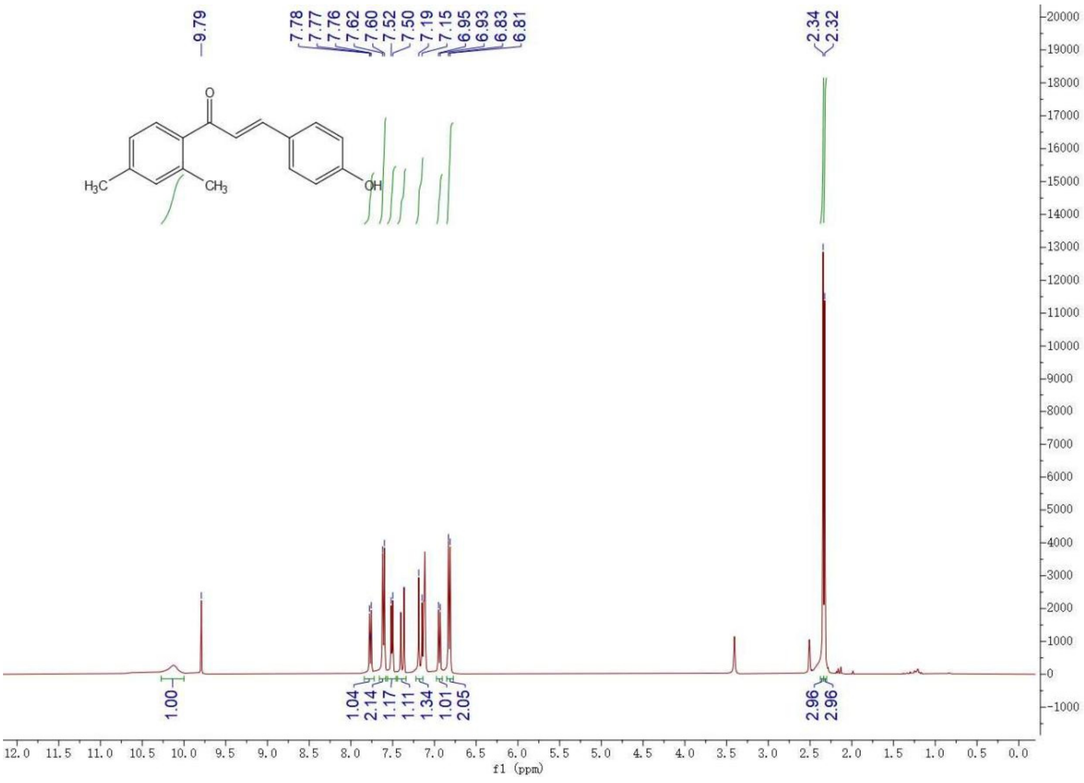

^{1}H NMR spectrum

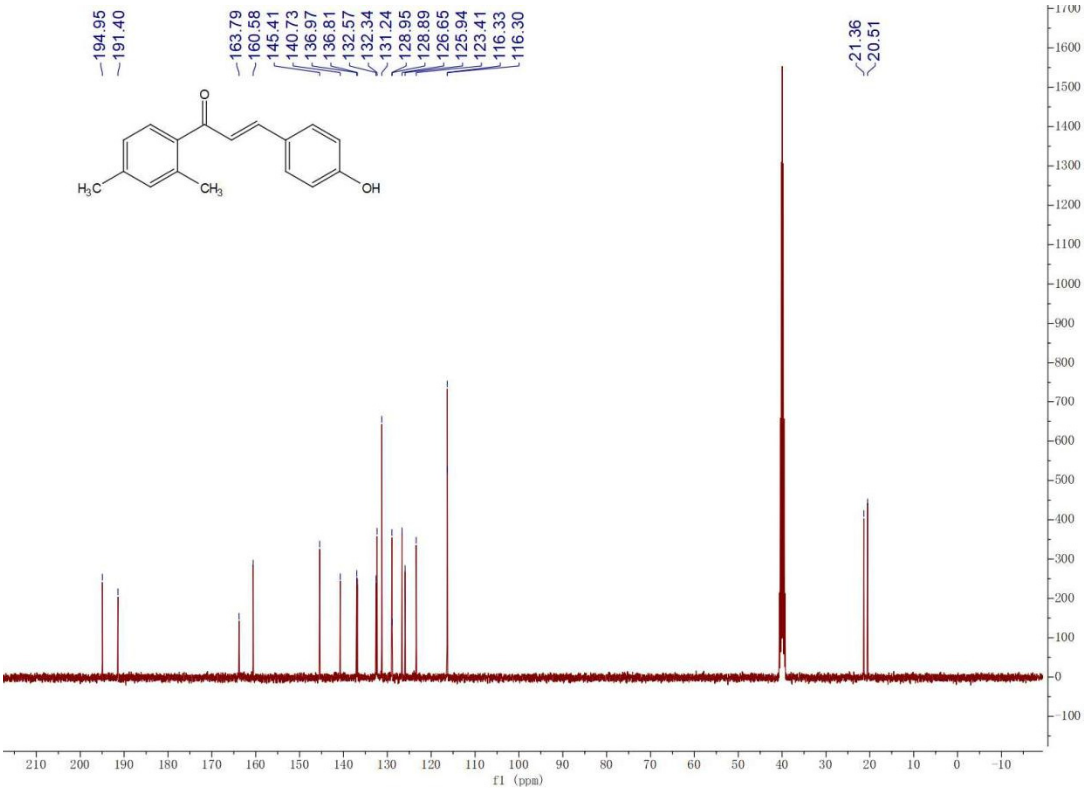

^{13}C NMR spectrum

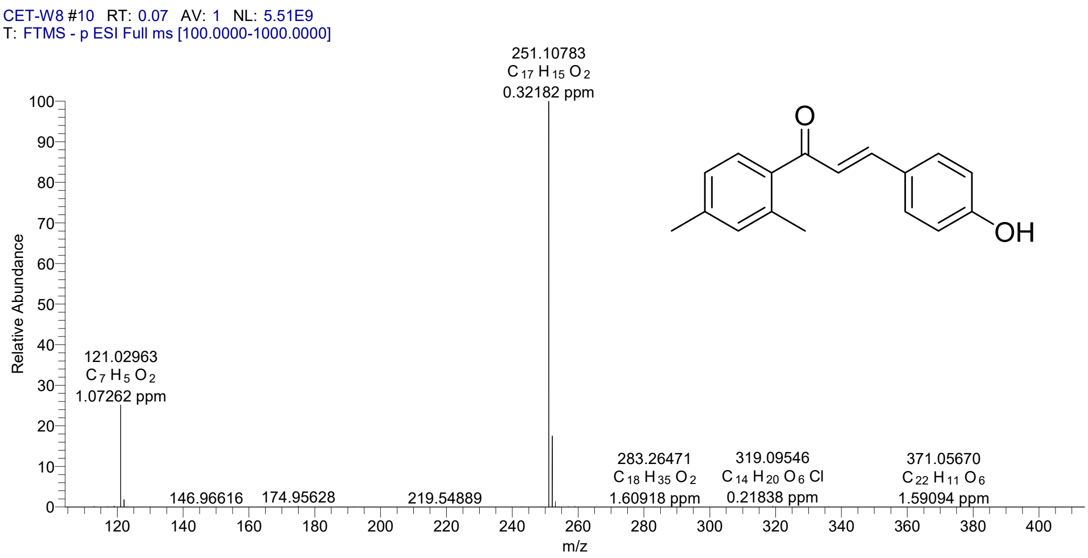

HRMS (ESI) spectrum

The C=O stretching vibration peak at 1666 cm^{-1} and the vibration peaks of O–H groups at 3161 cm^{-1}. The peaks at 3019 and 2963 cm^{-1} could be attributed to the stretching vibration of C–H in CH_3. Peaks at 1572, 1513, and 1444 cm^{-1} could be assigned to the stretching vibration of CH_2 in the aromatic nucleus. The peak at 818 cm^{-1} results from the out-of-plane bending vibration of C–H. The peaks at 1383 and 1335 cm^{-1} are ascribed to the stretching vibration of the C-C benzenoid ring. The peaks at 1025 and 980 cm^{-1} are assigned to C–H of the methyl group. These observations confirmed that the structure is (E)-1-(2,4-dimethylphenyl)-3-(4-hydroxyphenyl)propyl-2-en-1-one (**W8**).

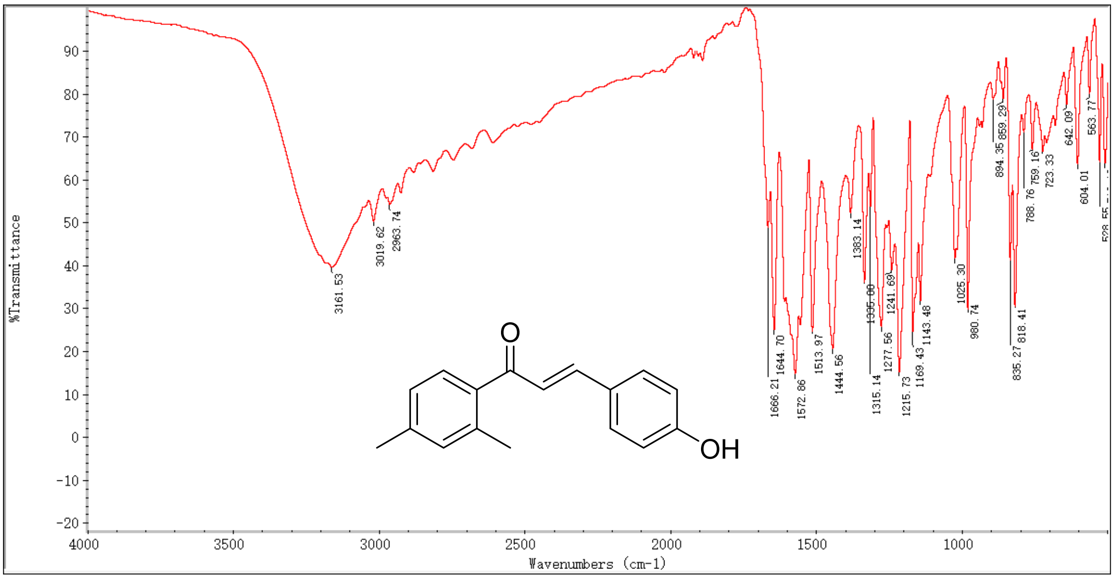

IR spectrum

(E)-1-(3,4-dimethylphenyl)-3-(4-hydroxyphenyl)propyl-2-en-1-one (W9)

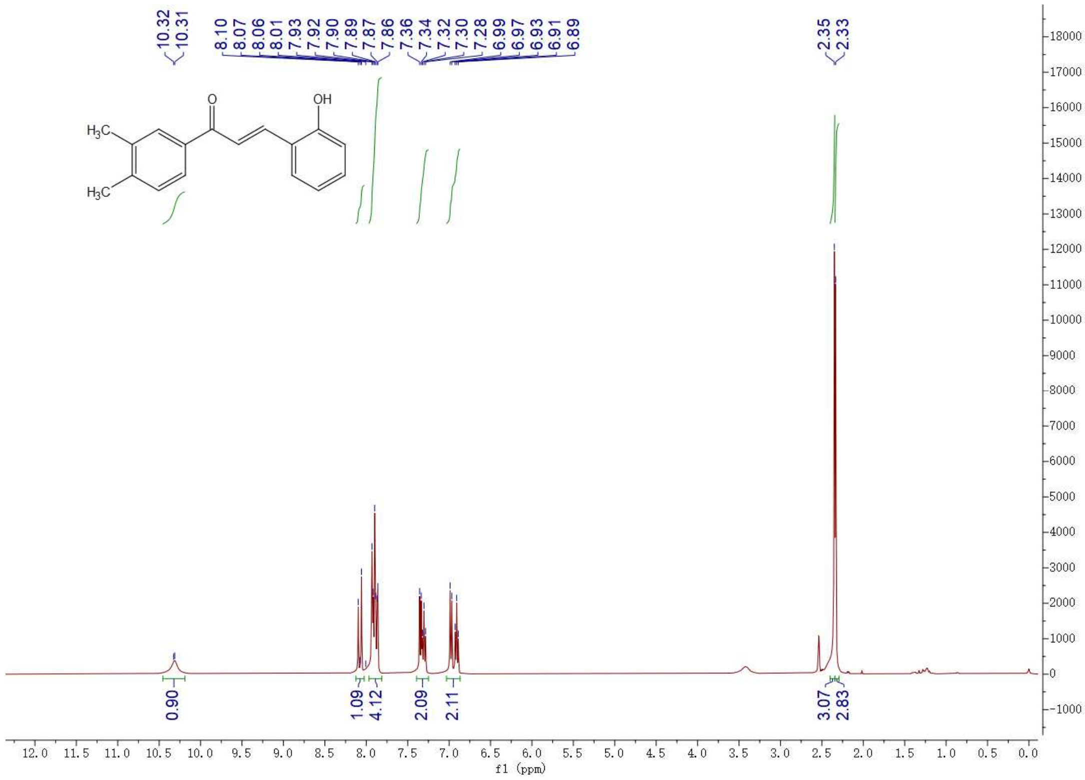

^{1}H NMR spectrum

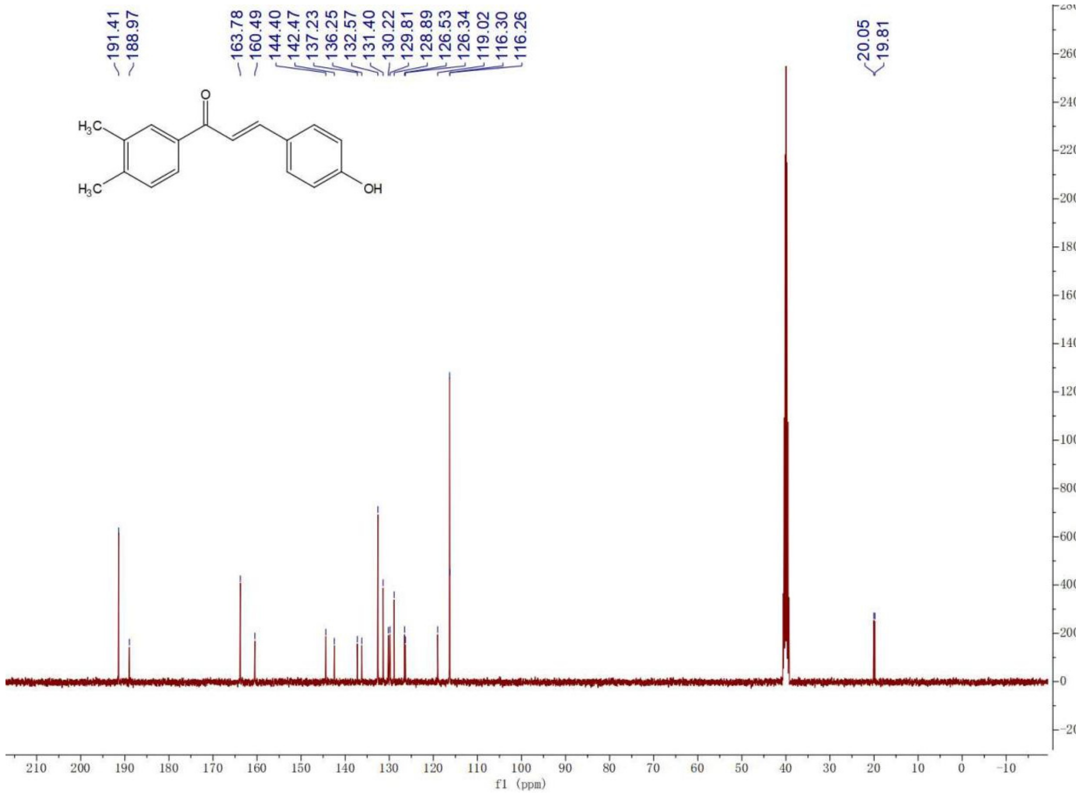

^{13}C NMR spectrum

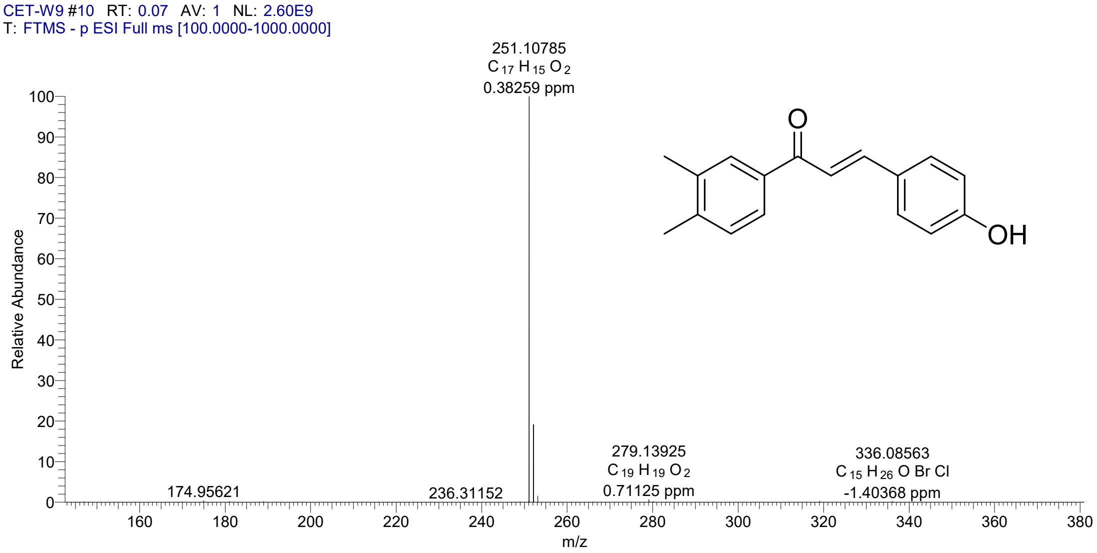

HRMS (ESI) spectrum

The C=O stretching vibration peak at 1666 cm-1 and the vibration peaks of O–H groups at 3169 cm-1. The peak at 2878 cm-1 could be attributed to the stretching vibration of C–H in CH_3. Peaks at 1599, 1573, and 1555 cm-1 could be assigned to the stretching vibration of CH_2 in the aromatic nucleus. The peak at 834 cm−1 results from the out-of-plane bending vibration of C–H. The peaks at 1350 and 1315 cm−1 are ascribed to the stretching vibration of the C-C benzenoid ring. The peaks at 1047 and 979 cm−1 are assigned to C–H of the methyl group. These observations confirmed that the structure is (E)-1-(3,4-dimethylphenyl)-3-(4-hydroxyphenyl)propyl-2-en-1-one (**W9**).

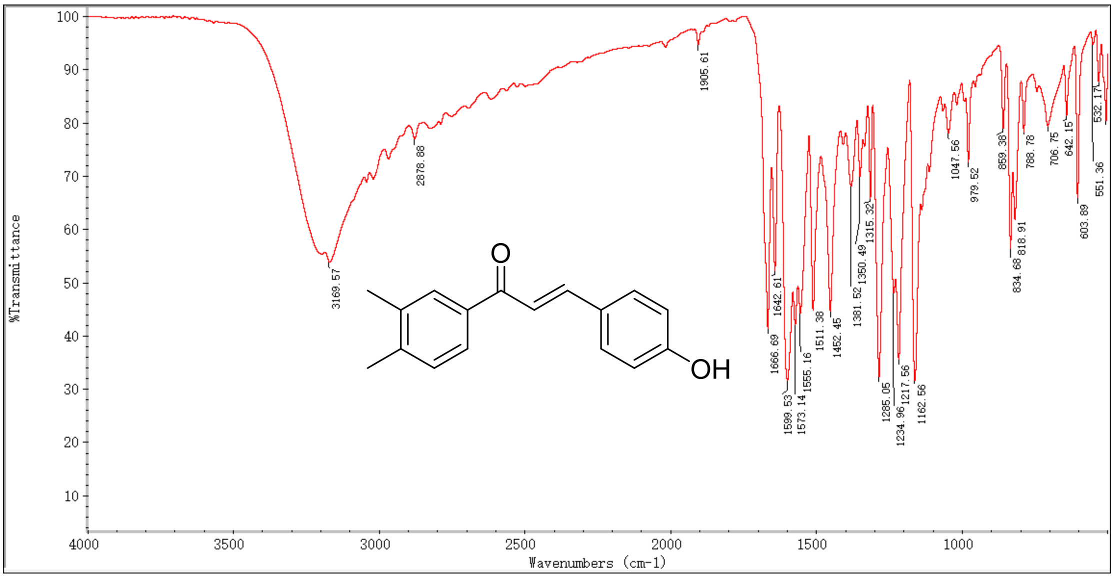

IR spectrum

(E)-1-(3-fluorophenyl)-3-(4-hydroxyphenyl)propyl-2-en-1-one (W10)

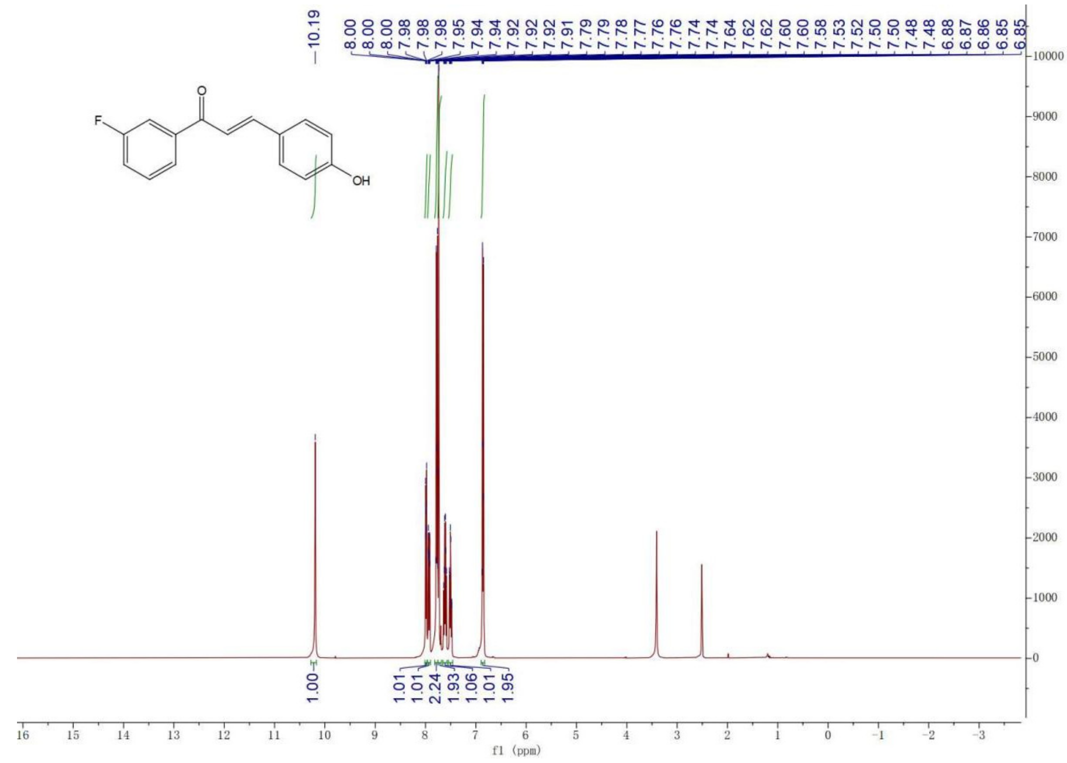

^{1}H NMR spectrum

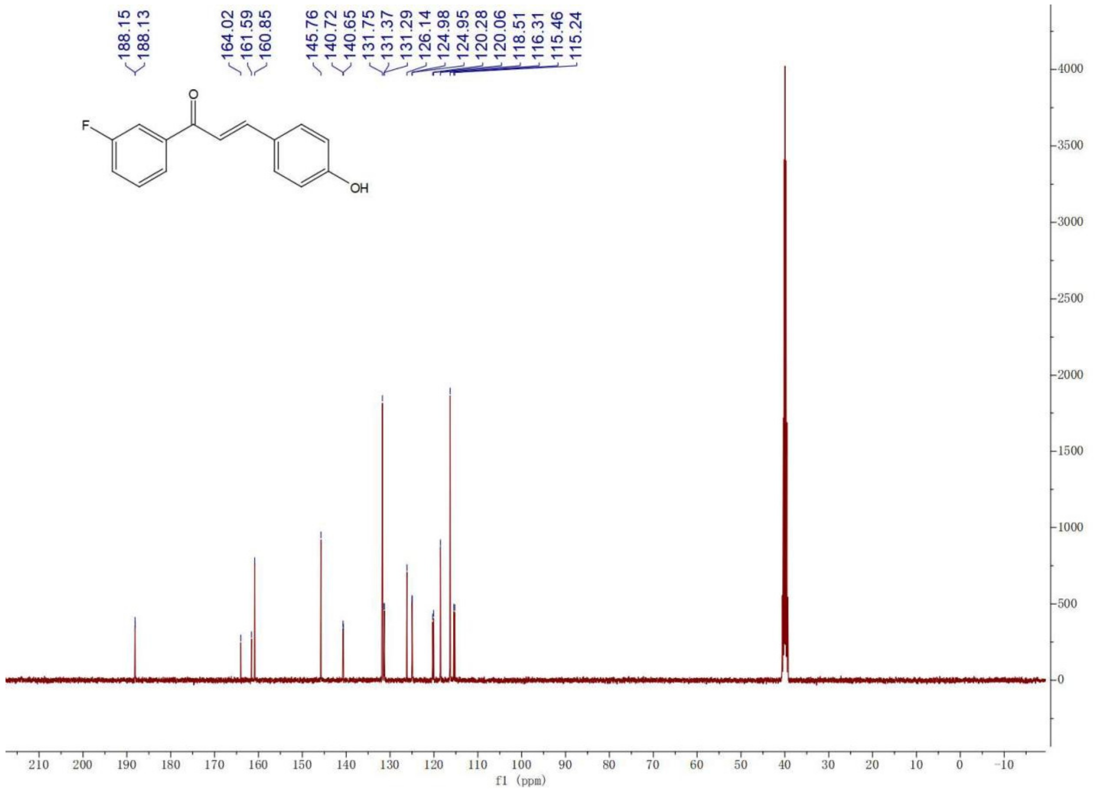

^{13}C NMR spectrum

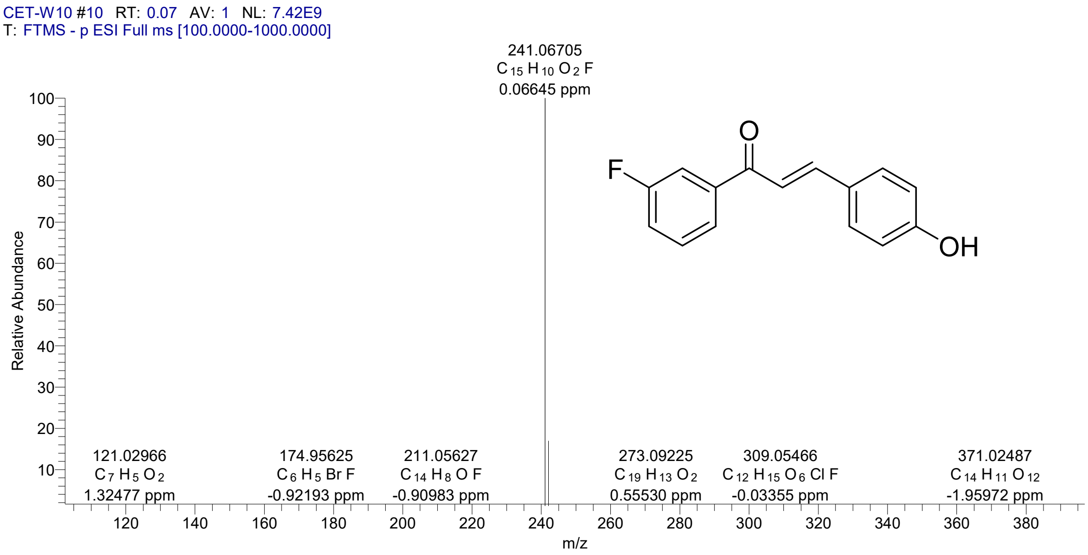

HRMS (ESI) spectrum

The C=O stretching vibration peak at 1649 cm-1 and the vibration peaks of O–H groups at 3242 cm-1. Peaks at 1608, 1559, 1512, and 1441 cm-1 could be assigned to the stretching vibration of CH_2 in the aromatic nucleus. The peak at 837 cm−1 results from the out-of-plane bending vibration of C–H. The peaks at 1336 and 1301 cm−1 are ascribed to the stretching vibration of the C-C benzenoid ring. The peak at 740 cm−1 is assigned to the stretching vibration of the C-Br. These observations confirmed that the structure is (E)-1-(3-fluorophenyl)-3-(4-hydroxyphenyl)propyl-2-en-1-one (**W10**).

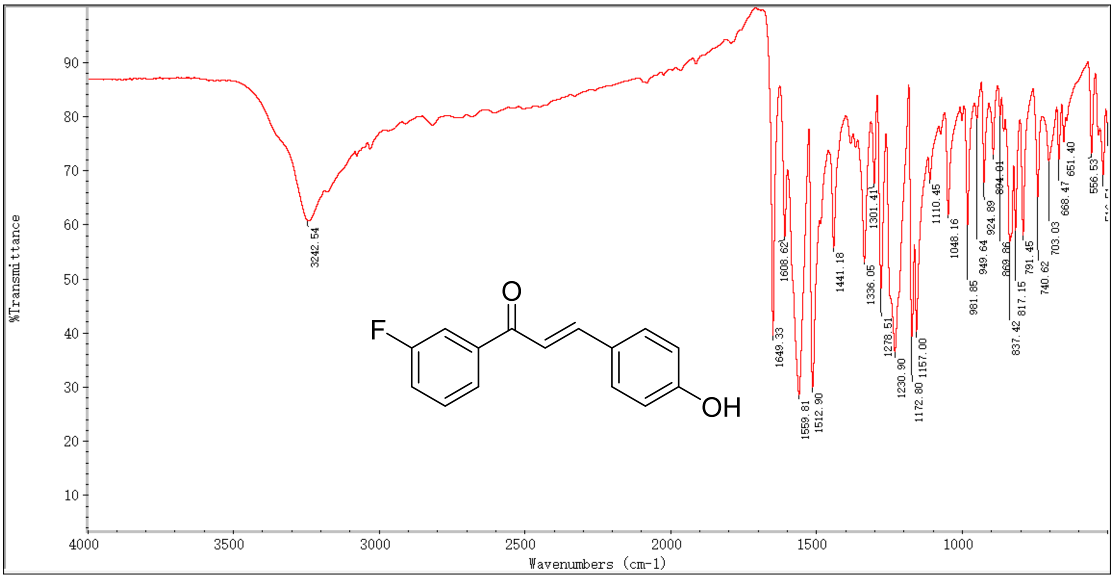

IR spectrum

(E)-3-(4-hydroxyphenyl)-1-(naphthalene-1-yl)propyl-2-en-1-one (W11)

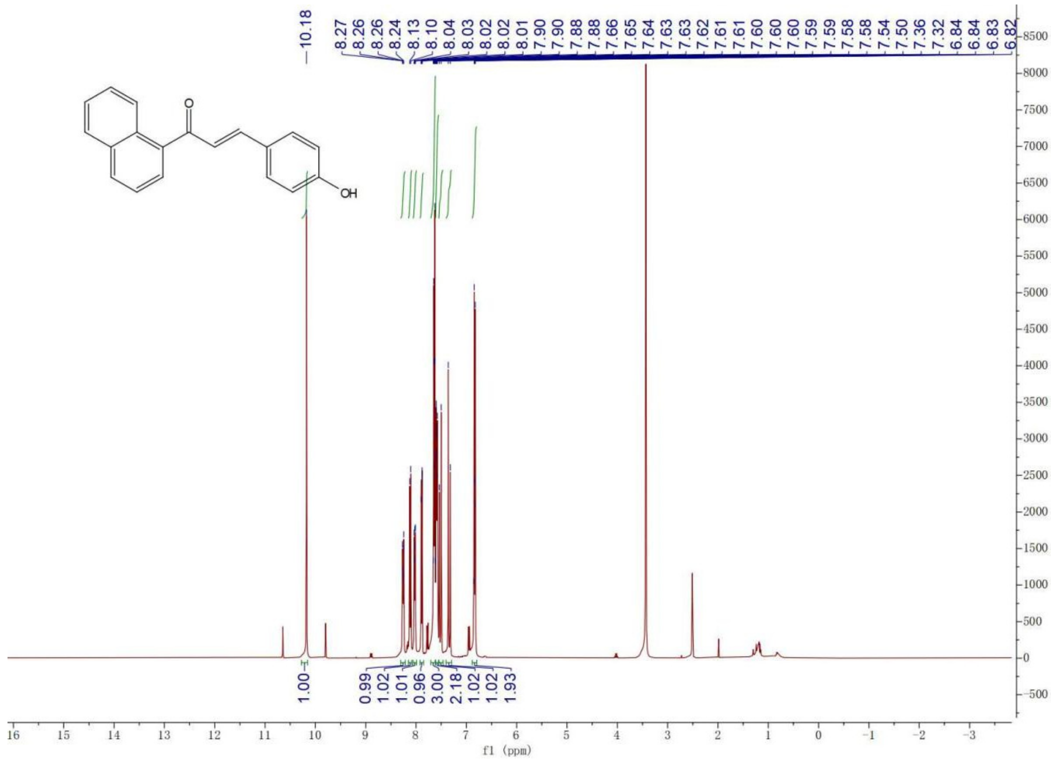

^{1}H NMR spectrum

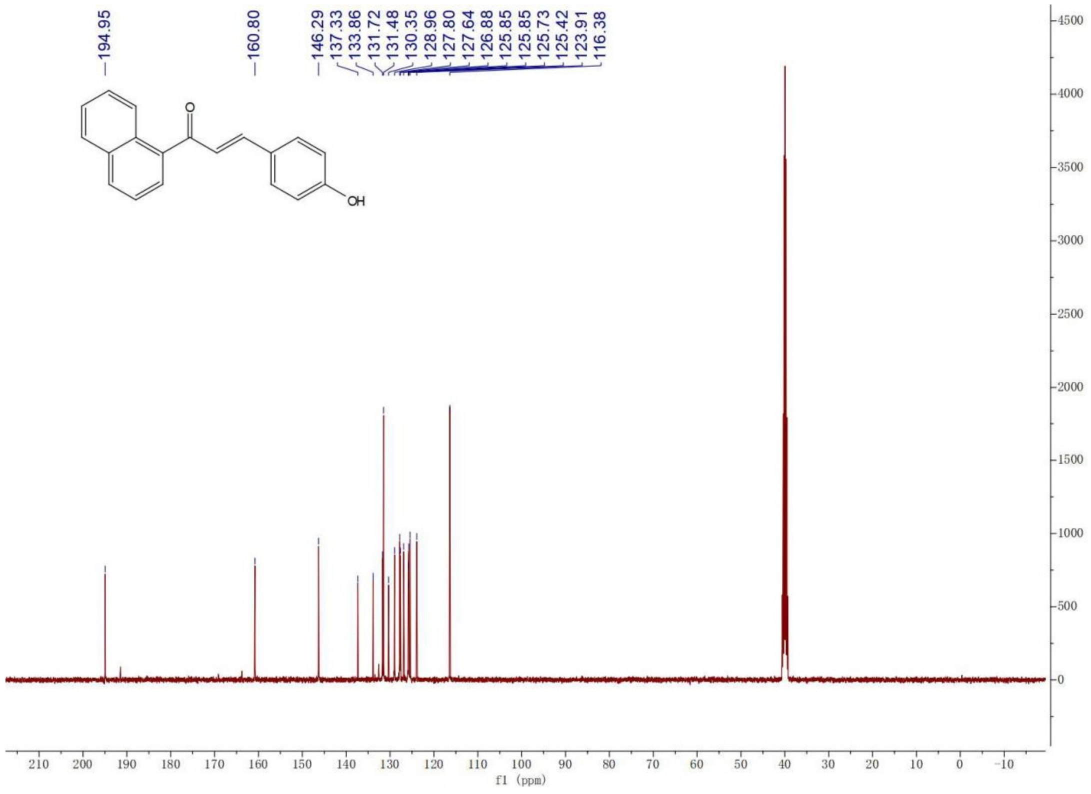

^{13}C NMR spectrum

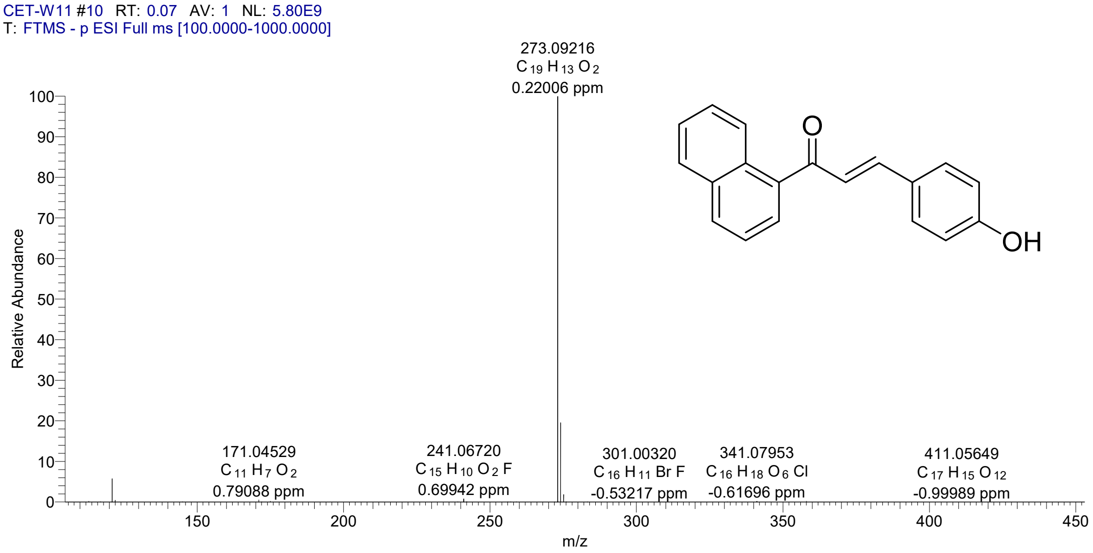

HRMS (ESI) spectrum

The C=O stretching vibration peak at 1649 cm⁻¹ and the vibration peaks of O–H groups at 3370 cm⁻¹. Peaks at 1592, 1566, 1511, and 1432 cm⁻¹ could be assigned to the stretching vibration of CH_2 in the aromatic nucleus. The peak at 831 cm⁻¹ results from the out-of-plane bending vibration of C–H. The peaks at 1362 and 1326 cm⁻¹ are ascribed to the stretching vibration of the C-C benzenoid ring. These observations confirmed that the structure is (E)-3-(4-hydroxyphenyl)-1-(naphthalene-1-yl)propyl-2-en-1-one (**W11**).

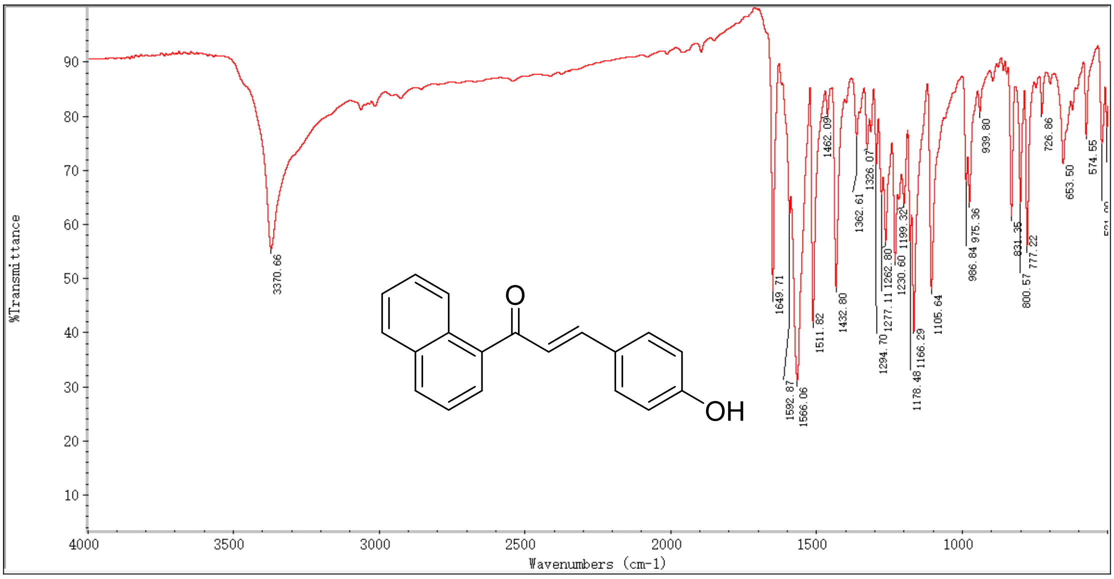

IR spectrum

(E)-3-(4-hydroxyphenyl)-1-phenylpropyl-2-en-1-one (F1)

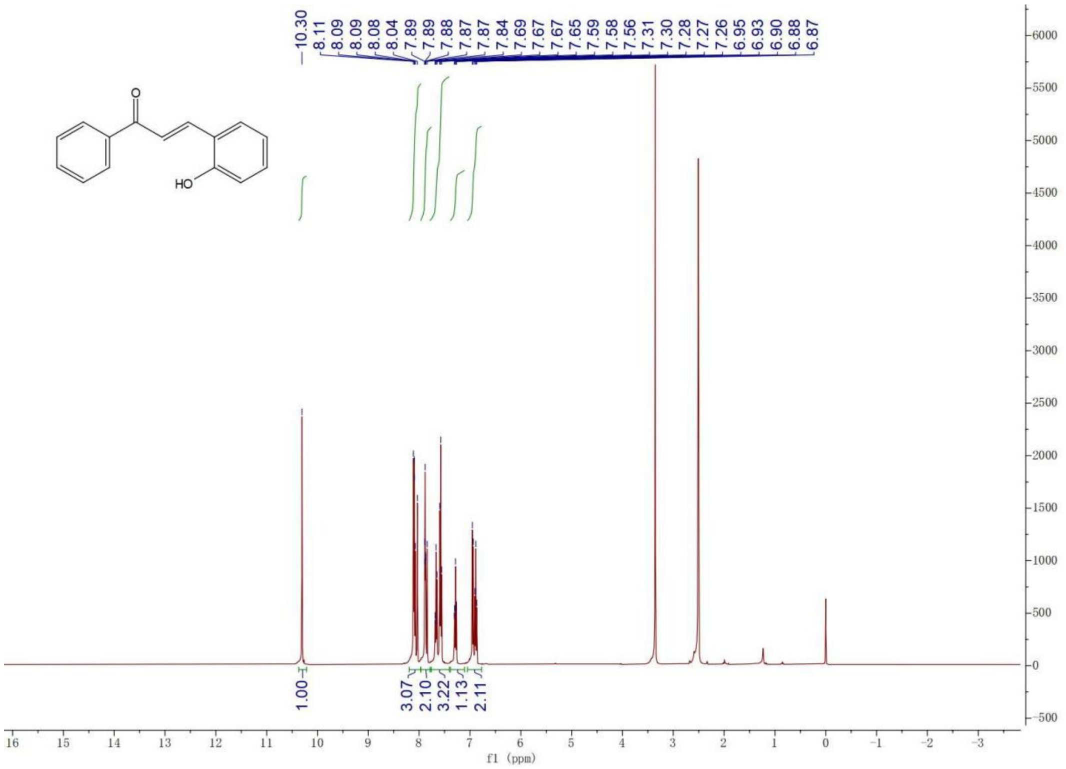

^{1}H NMR spectrum

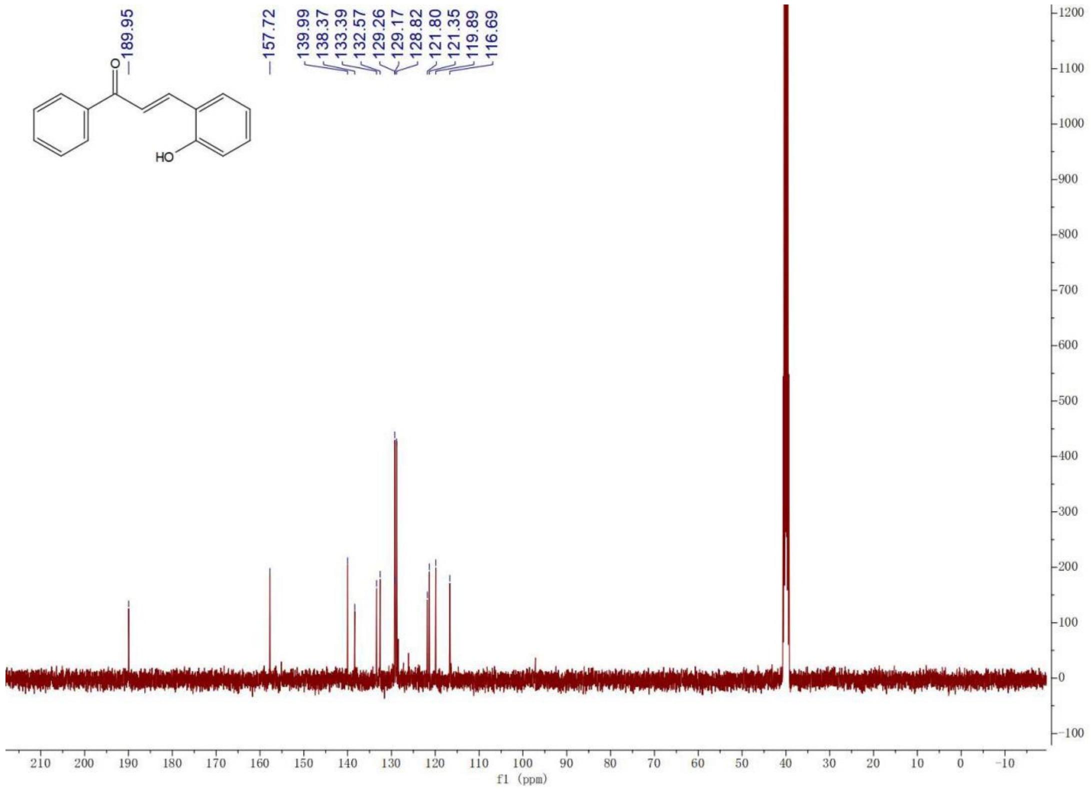

^{13}C NMR spectrum

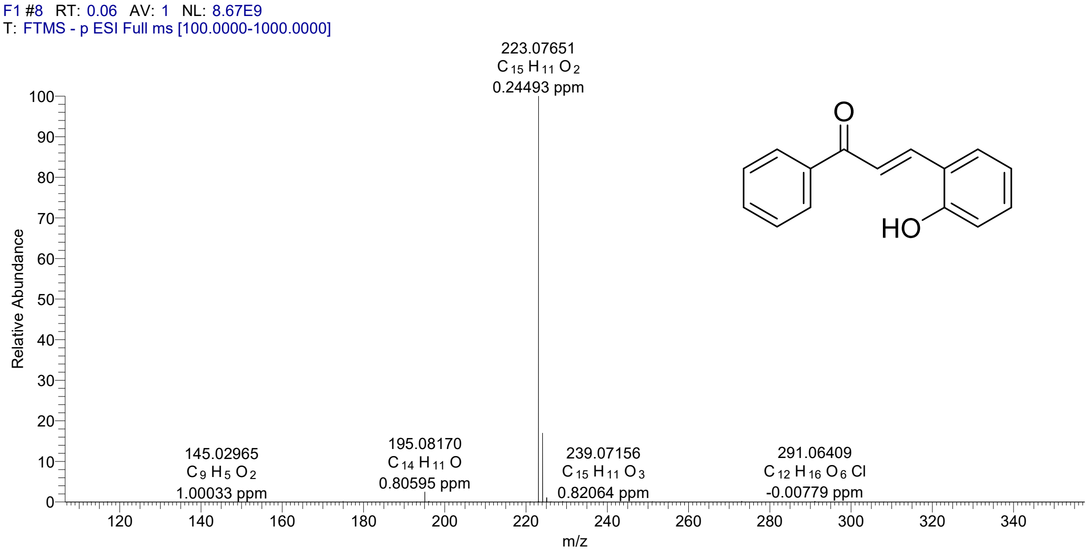

HRMS (ESI) spectrum

The C=O stretching vibration peak at 1651 cm^{-1} and the vibration peaks of O–H groups at 3365 cm^{-1}. Peaks at 1599, 1585, 1567, and 1458 cm^{-1} could be assigned to the stretching vibration of CH_2 in the aromatic nucleus. The peak at 755 cm^{-1} results from the out-of-plane bending vibration of C–H. The peaks at 1344 and 1317 cm^{-1} are ascribed to the stretching vibration of the C-C benzenoid ring. These observations confirmed that the structure is €-3-(4-hydroxyphenyl)-1-phenylpropyl-2-en-1-one (**F1**).

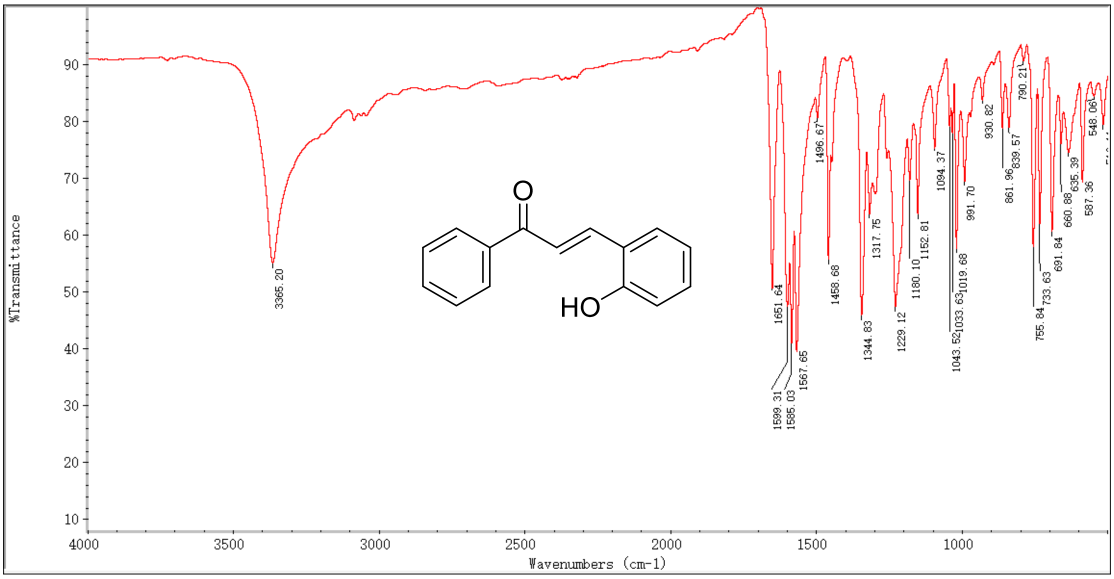

IR spectrum

(E)-3-(2-hydroxyphenyl)-1-(3-methoxyphenyl)propyl-2-en-1-one (F2)

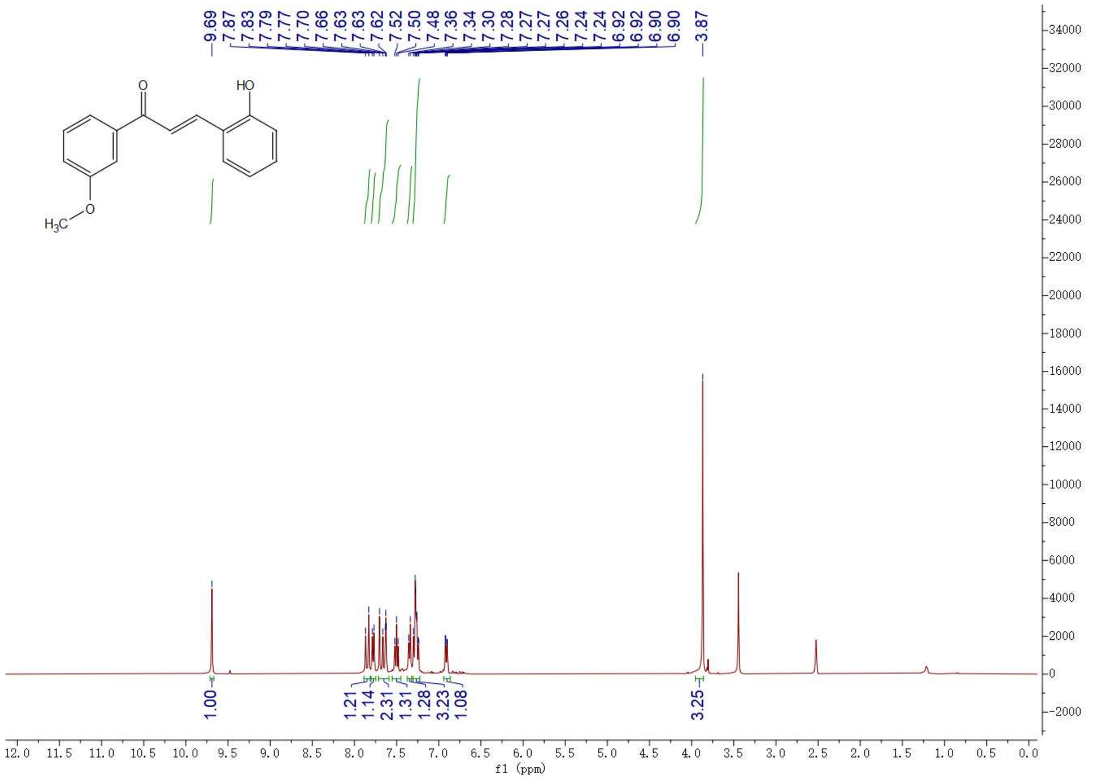

^{1}H NMR spectrum

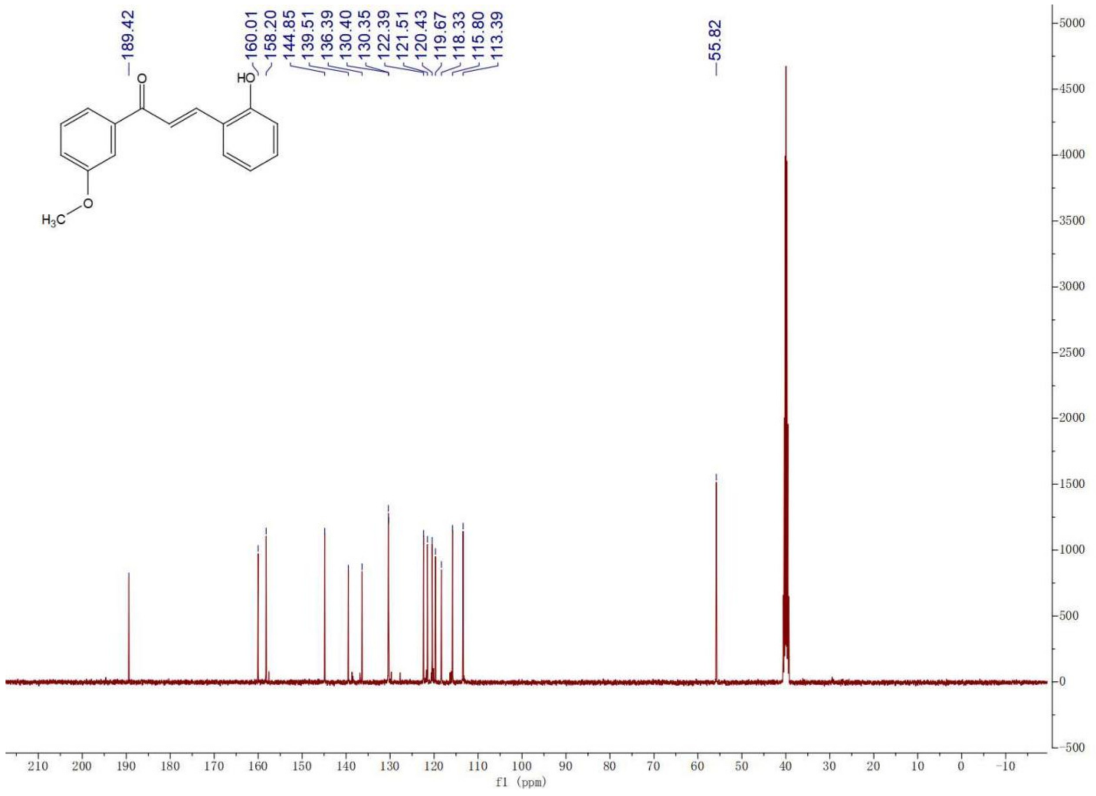

^{13}C NMR spectrum

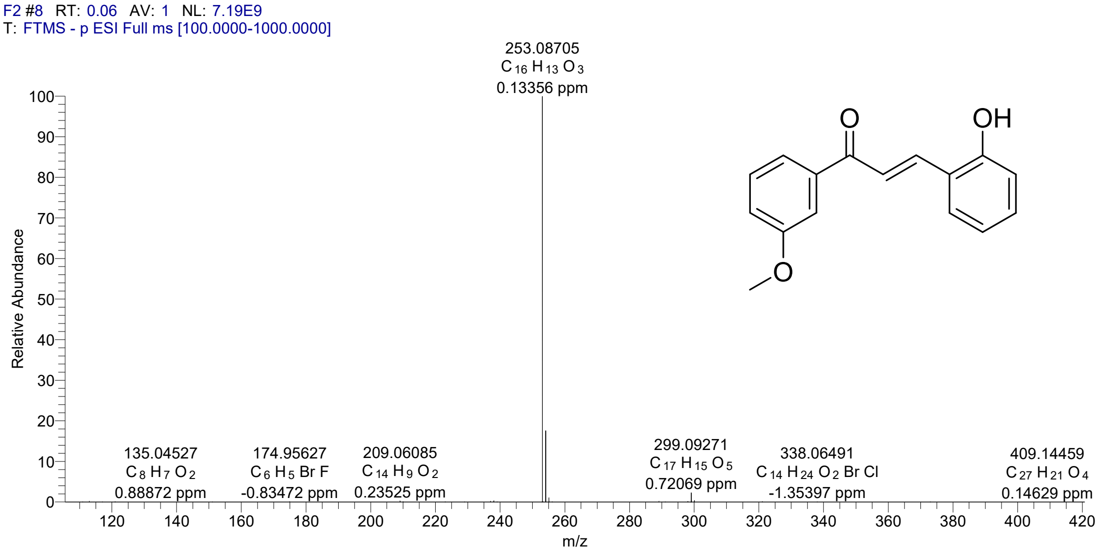

HRMS (ESI) spectrum

The C=O stretching vibration peak at 1653 cm^{-1} and the vibration peaks of O–H groups at 3356 cm^{-1}. The peaks at 2961 and 2937 cm^{-1} could be attributed to $–OCH_3$. Peaks at 1580, 1487, 1464, and 1447 cm^{-1} could be assigned to the stretching vibration of CH_2 in the aromatic nucleus. The peak at 843 cm^{-1} results from the out-of-plane bending vibration of C–H. The peaks at 1368 and 1345 cm^{-1} are ascribed to the stretching vibration of the C-C benzenoid ring. The peaks at 1058, 1034, and 995 cm^{-1} are assigned to C–H of the methoxy group. These observations confirmed that the structure is (E)-3-(2-hydroxyphenyl)-1-(3-methoxyphenyl)propyl-2-en-1-one (**F2**).

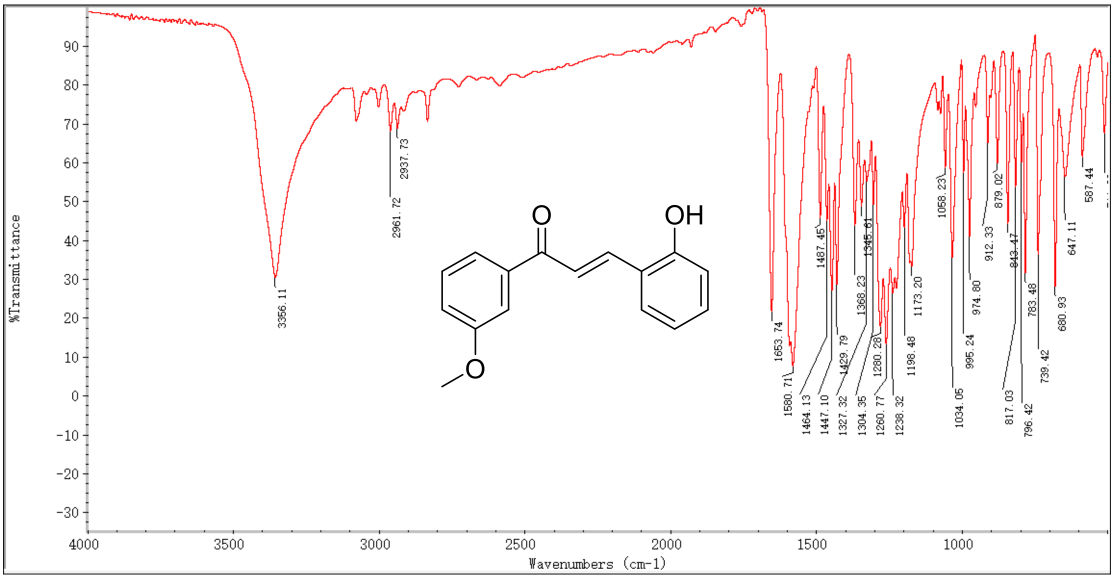

IR spectrum

(E)-3-(3-hydroxyphenyl)-1-(3-methoxyphenyl)propyl-2-en-1-one (F3)

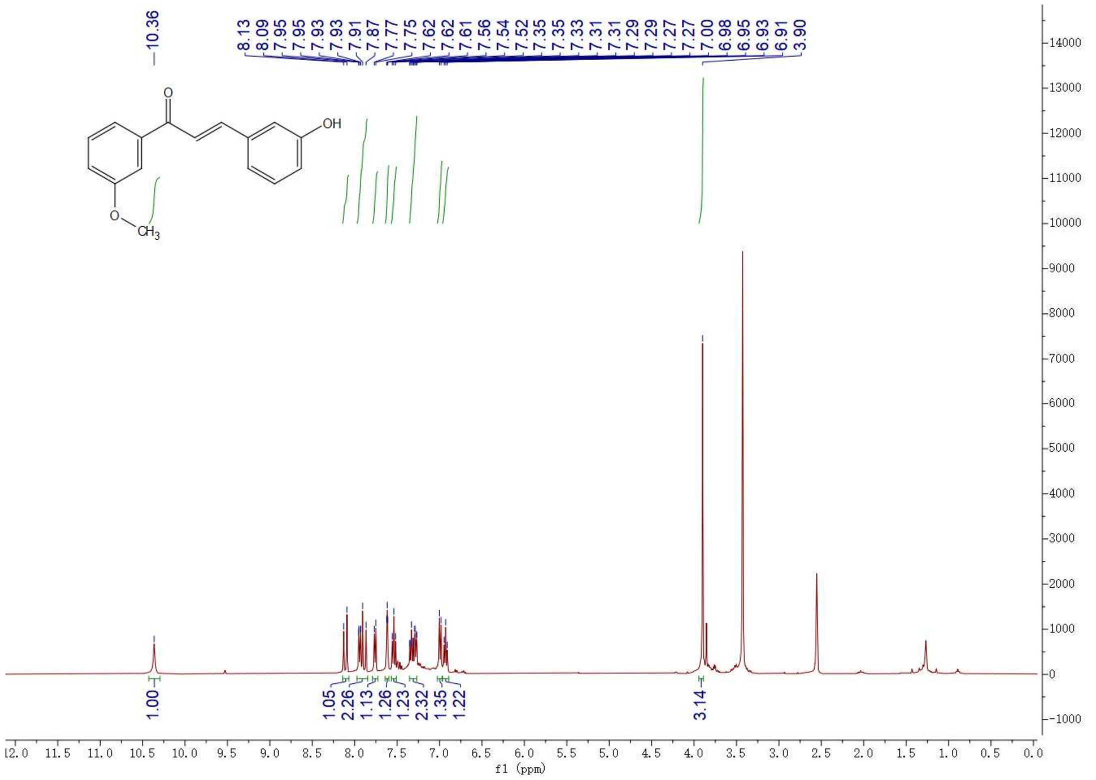

^{1}H NMR spectrum

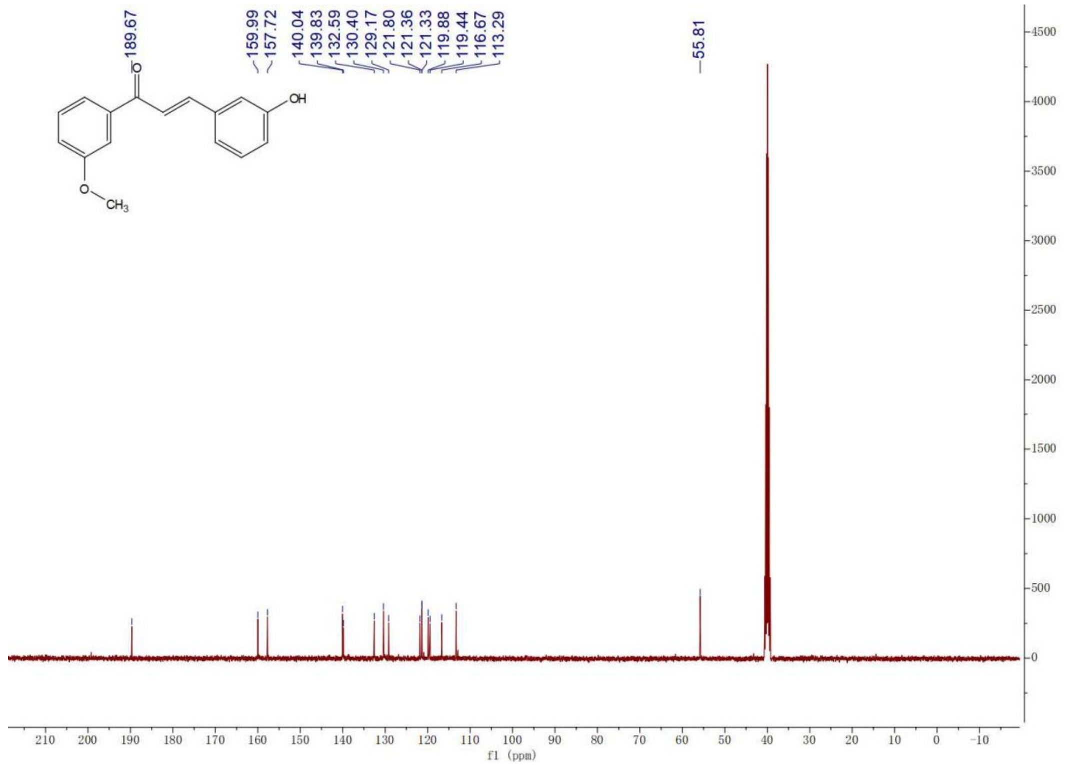

^{13}C NMR spectrum

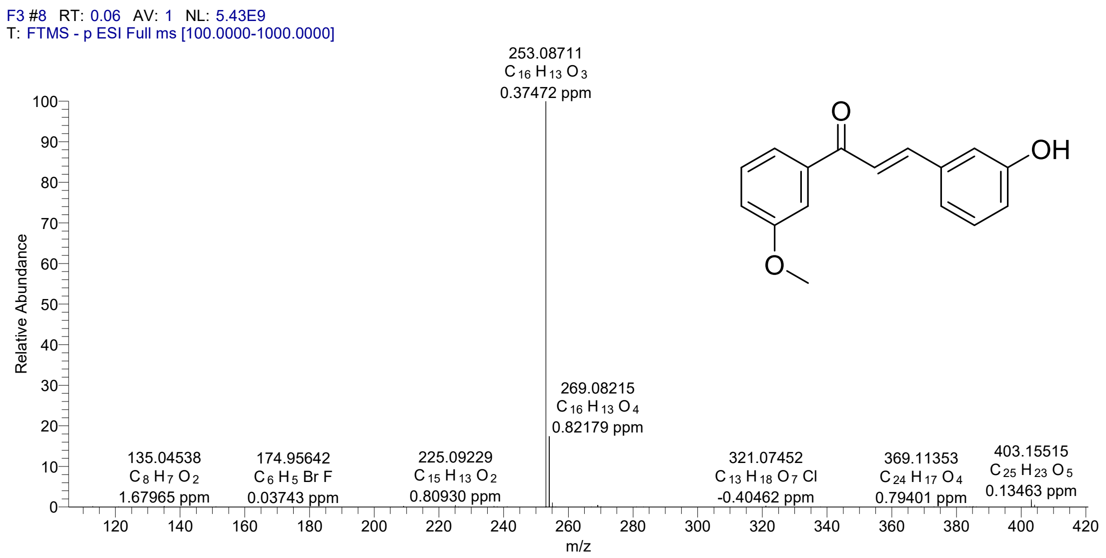

HRMS (ESI) spectrum

(E)-3-(3-hydroxyphenyl)-1-phenylpropyl-2-en-1-one (F4)

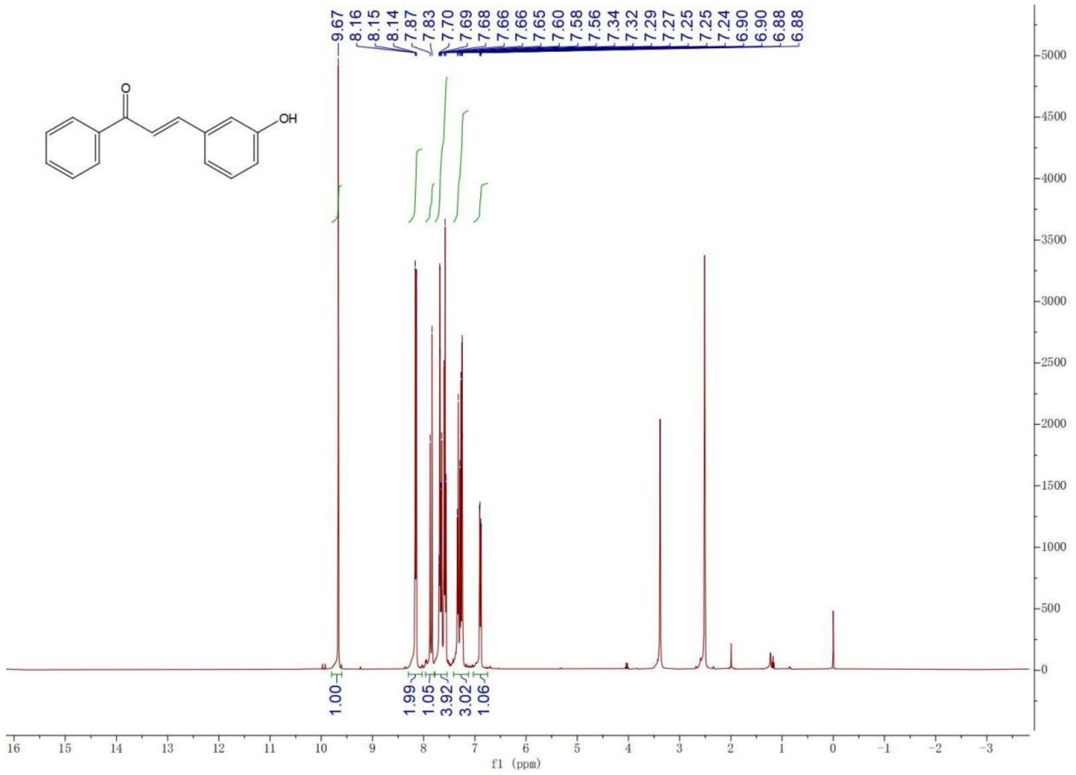

^{1}H NMR spectrum

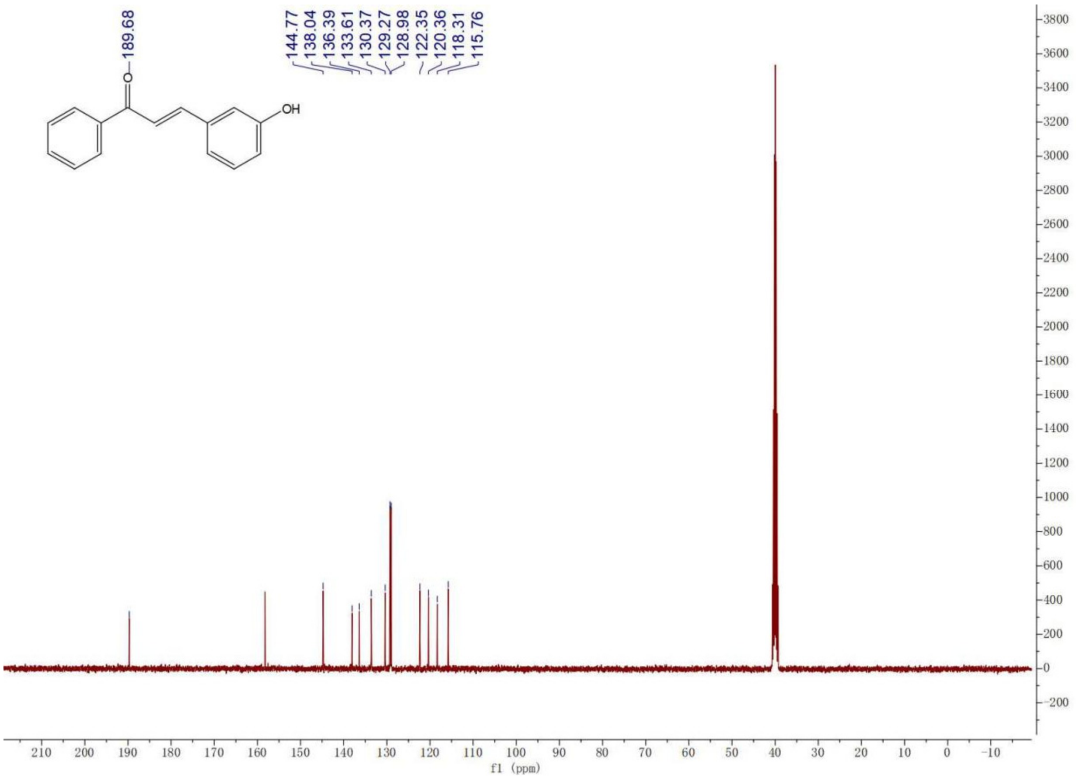

^{13}C NMR spectrum

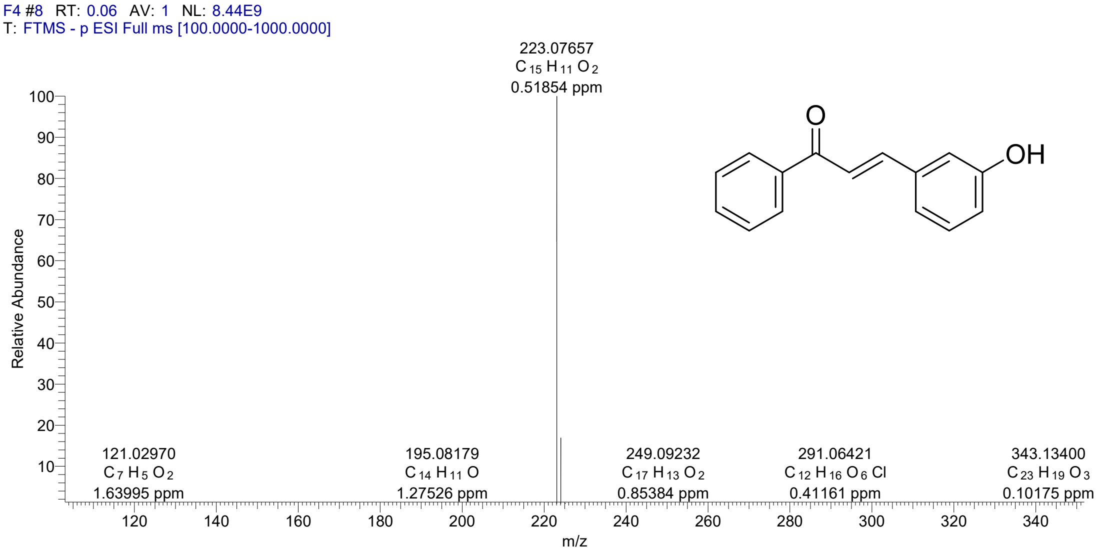

HRMS (ESI) spectrum

The C=O stretching vibration peak at 1651 cm^{-1} and the vibration peaks of O–H groups at 3329 cm^{-1}. Peaks at 1603, 1580, 1489, and 1463 cm^{-1} could be assigned to the stretching vibration of CH_2 in the aromatic nucleus. The peak at 772 cm^{-1} results from the out-of-plane bending vibration of C–H. The peaks at 1329 and 1314 cm^{-1} are ascribed to the stretching vibration of the C-C benzenoid ring. These observations confirmed that the structure is (E)-3-(3-hydroxyphenyl)-1-phenylpropyl-2-en-1-one (**F4**).

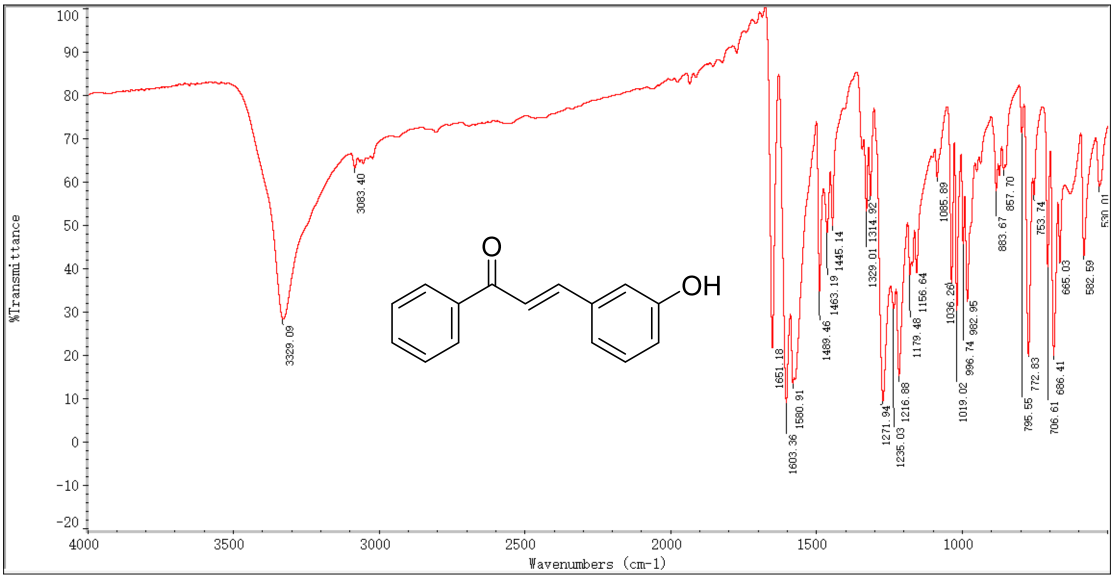

IR spectrum

(E)-3-(2-hydroxyphenyl)-1-(4-methoxyphenyl)propyl-2-en-1-one (F5)

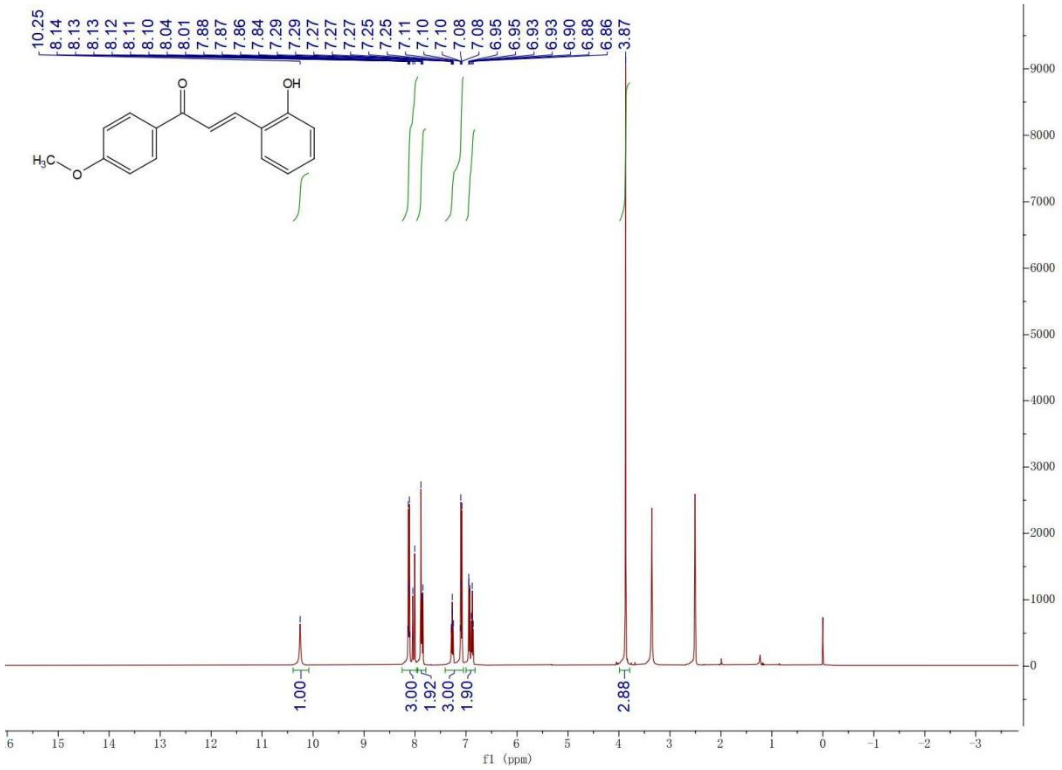

^{1}H NMR spectrum

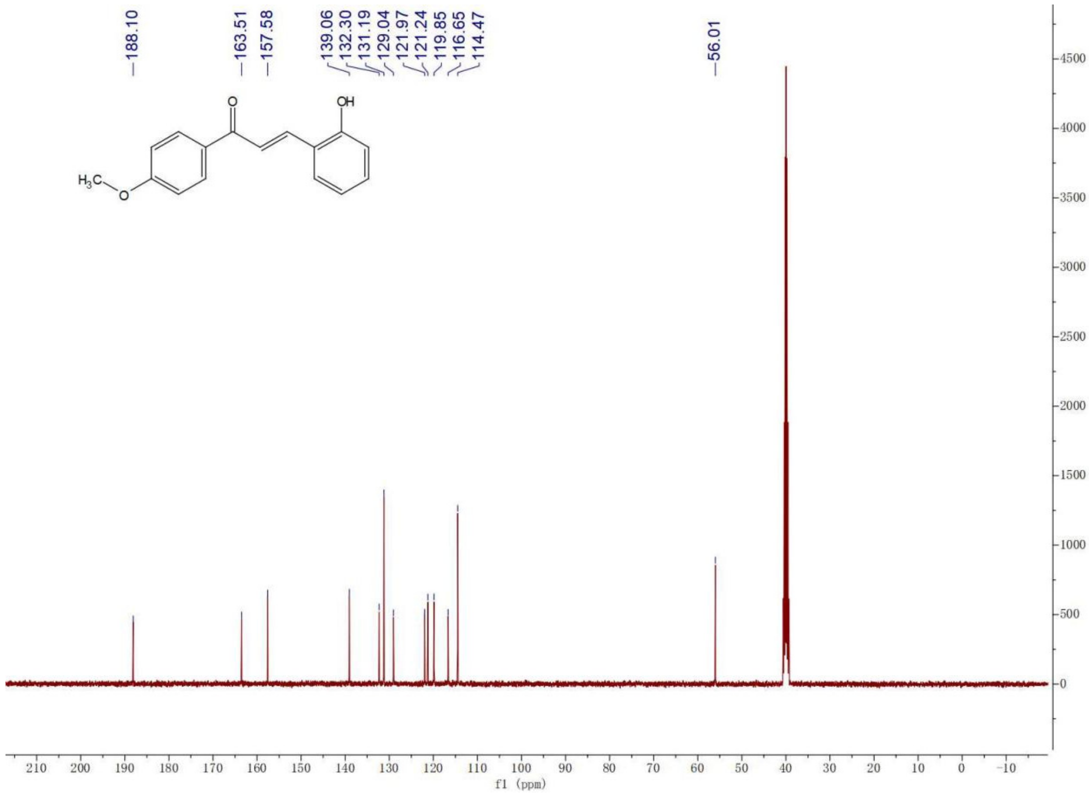

^{13}C NMR spectrum

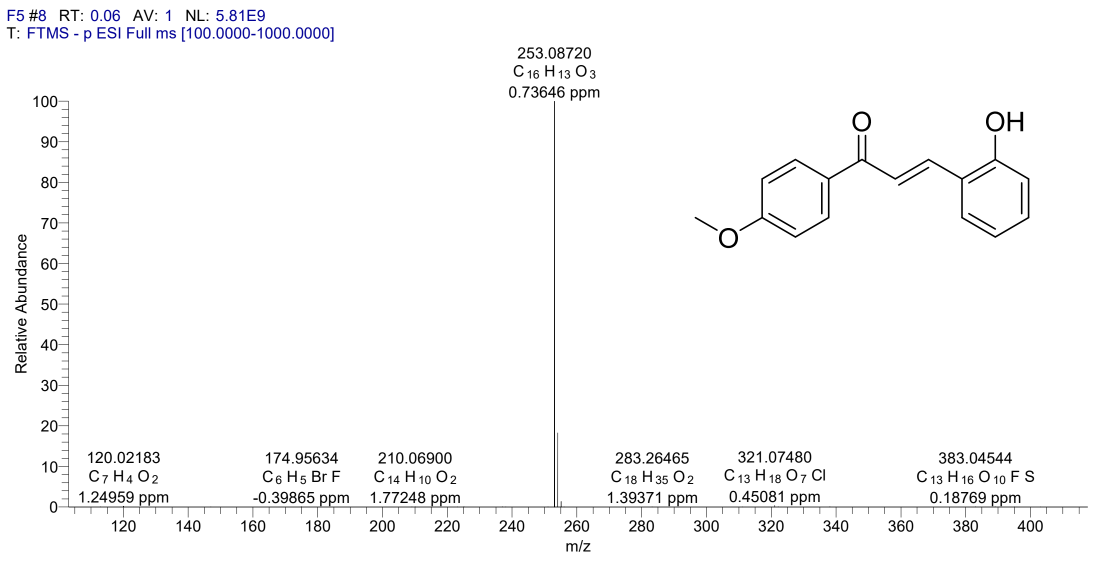

HRMS (ESI) spectrum

The C=O stretching vibration peak at 1642 cm^{-1} and the vibration peaks of O–H groups at 3216 cm^{-1}. The peaks at 3006 and 2970 cm^{-1} could be attributed to –OCH_3. Peaks at 1600, 1583, 1561, and 1510 cm^{-1} could be assigned to the stretching vibration of CH_2 in the aromatic nucleus. The peak at 839 cm^{-1} results from the out-of-plane bending vibration of C–H. The peaks at 1374 and 1341 cm^{-1} are ascribed to the stretching vibration of the C-C benzenoid ring. The peaks at 1094, 1046, and 1021 cm^{-1} are assigned to C–H of the methoxy group. These observations confirmed that the structure is €-3-(2-hydroxyphenyl)-1-(4-methoxyphenyl)propyl-2-en-1-one (**F5**).

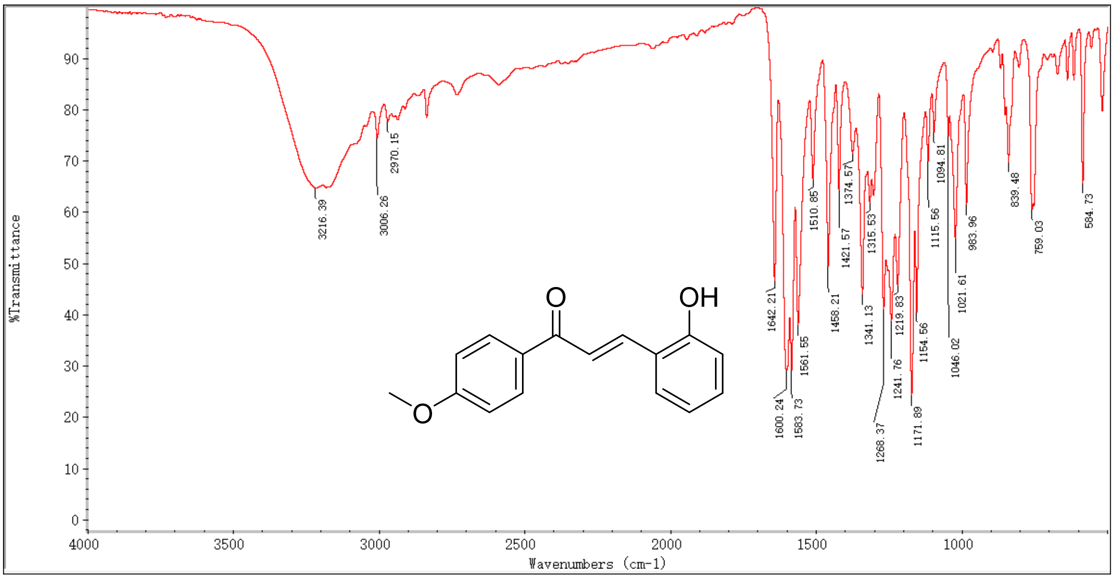

IR spectrum

(E)-3-(3-hydroxyphenyl)-1-(4-methoxyphenyl)propyl-2-en-1-one (F6)

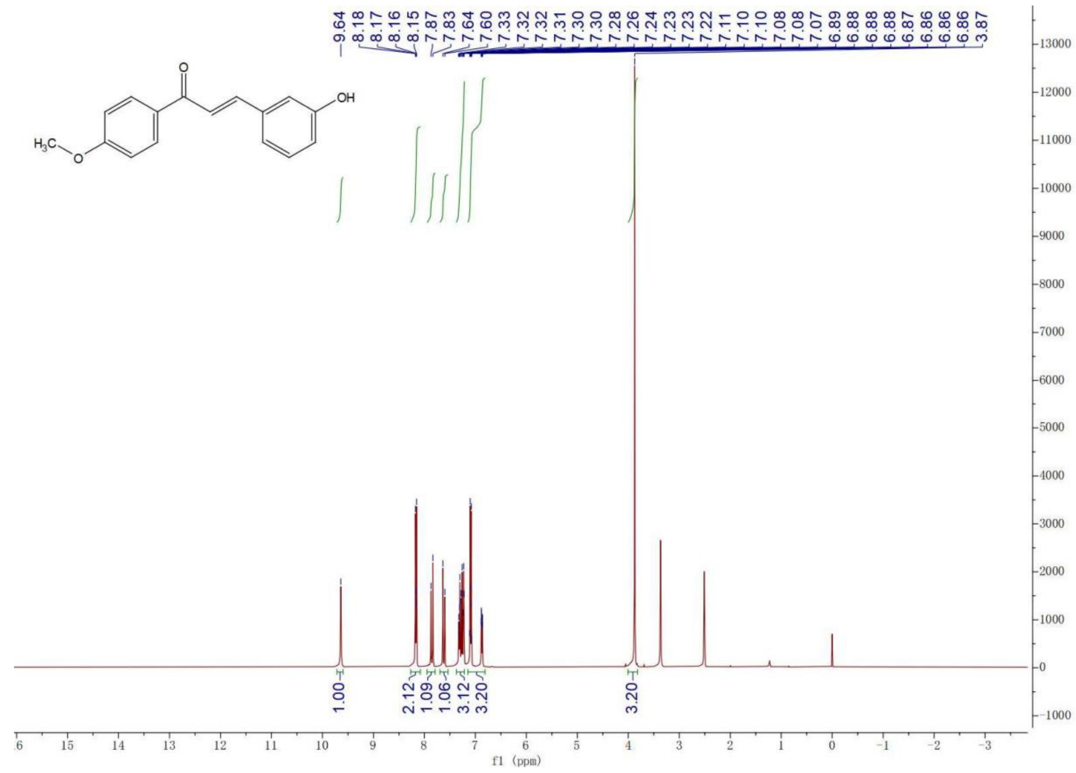

^{1}H NMR spectrum

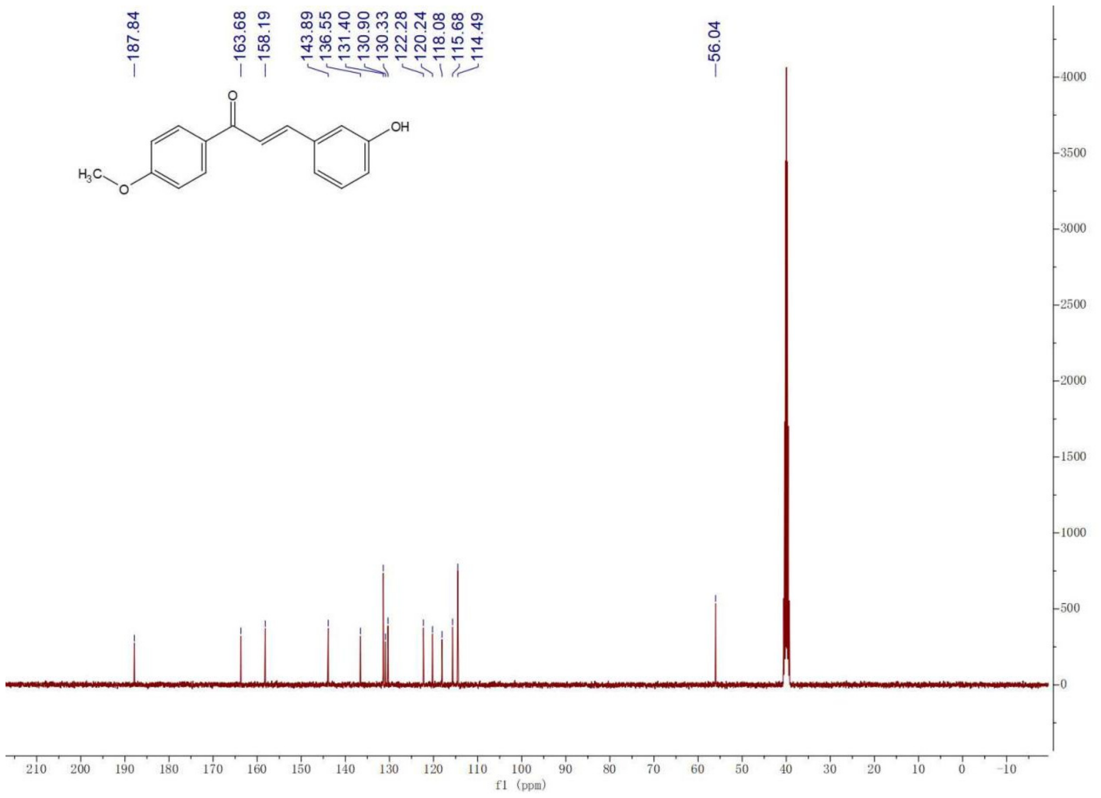

^{13}C NMR spectrum

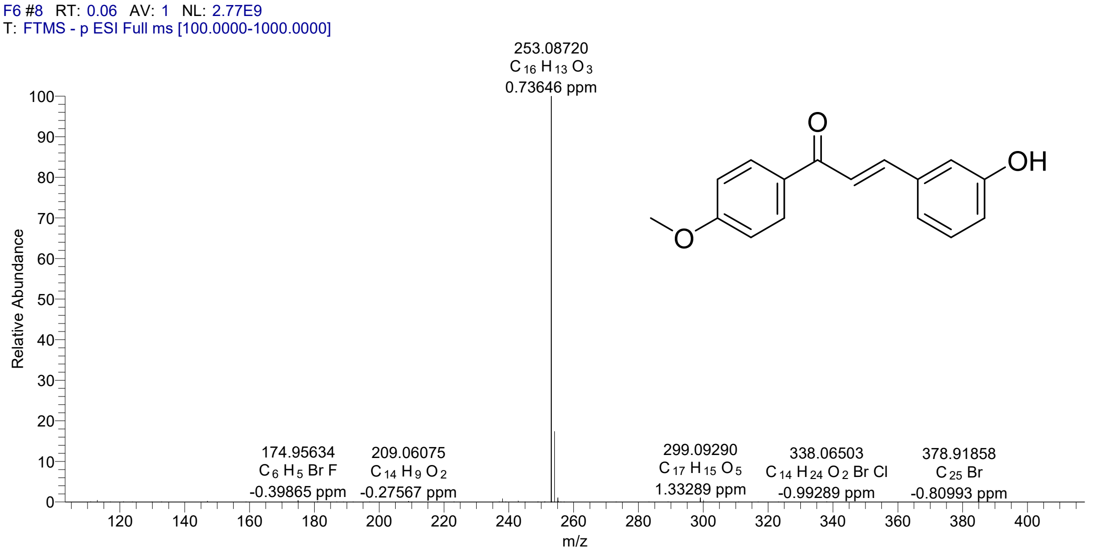

HRMS (ESI) spectrum

The C=O stretching vibration peak at 1650 cm^{-1} and the vibration peaks of O–H groups at 3321 cm^{-1}. The peaks at 2967 and 2841 cm^{-1} could be attributed to $–OCH_3$. Peaks at 1590, 1564, 1510, and 1446 cm^{-1} could be assigned to the stretching vibration of CH_2 in the aromatic nucleus. The peak at 832 cm^{-1} results from the out-of-plane bending vibration of C–H. The peaks at 1366 and 1338 cm^{-1} are ascribed to the stretching vibration of the C-C benzenoid ring. The peaks at 1037, 1021, and 976 cm^{-1} are assigned to C–H of the methoxy group. These observations confirmed that the structure is €-3-(3-hydroxyphenyl)-1-(4-methoxyphenyl)propyl-2-en-1-one (**F6**).

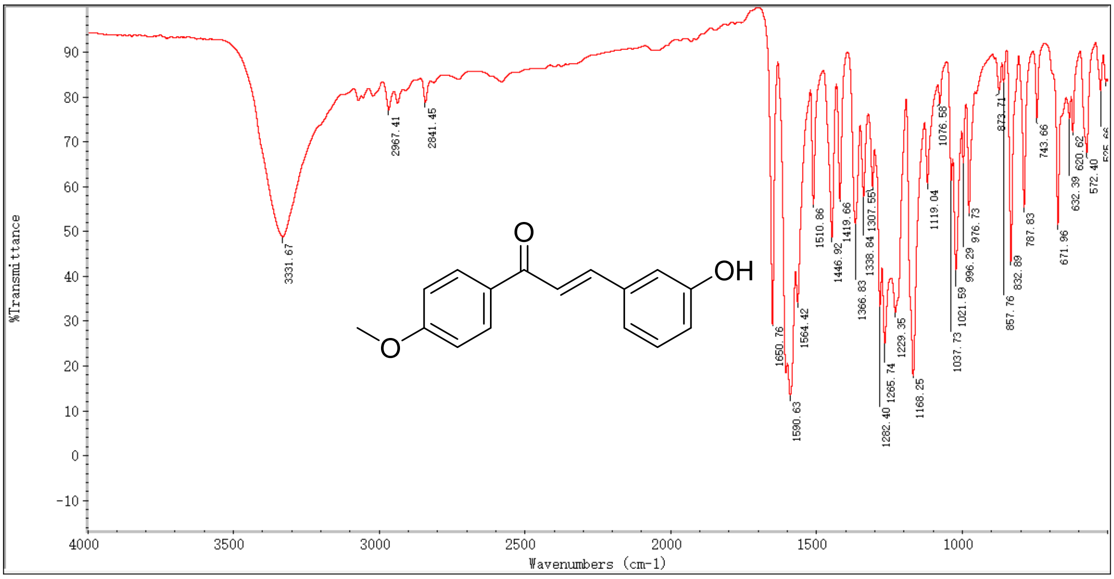

IR spectrum

(E)-1-(2-chlorophenyl)-3-(2-hydroxyphenyl)propyl-2-en-1-one (F7)

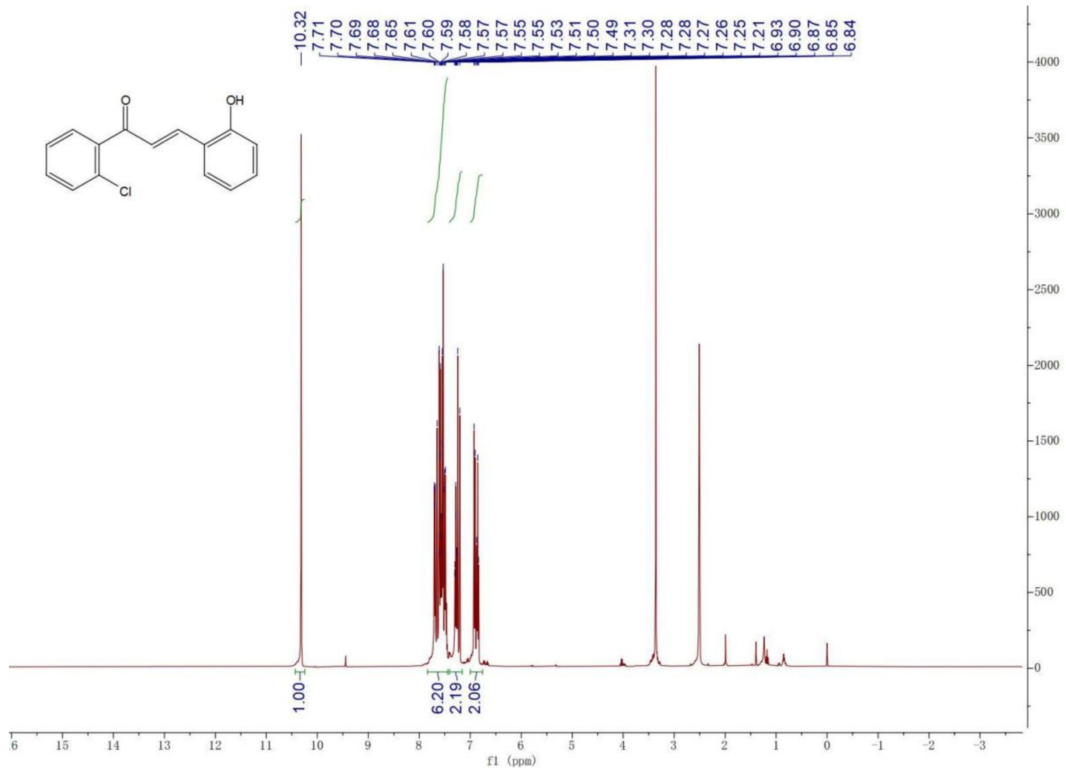

^{1}H NMR spectrum

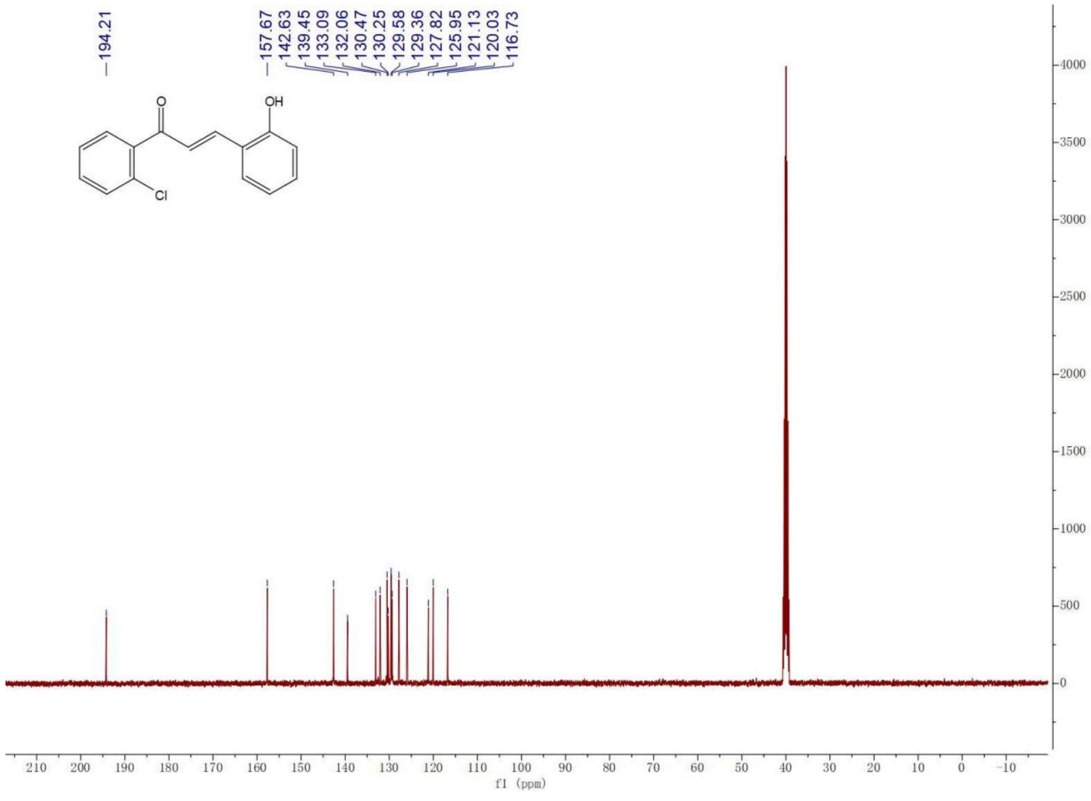

^{13}C NMR spectrum

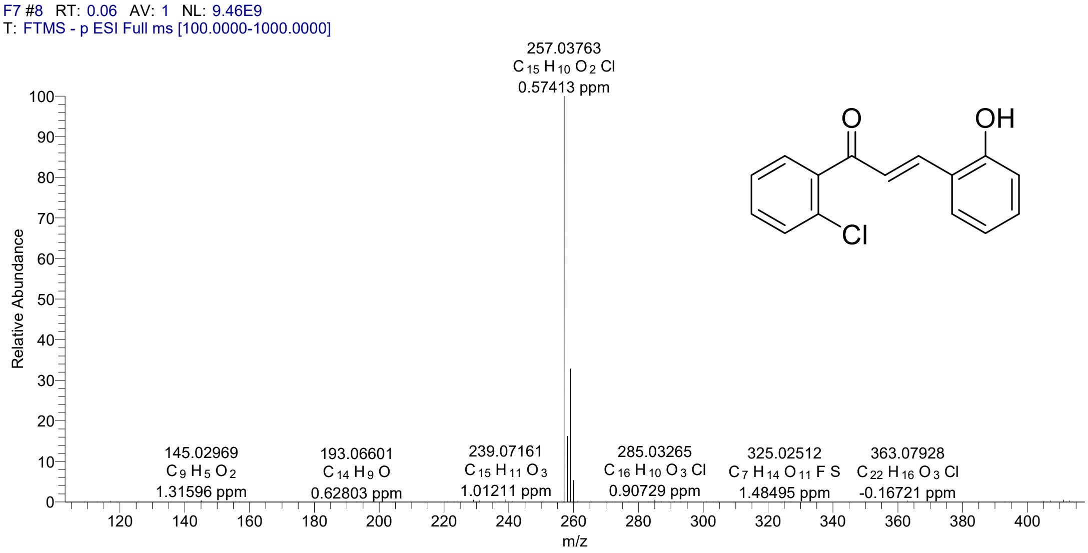

HRMS (ESI) spectrum

The C=O stretching vibration peak at 1648 cm-1 and the vibration peaks of O–H groups at 3217 cm-1. Peaks at 1589, 1563, 1498, and 1470 cm-1 could be assigned to the stretching vibration of CH2 in the aromatic nucleus. The peak at 836 cm−1 results from the out-of-plane bending vibration of C–H. The peaks at 1354 and 1335 cm−1 are ascribed to the stretching vibration of the C-C benzenoid ring. The peak at 748 cm−1 is assigned to the stretching vibration of the C-Cl. These observations confirmed that the structure is €-1-(2-chlorophenyl)-3-(2-hydroxyphenyl)propyl-2-en-1-one (**F7**).

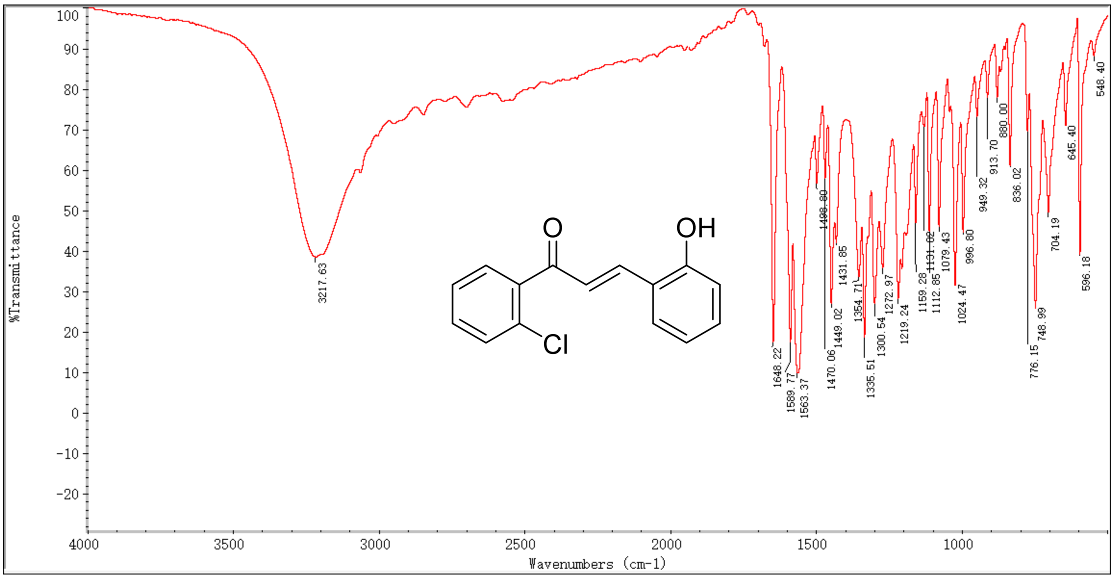

IR spectrum

(E)-1-(4-chlorophenyl)-3-(2-hydroxyphenyl)propyl-2-en-1-one（F8）

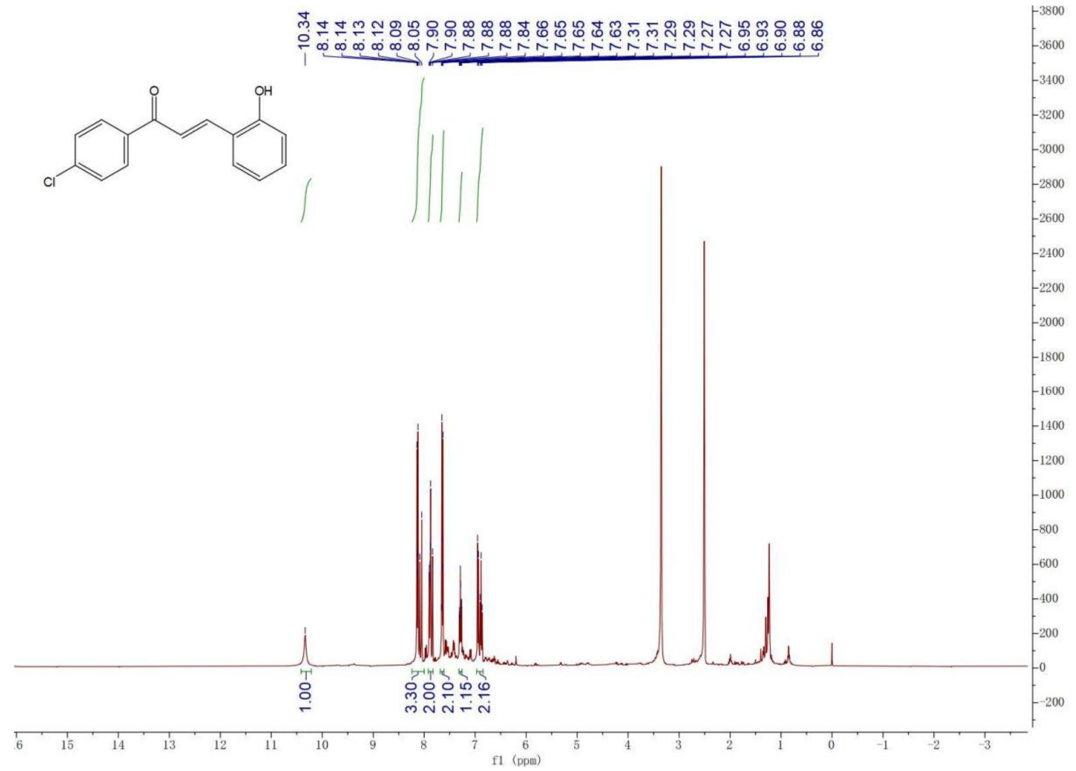

^{1}H NMR spectrum

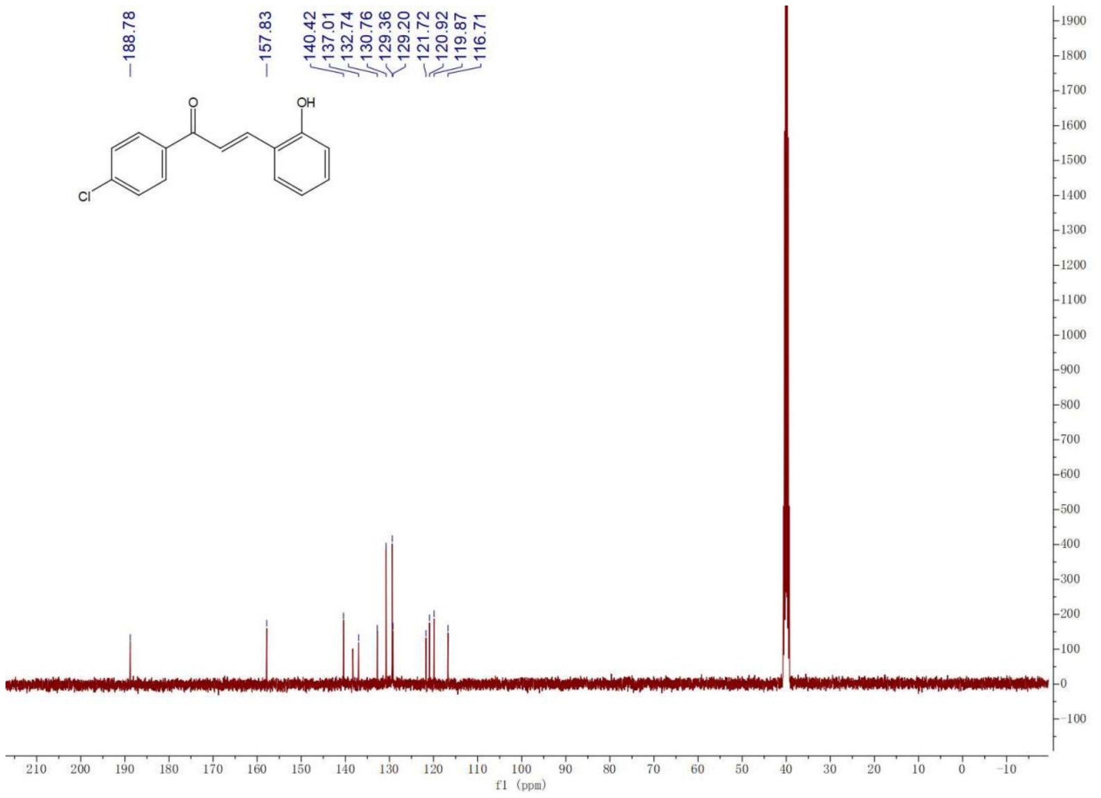

^{13}C NMR spectrum

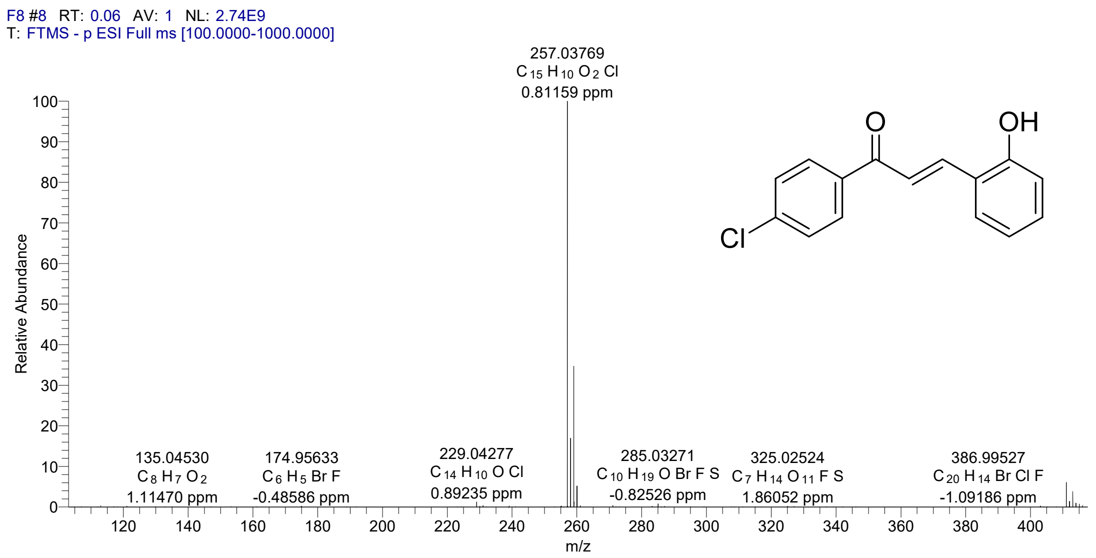

HRMS (ESI) spectrum

(E)-1-(4-bromophenyl)-3-(2-hydroxyphenyl)propyl-2-en-1-one (F9)

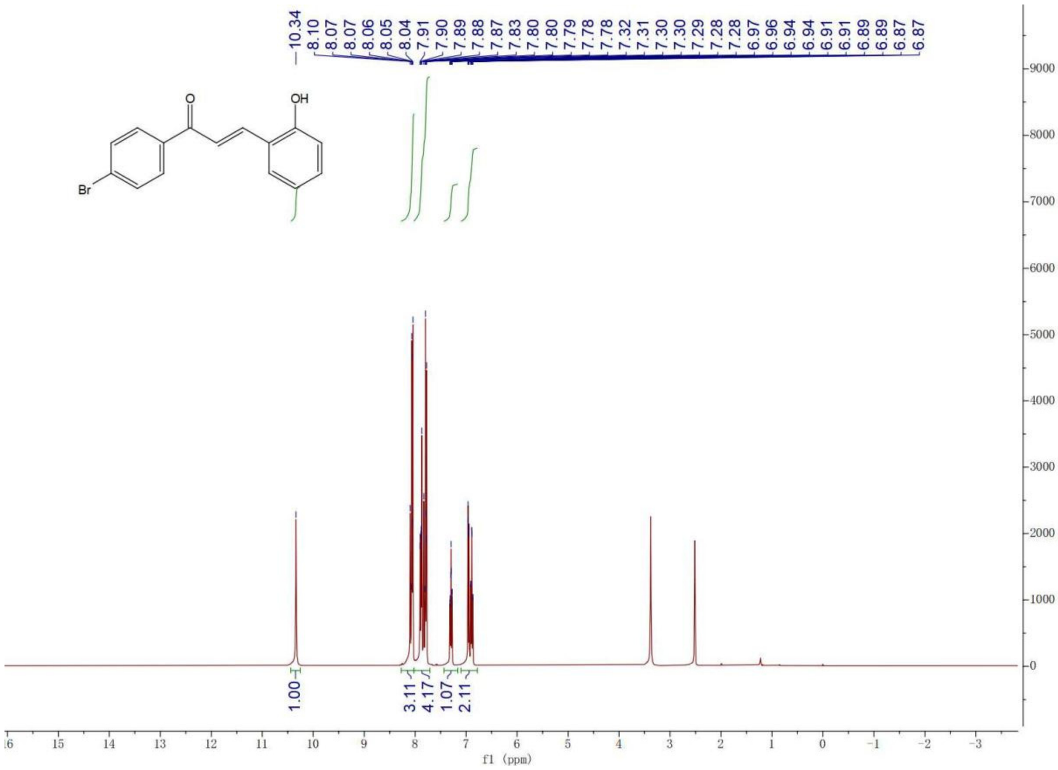

^{1}H NMR spectrum

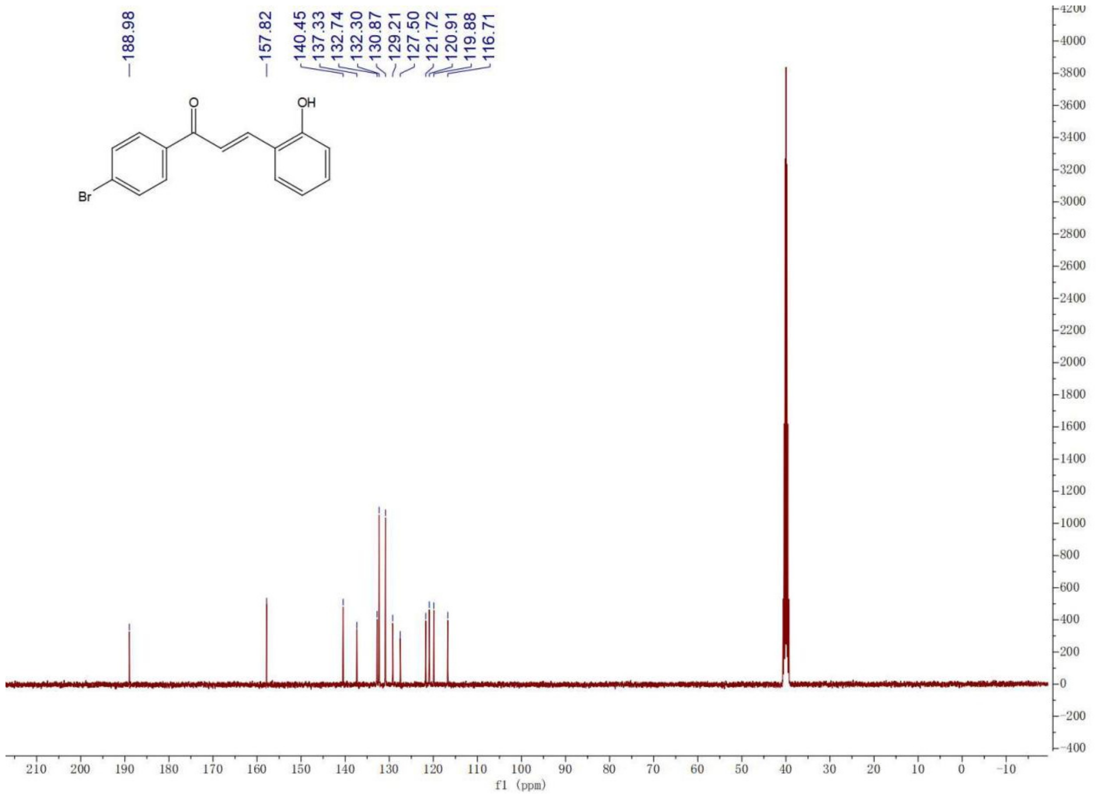

^{13}C NMR spectrum

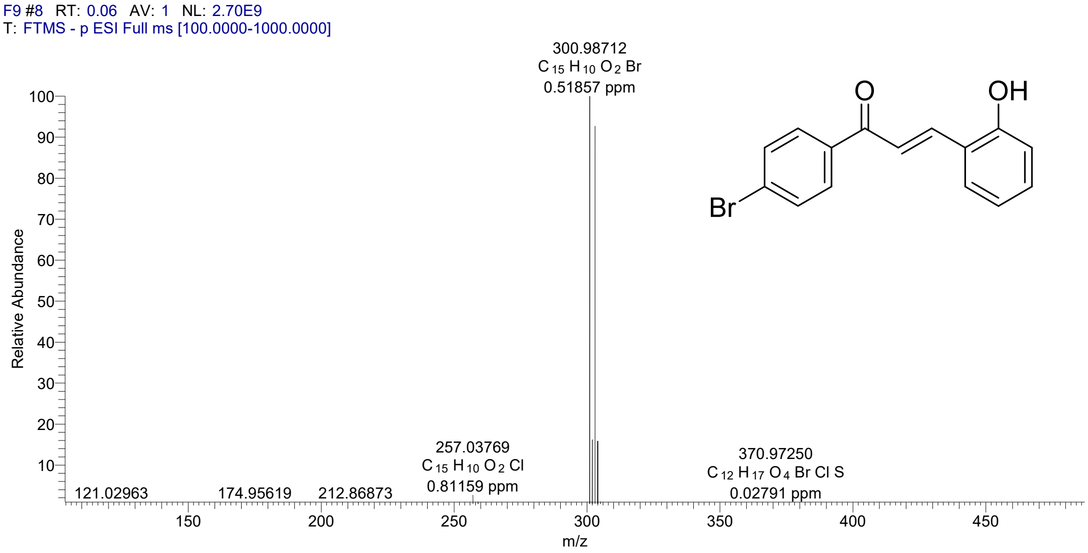

HRMS (ESI) spectrum

The C=O stretching vibration peak at 1643 cm-1 and the vibration peaks of O–H groups at 3219 cm-1. Peaks at 1584, 1554, 1484, and 1448 cm-1 could be assigned to the stretching vibration of CH_2 in the aromatic nucleus. The peak at 832 cm−1 results from the out-of-plane bending vibration of C–H. The peaks at 1338 and 1304cm−1 are ascribed to the stretching vibration of the C-C benzenoid ring. The peak at 749 cm−1 is assigned to the stretching vibration of the C-Br. These observations confirmed that the structure is€-1-(4-bromophenyl)-3-(2-hydroxyphenyl)propyl-2-en-1-one (**F9**).

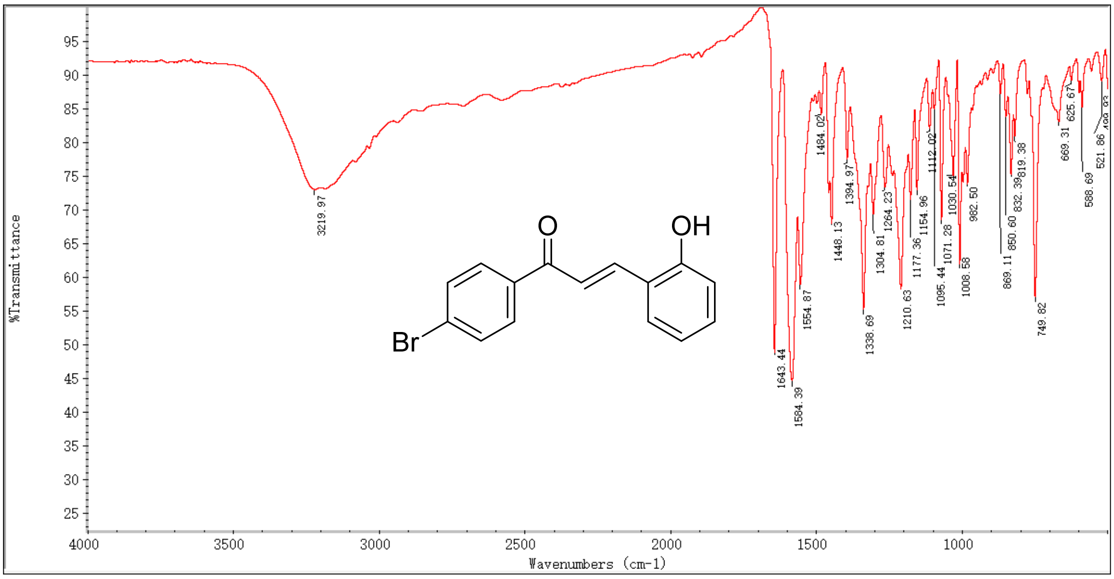

IR spectrum

(E)-1-(4-bromophenyl)-3-(3-hydroxyphenyl)propyl-2-en-1-one (F10)

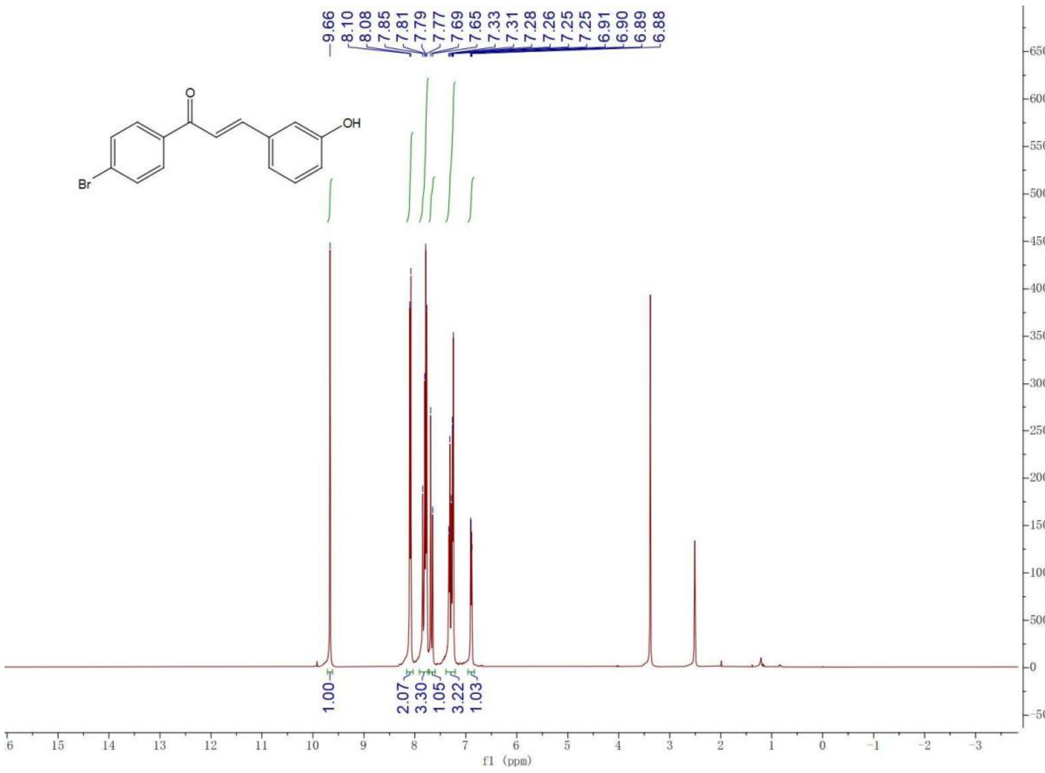

^{1}H NMR spectrum

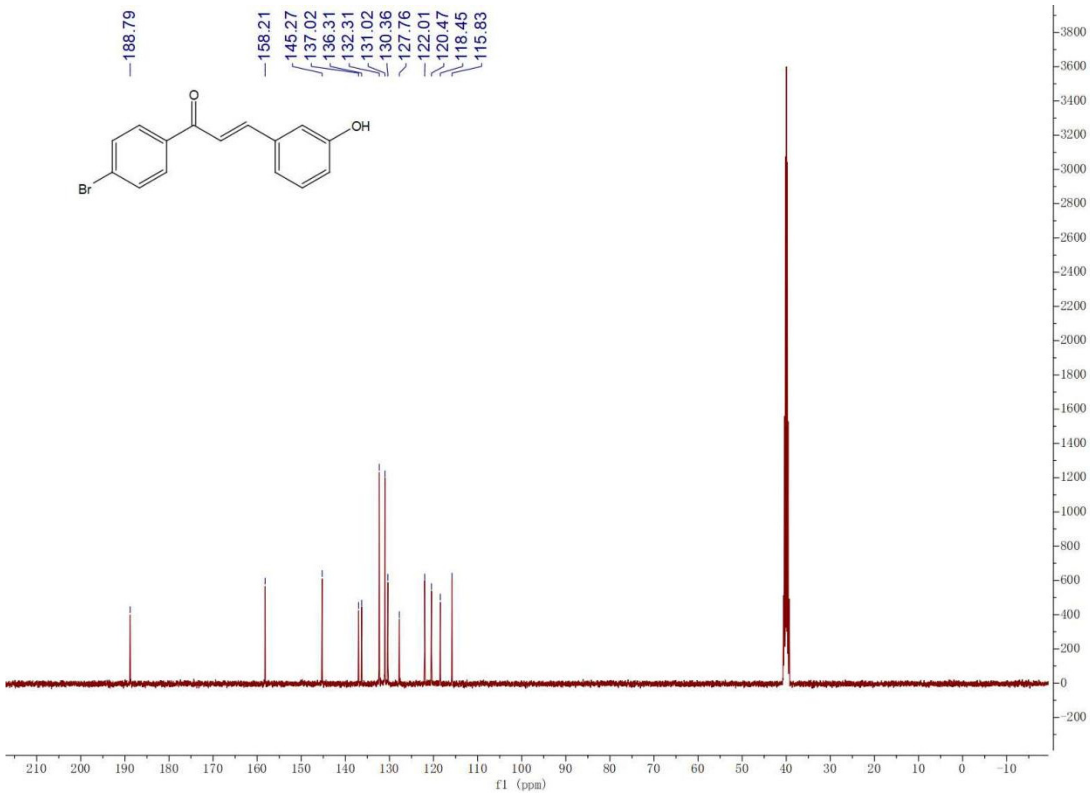

^{13}C NMR spectrum

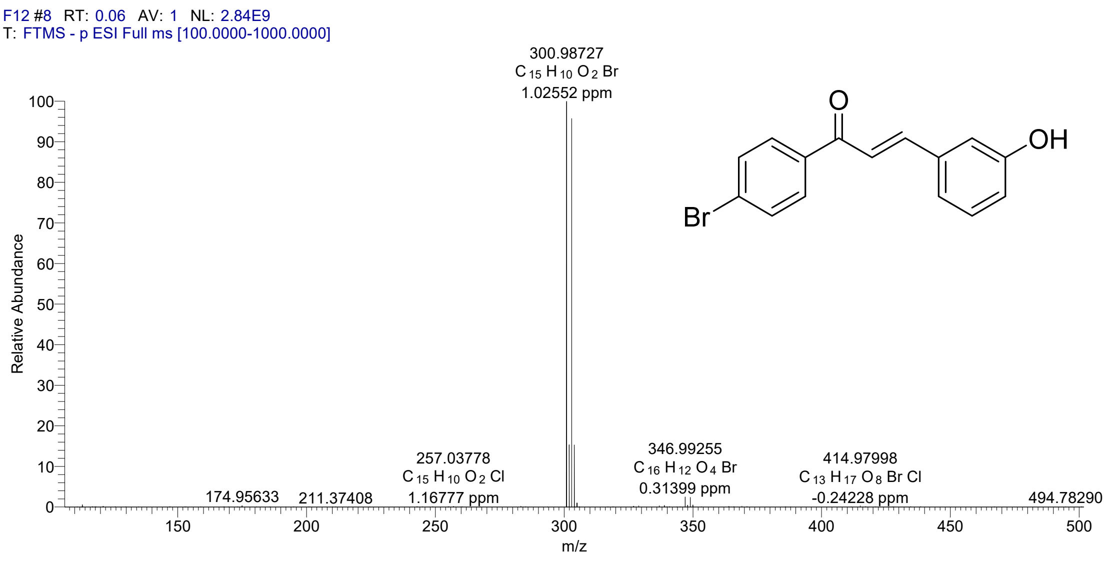

HRMS (ESI) spectrum

The C=O stretching vibration peak at 1653 cm^{-1} and the vibration peaks of O–H groups at 3396 cm^{-1}. Peaks at 1605, 190, 1483, and 1448 cm^{-1} could be assigned to the stretching vibration of CH_2 in the aromatic nucleus. The peak at 825 cm^{-1} results from the out-of-plane bending vibration of C–H. The peaks at 1394 and 1361cm^{-1} are ascribed to the stretching vibration of the C-C benzenoid ring. The peak at 793 cm^{-1} is assigned to the stretching vibration of the C-Br. These observations confirmed that the structure is (E)-1-(4-bromophenyl)-3-(3-hydroxyphenyl)propyl-2-en-1-one (**F10**).

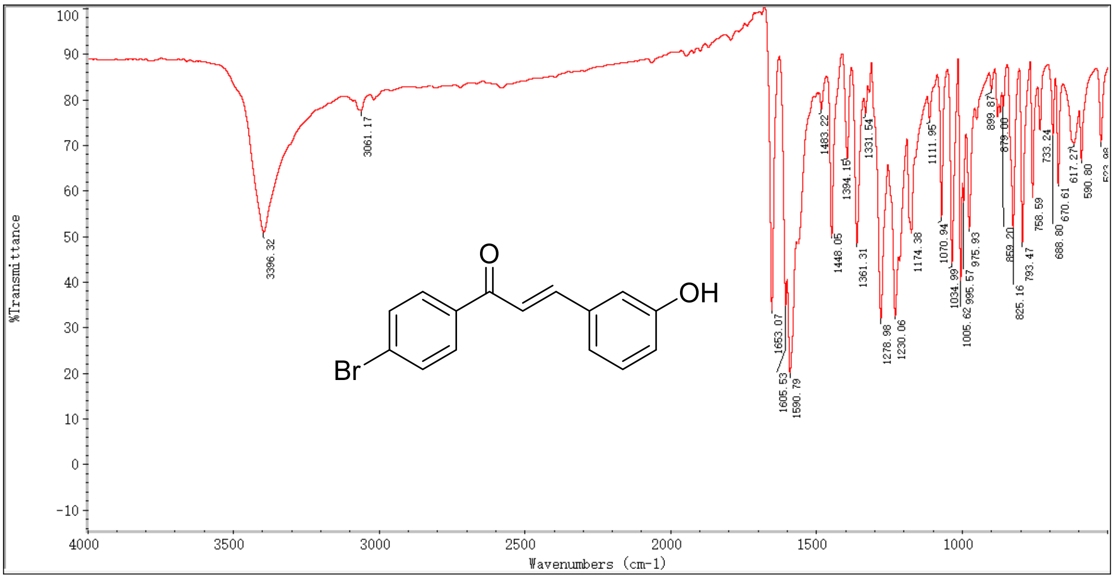

IR spectrum

(E)-1-(2,4-dimethylphenyl)-3-(2-hydroxyphenyl)propyl-2-en-1-one (F11)

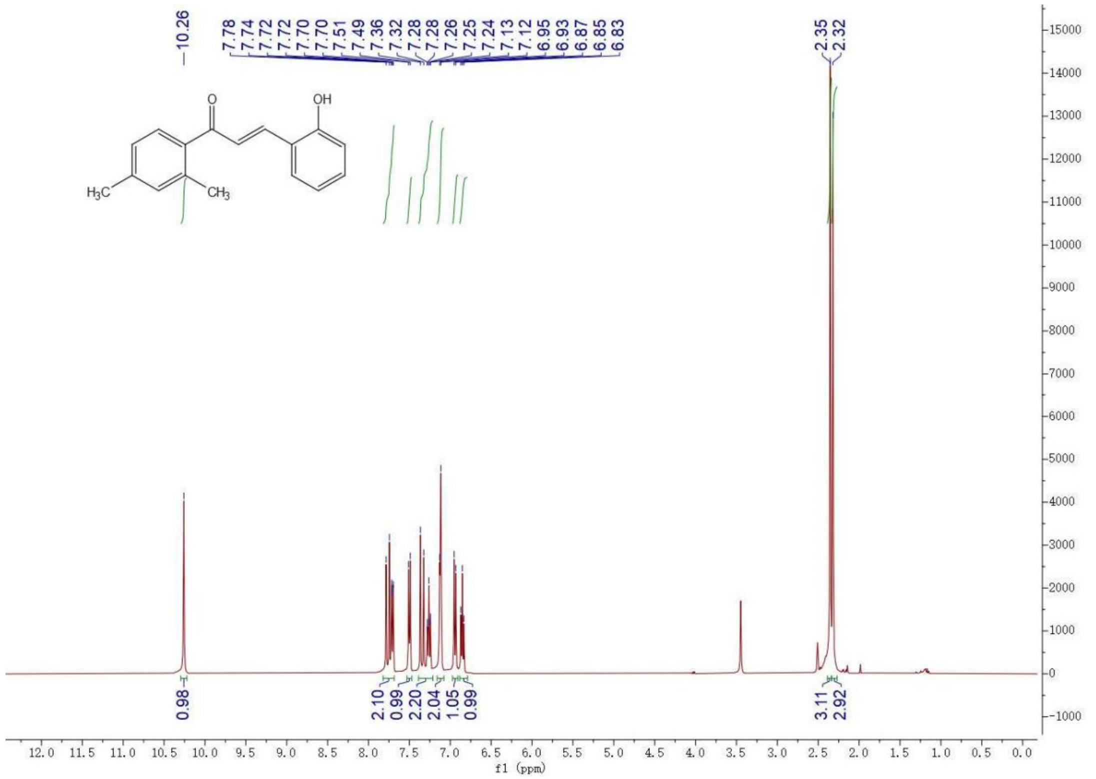

^{1}H NMR spectrum

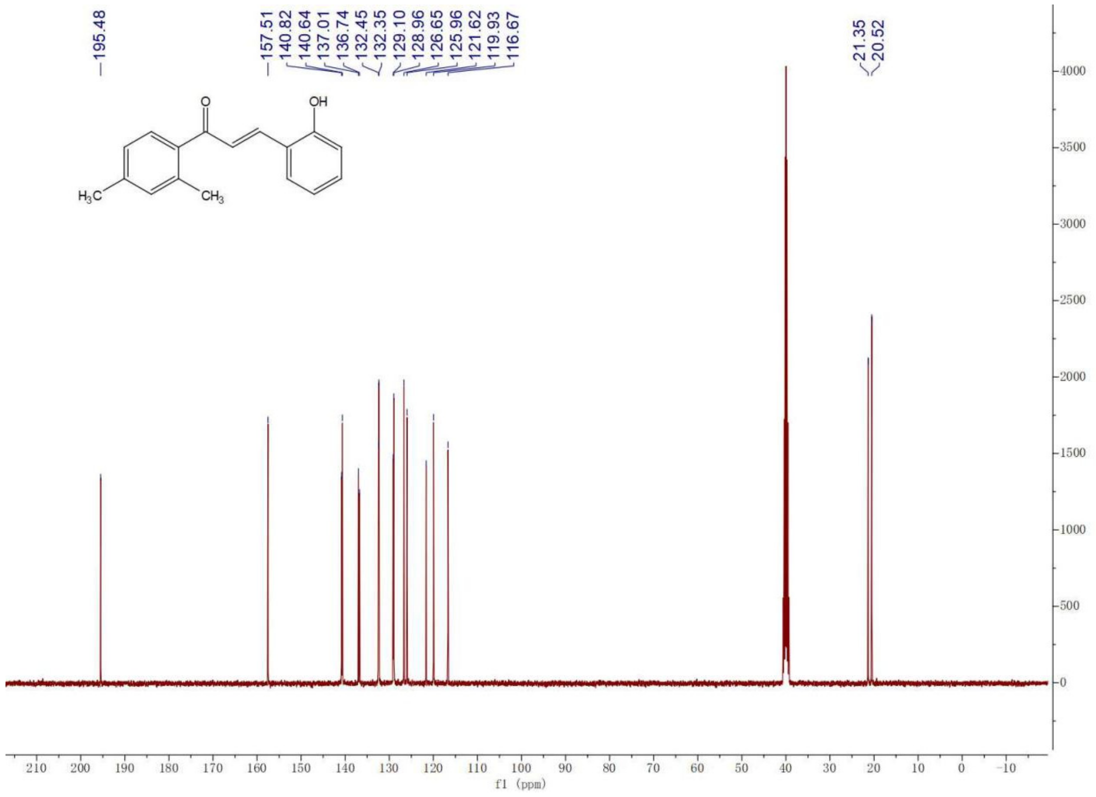

^{13}C NMR spectrum

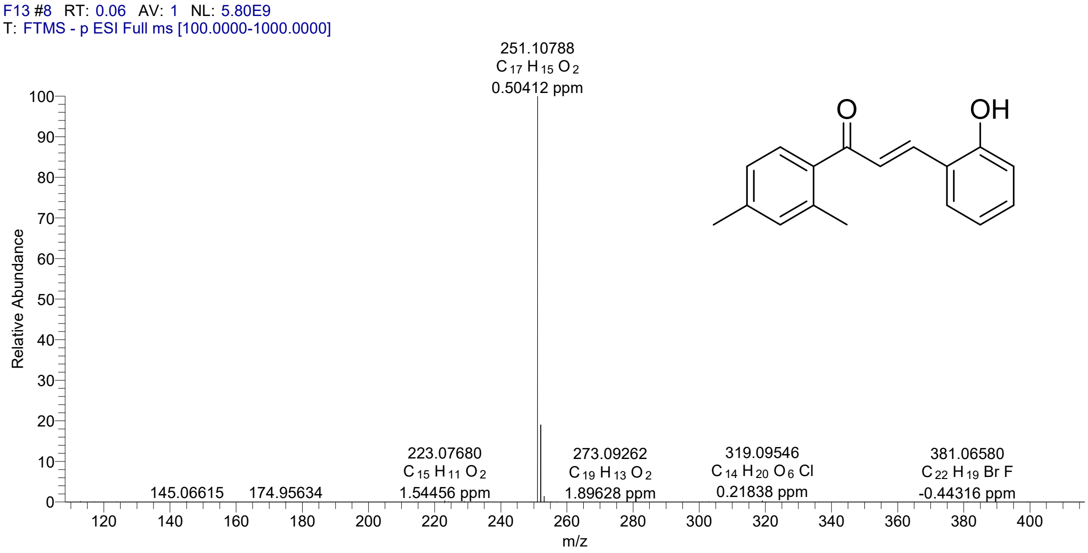

HRMS (ESI) spectrum

The C=O stretching vibration peak at 1640 cm^{-1} and the vibration peaks of O–H groups at 3181 cm^{-1}. Peaks at 1555, 147, and 1450 cm^{-1} could be assigned to the stretching vibration of CH_2 in the aromatic nucleus. The peak at 822 cm^{-1} results from the out-of-plane bending vibration of C–H. The peaks at 1357 and 1336 cm^{-1} are ascribed to the stretching vibration of the C-C benzenoid ring. The peaks at 1025 and 996 cm^{-1} are assigned to C–H of the methyl group. These observations confirmed that the structure is (E)-1-(2,4-dimethylphenyl)-3-(2-hydroxyphenyl)propyl-2-en-1-one (**F11**).

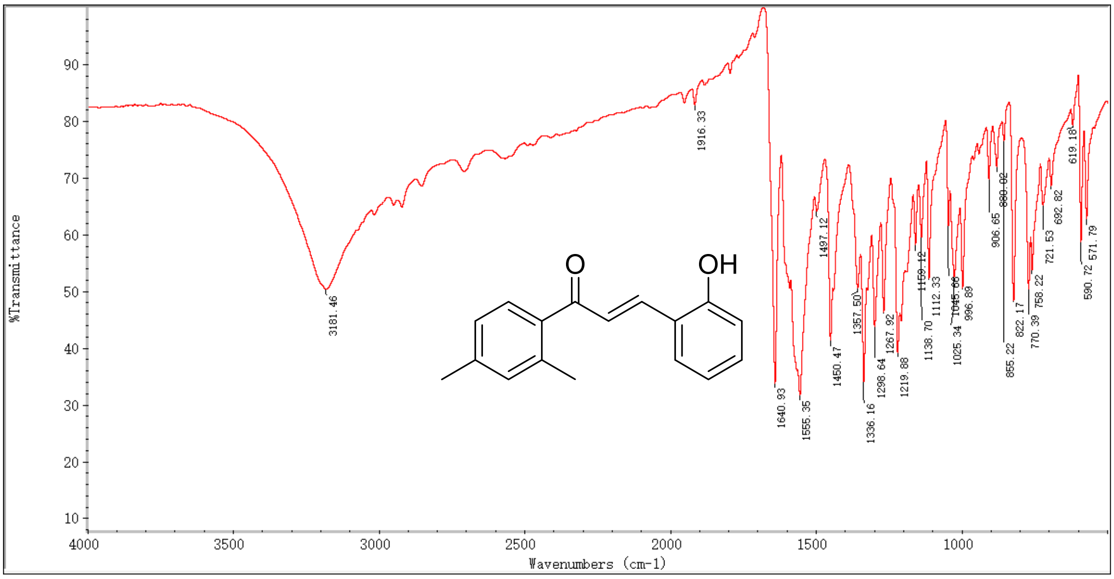

IR spectrum

(E)-1-(3,4-dimethylphenyl)-3-(3-hydroxyphenyl)propyl-2-en-1-one (F12)

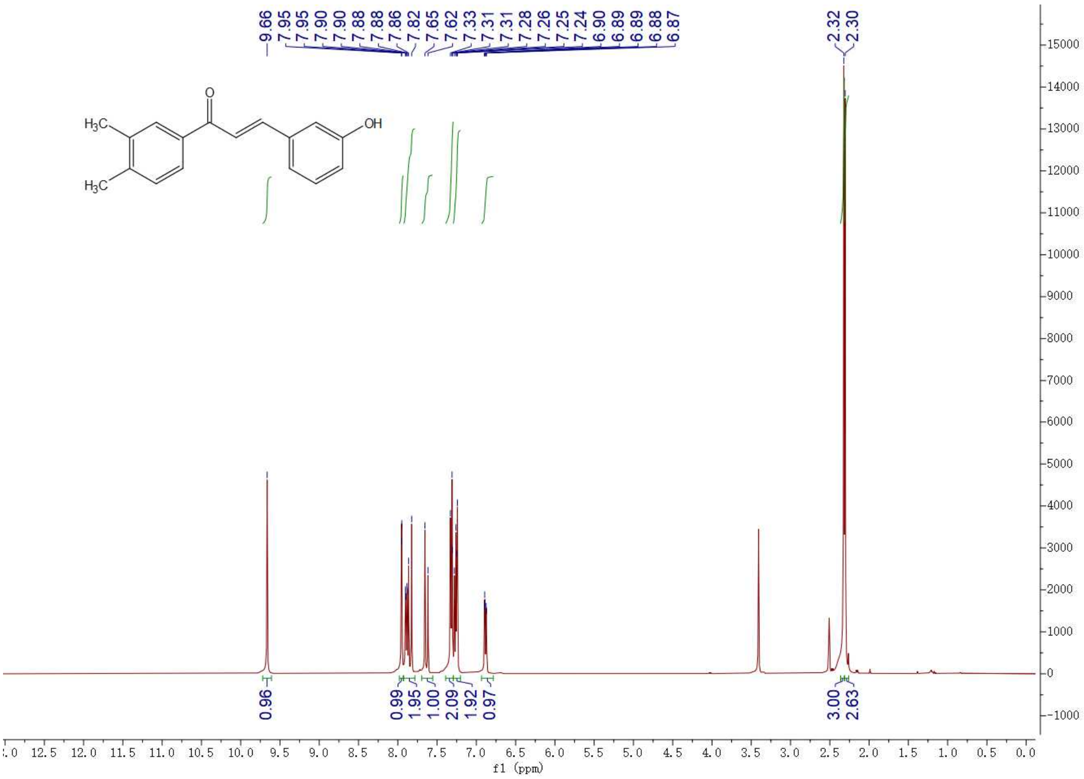

^{1}H NMR spectrum

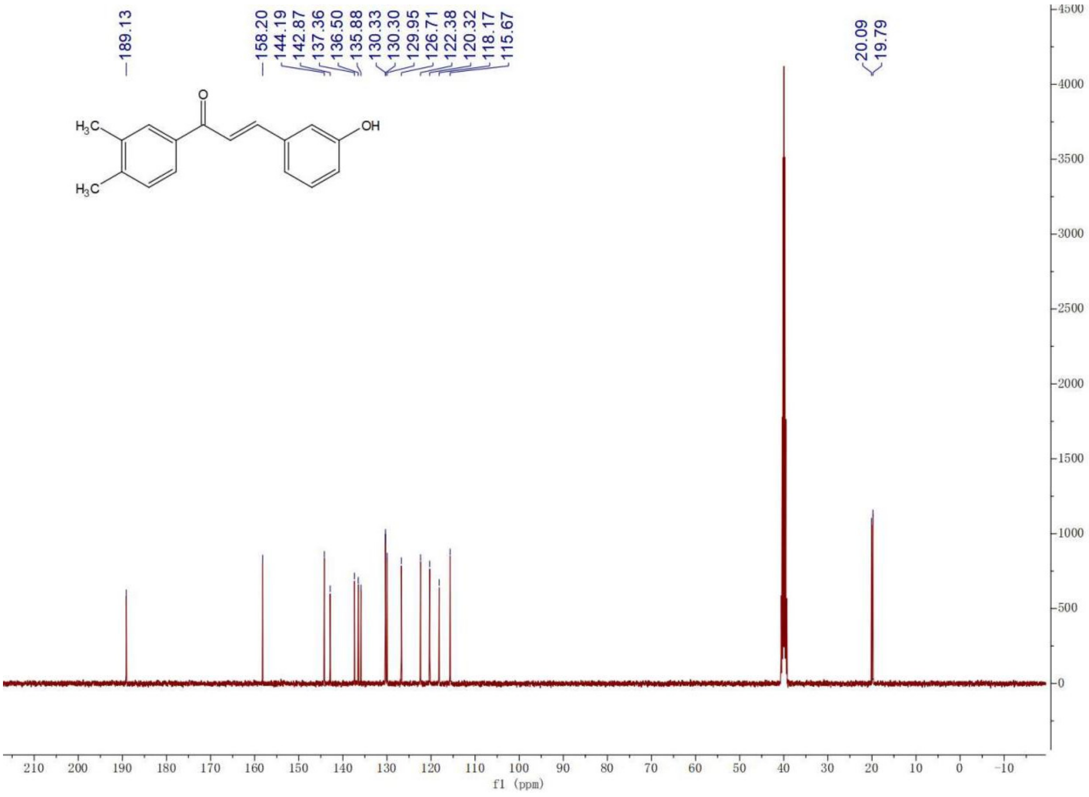

^{13}C NMR spectrum

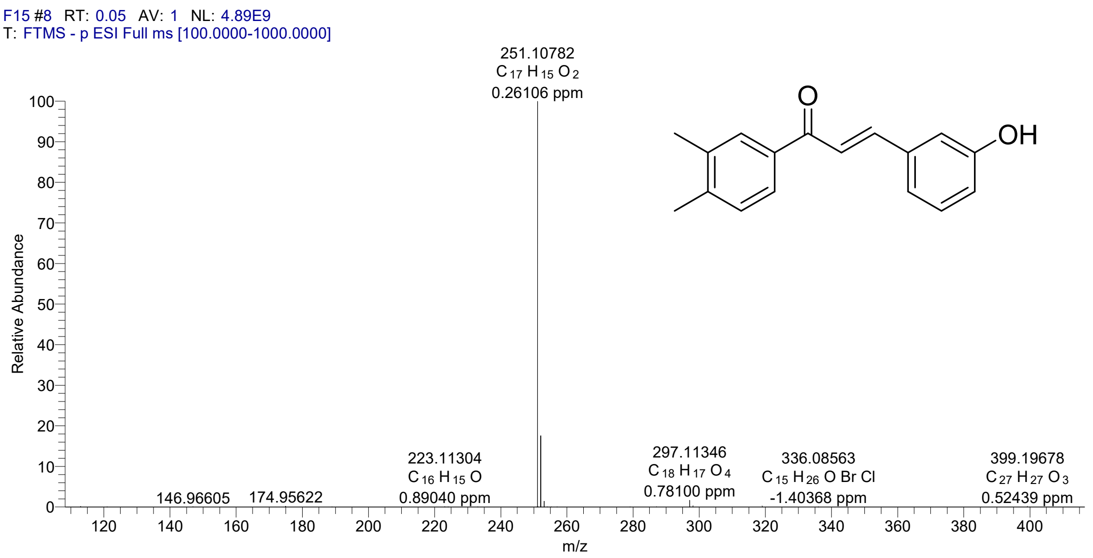

HRMS (ESI) spectrum

The C=O stretching vibration peak at 1651 cm^{-1} and the vibration peaks of O–H groups at 3348 cm^{-1}. The peaks at 3071, 2970, and 2919 cm^{-1} could be attributed to the stretching vibration of C–H in CH_3. Peaks at 1612, 1593, and 1566 cm^{-1} could be assigned to the stretching vibration of CH_2 in the aromatic nucleus. The peak at 809 cm^{-1} results from the out-of-plane bending vibration of C–H. The peaks at 1362 and 1306 cm^{-1} are ascribed to the stretching vibration of the C-C benzenoid ring. The peaks at 994 and 975 cm^{-1} are assigned to C–H of the methyl group. These observations confirmed that the structure is (E)-1-(3,4-dimethylphenyl)-3-(3-hydroxyphenyl)propyl-2-en-1-one (**F12**).

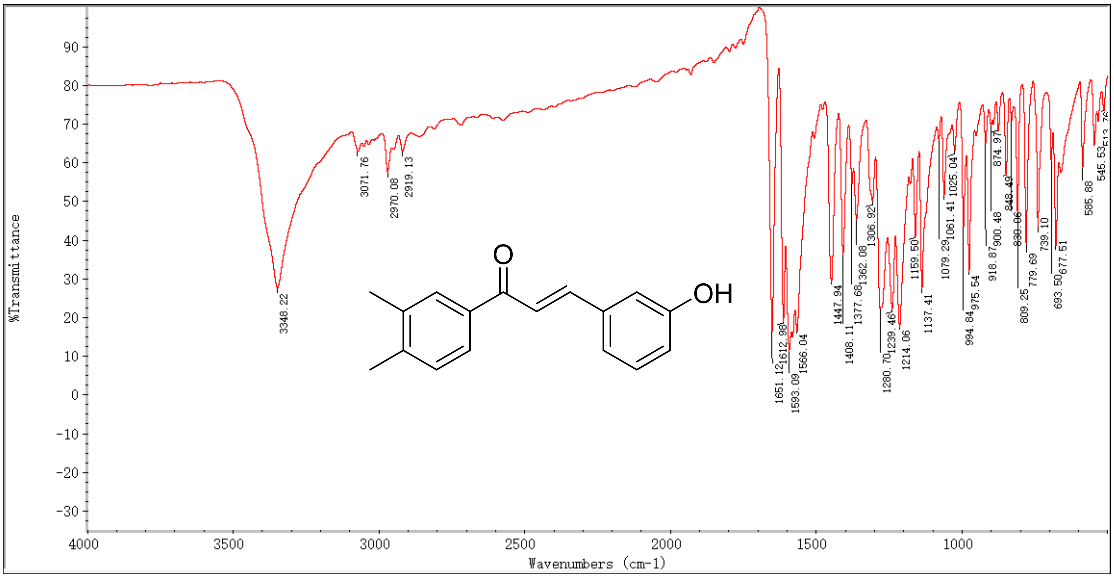

IR spectrum

(E)-1-(3-fluorophenyl)-3-(3-hydroxyphenyl)propyl-2-en-1-one(F13)

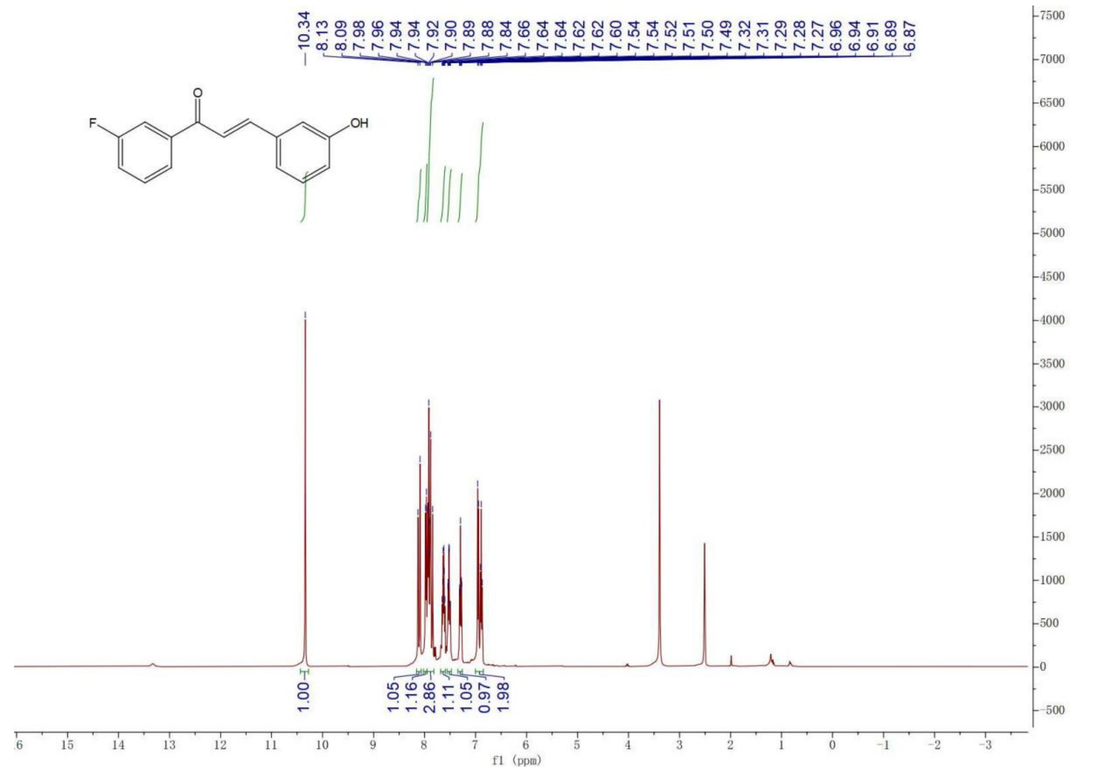

^{1}H NMR spectrum

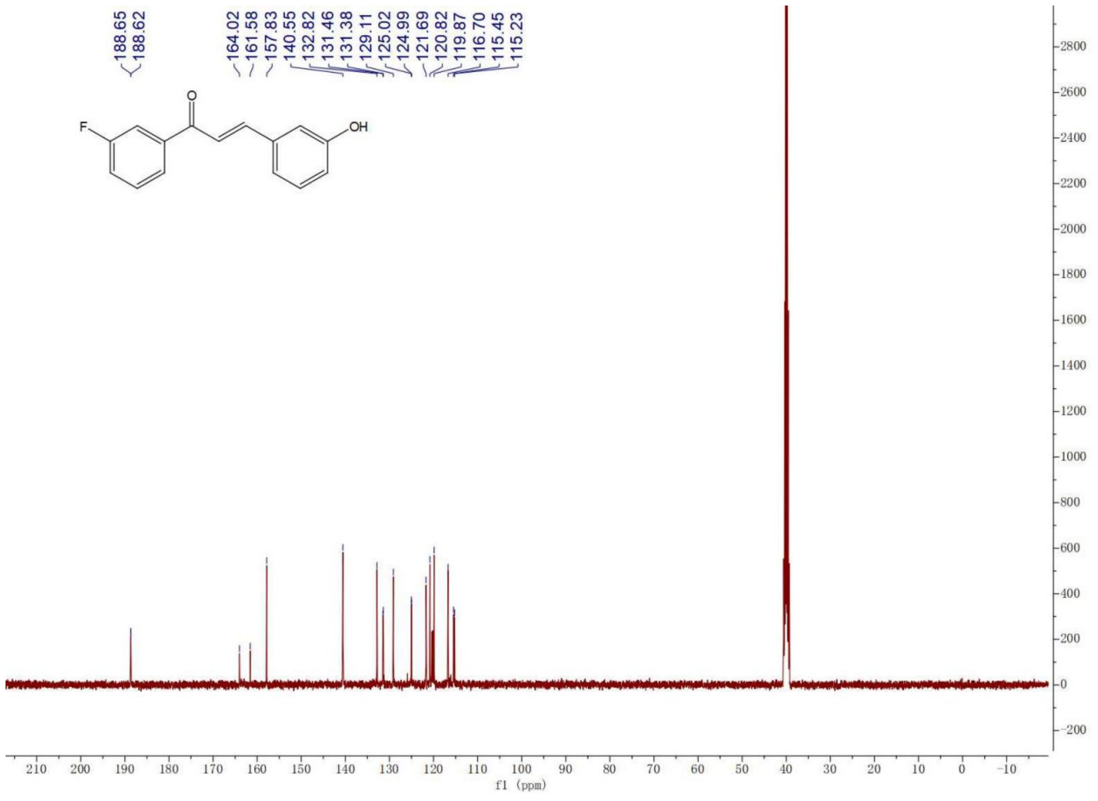

^{13}C NMR spectrum

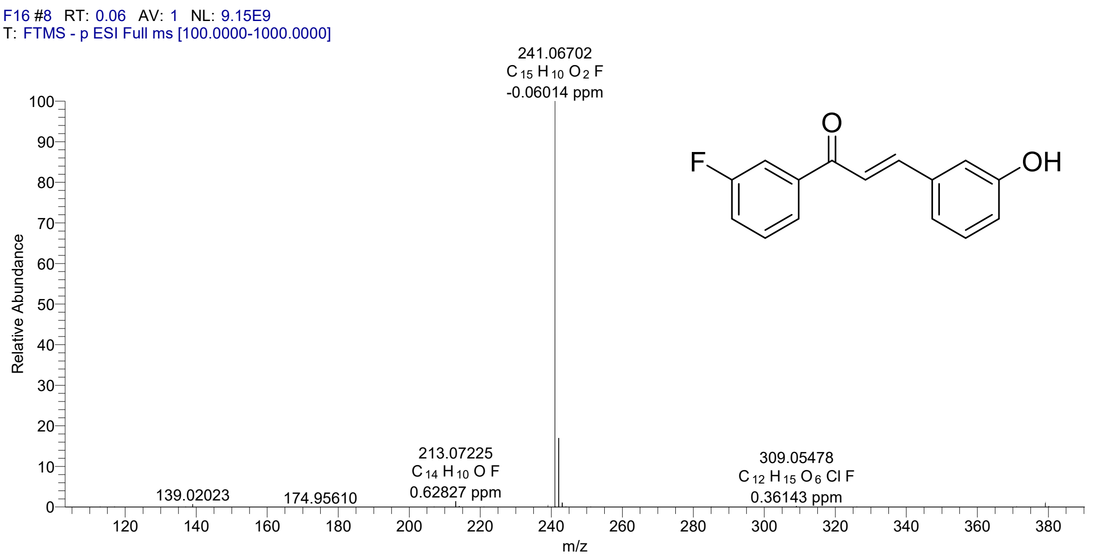

HRMS (ESI) spectrum

The C=O stretching vibration peak at 1648 cm^{-1} and the vibration peaks of O–H groups at 3164 cm^{-1}. Peaks at 1602, 1570, 1487, and 1460 cm^{-1} could be assigned to the stretching vibration of CH_2 in the aromatic nucleus. The peak at 832 cm^{-1} results from the out-of-plane bending vibration of C–H. The peaks at 1346 and 1305 cm^{-1} are ascribed to the stretching vibration of the C-C benzenoid ring. The peak at 755 cm^{-1} is assigned to the stretching vibration of the C-F. These observations confirmed that the structure is(E)-1-(3-fluorophenyl)-3-(3-hydroxyphenyl)propyl-2-en-1-one (**F16**).

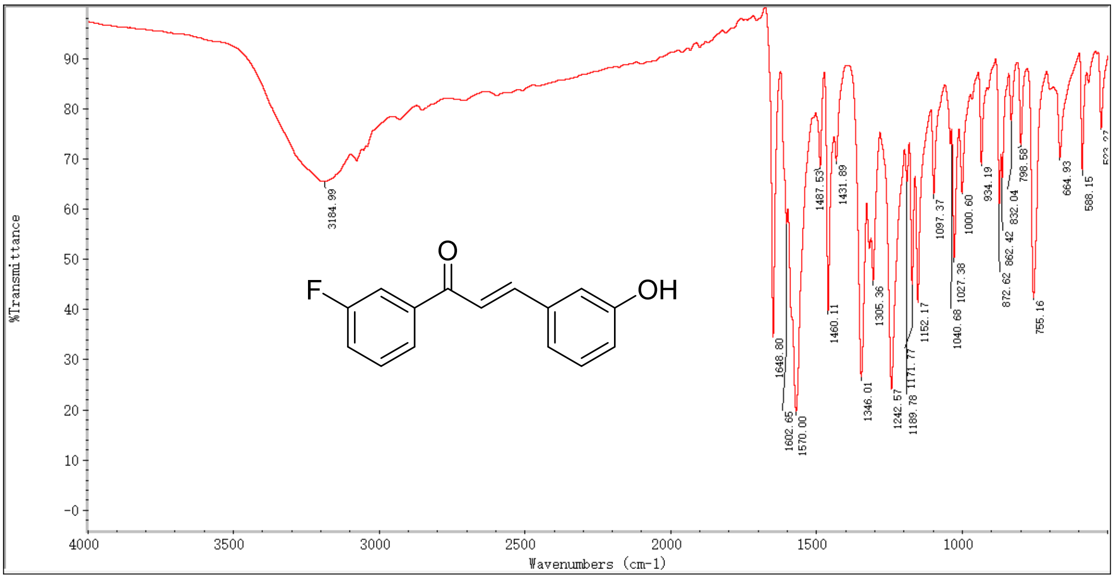

IR spectrum

(E)-1-(2,4-dimethylphenyl)-3-(3-hydroxyphenyl)propyl-2-en-1-one (F14)

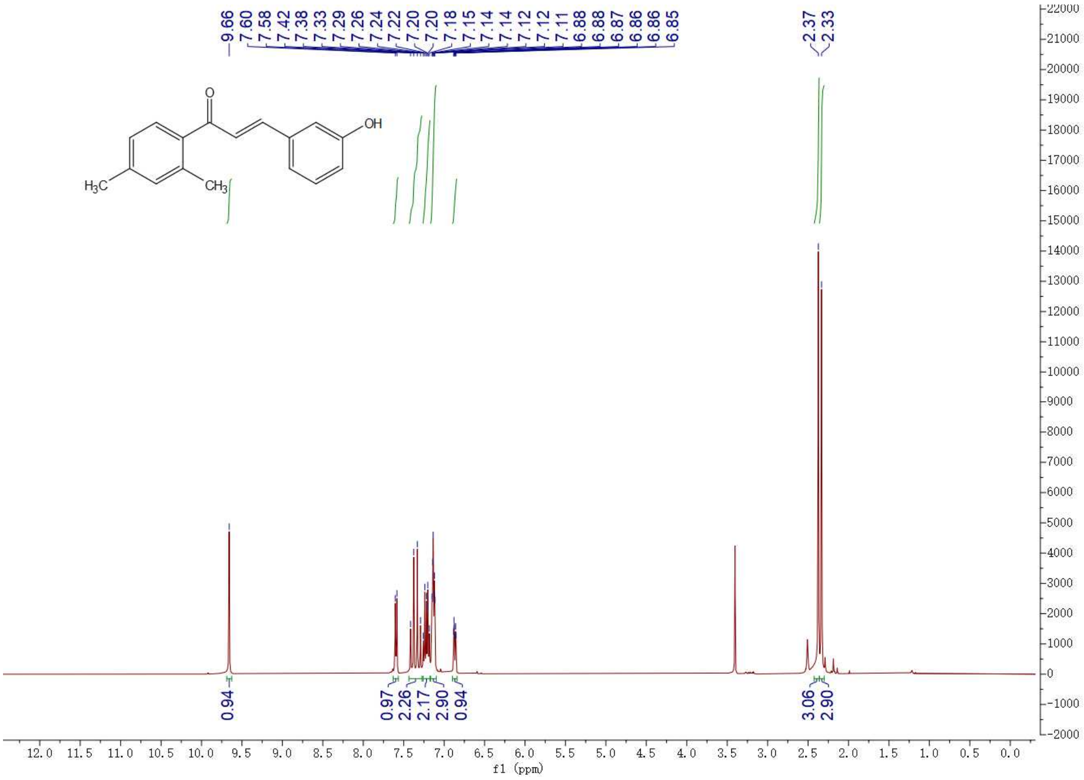

^{1}H NMR spectrum

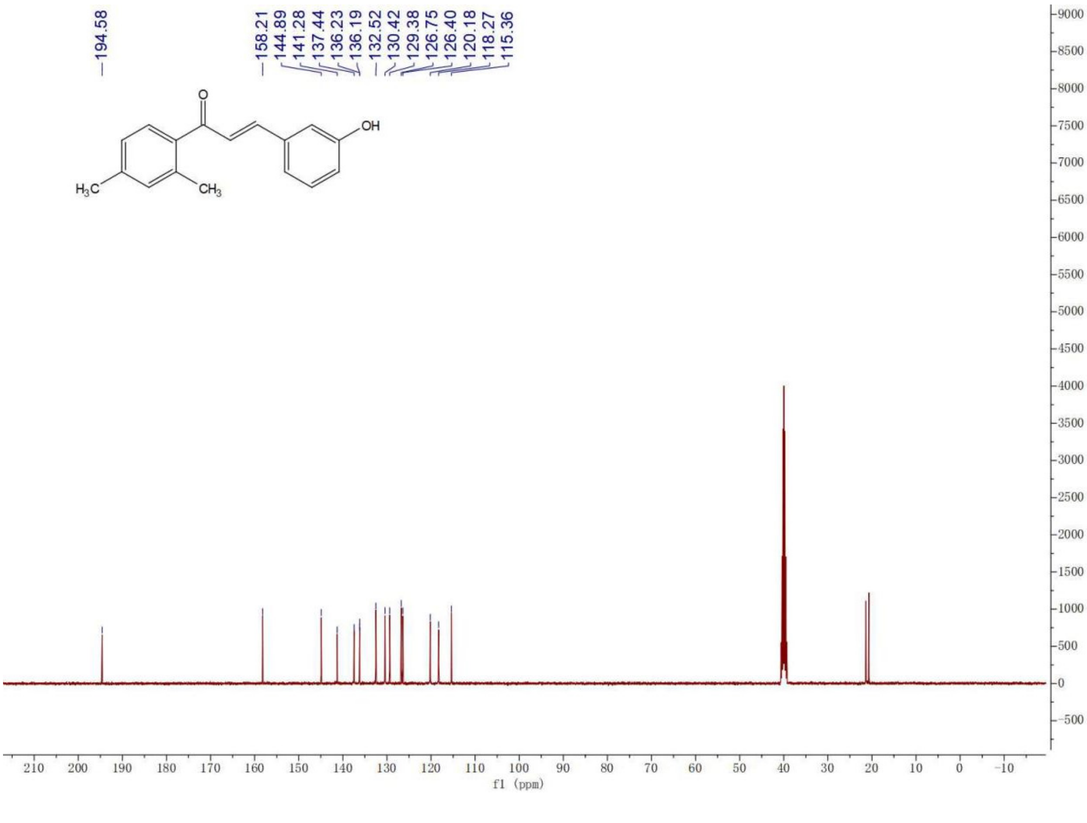

^{13}C NMR spectrum

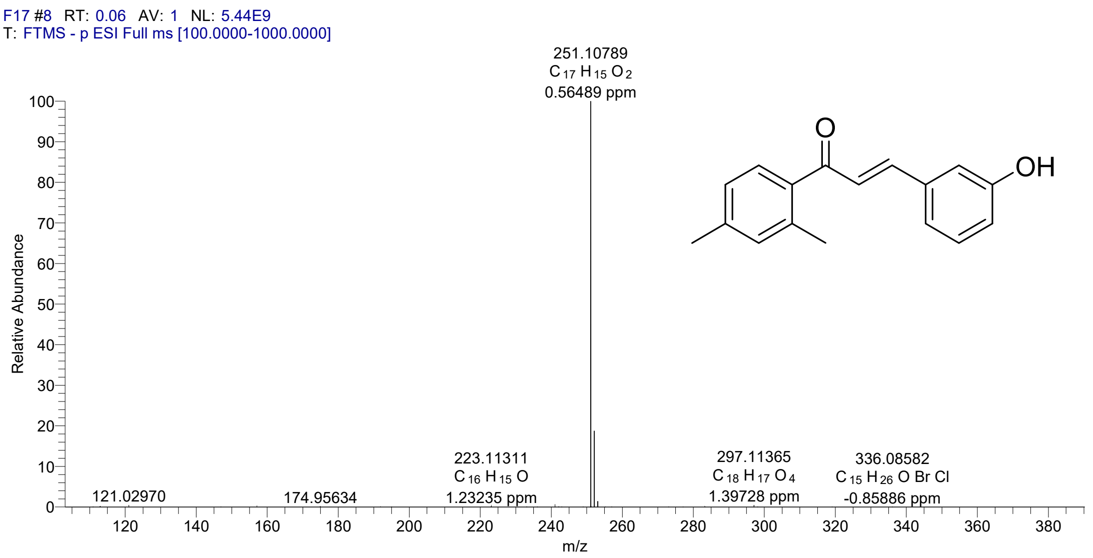

HRMS (ESI) spectrum

The C=O stretching vibration peak at 1652 cm^{-1} and the vibration peaks of O–H groups at 3395 cm^{-1}. The peaks at 302 and 2971 cm^{-1} could be attributed to the stretching vibration of C–H in CH_3. Peaks at 1585, 1555, and 1502 cm^{-1} could be assigned to the stretching vibration of CH_2 in the aromatic nucleus. The peak at 811 cm^{-1} results from the out-of-plane bending vibration of C–H. The peaks at 1378 and 1351 cm^{-1} are ascribed to the stretching vibration of the C-C benzenoid ring. The peaks at 994 and 977 cm^{-1} are assigned to C–H of the methyl group. These observations confirmed that the structure is (E)-1-(2,4-dimethylphenyl)-3-(3-hydroxyphenyl)propyl-2-en-1-one (**F14**).

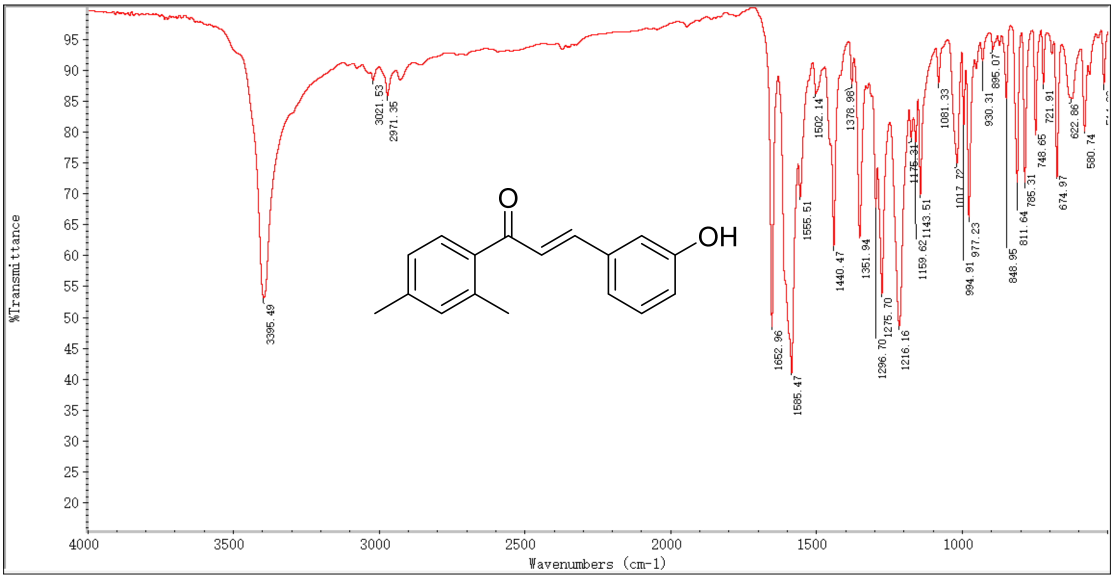

IR spectrum

(E)-3-(2-hydroxyphenyl)-1-(naphthale-1-yl)propyl-2-en-1-one (F15)

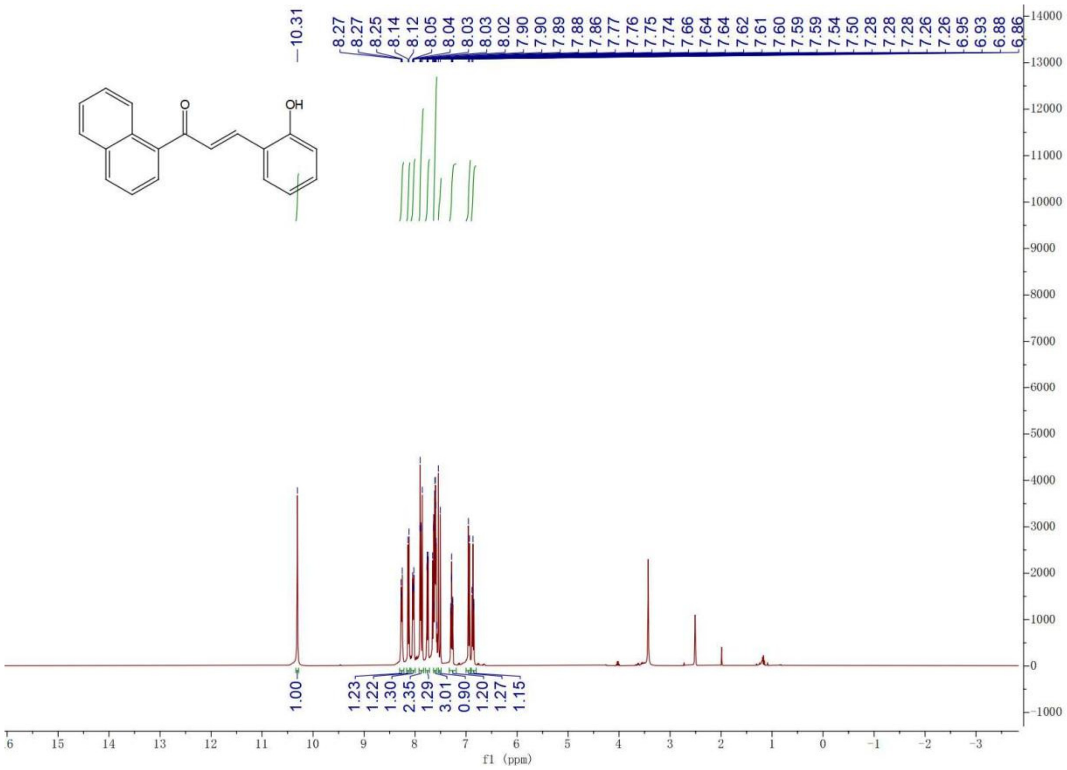

^{1}H NMR spectrum

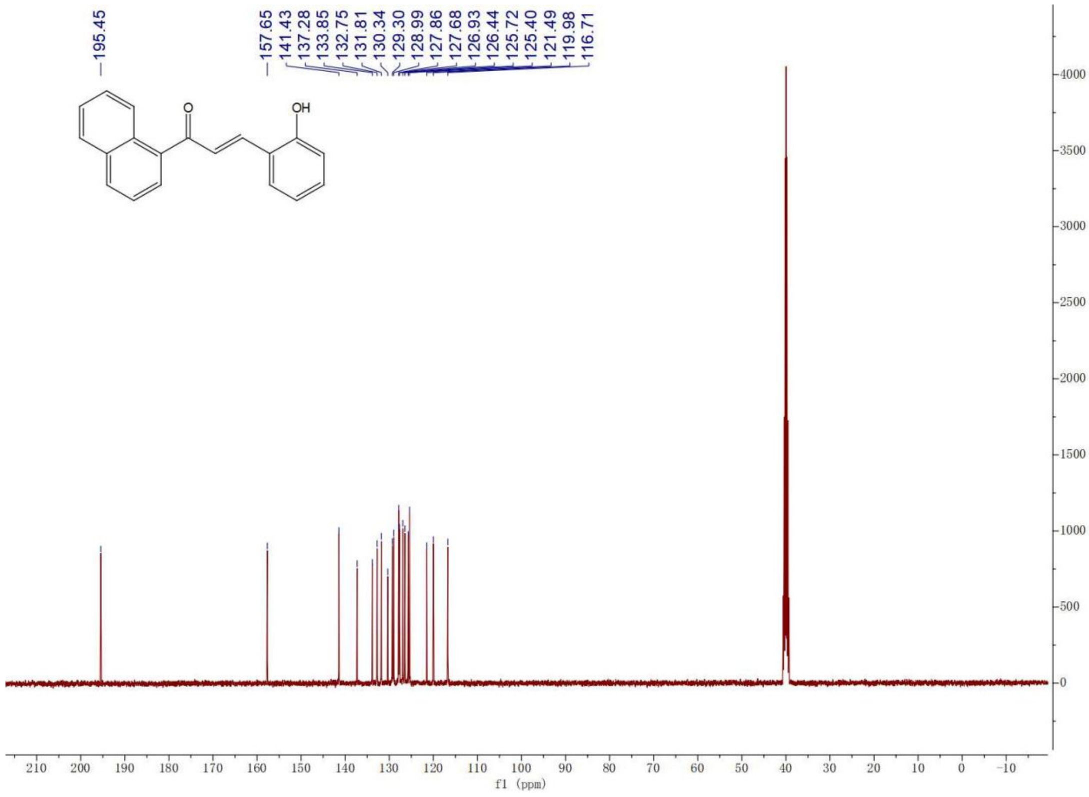

^{13}C NMR spectrum

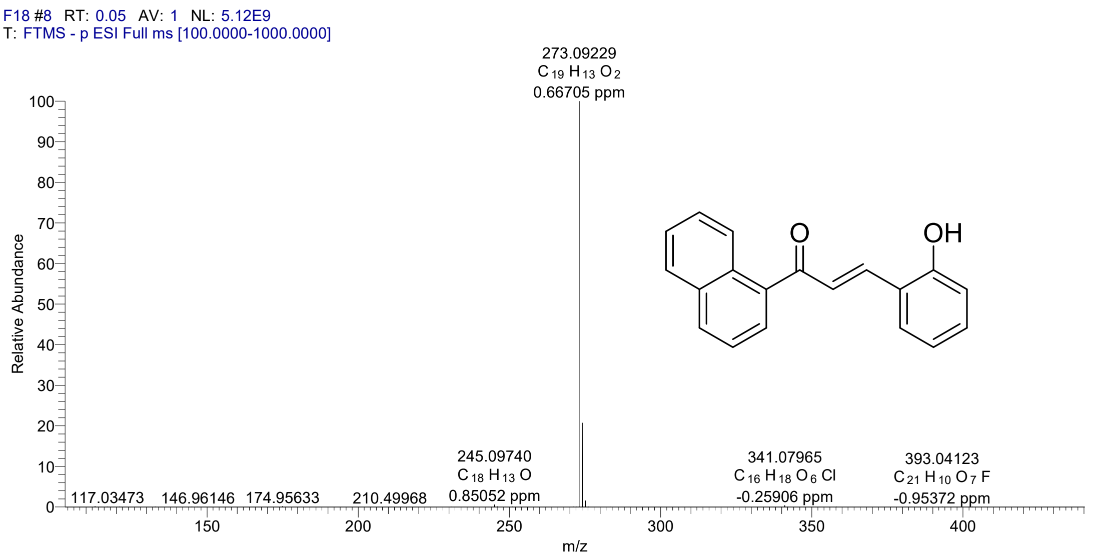

HRMS (ESI) spectrum

The C=O stretching vibration peak at 1649 cm^{-1} and the vibration peaks of O–H groups at 3083 cm^{-1}. Peaks at 1603, 1508, and 1459 cm^{-1} could be assigned to the stretching vibration of CH_2 in the aromatic nucleus. The peak at 779 cm^{-1} results from the out-of-plane bending vibration of C–H. The peaks at 1396 and 1359 cm^{-1} are ascribed to the stretching vibration of the C-C benzenoid ring. These observations confirmed that the structure is €-3-(2-hydroxyphenyl)-1-(naphthale-1-yl)propyl-2-en-1-one (**F15**).

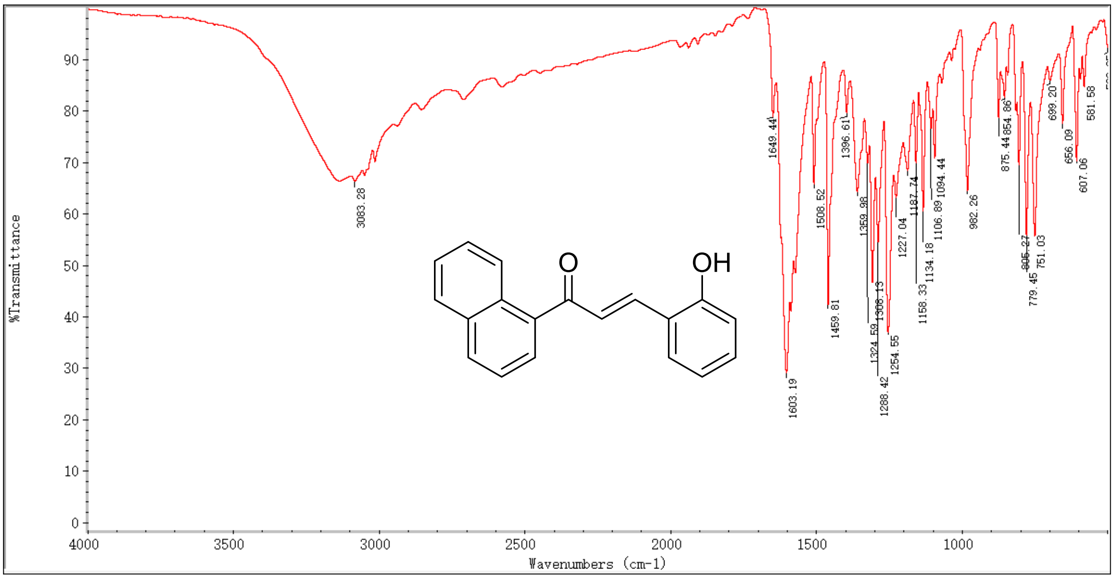

IR spectrum

(E)-3-(2-hydroxyphenyl)-1-(thiophene-2-yl)propion-2-en-1-one(F16)

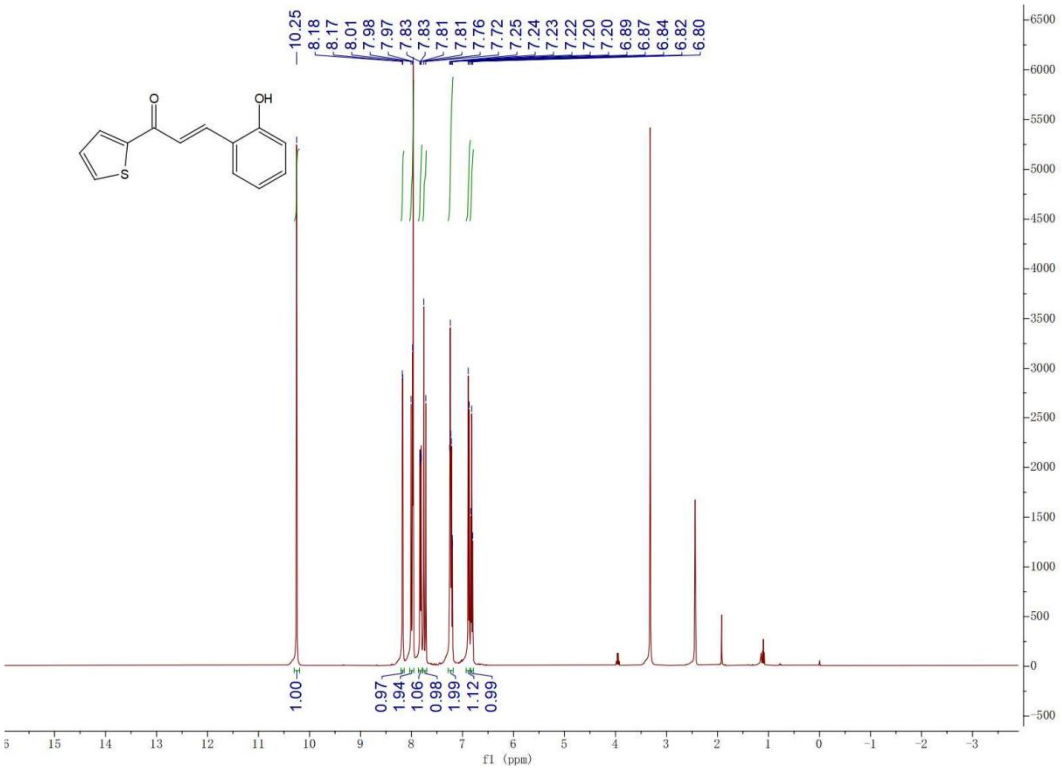

^{1}H NMR spectrum

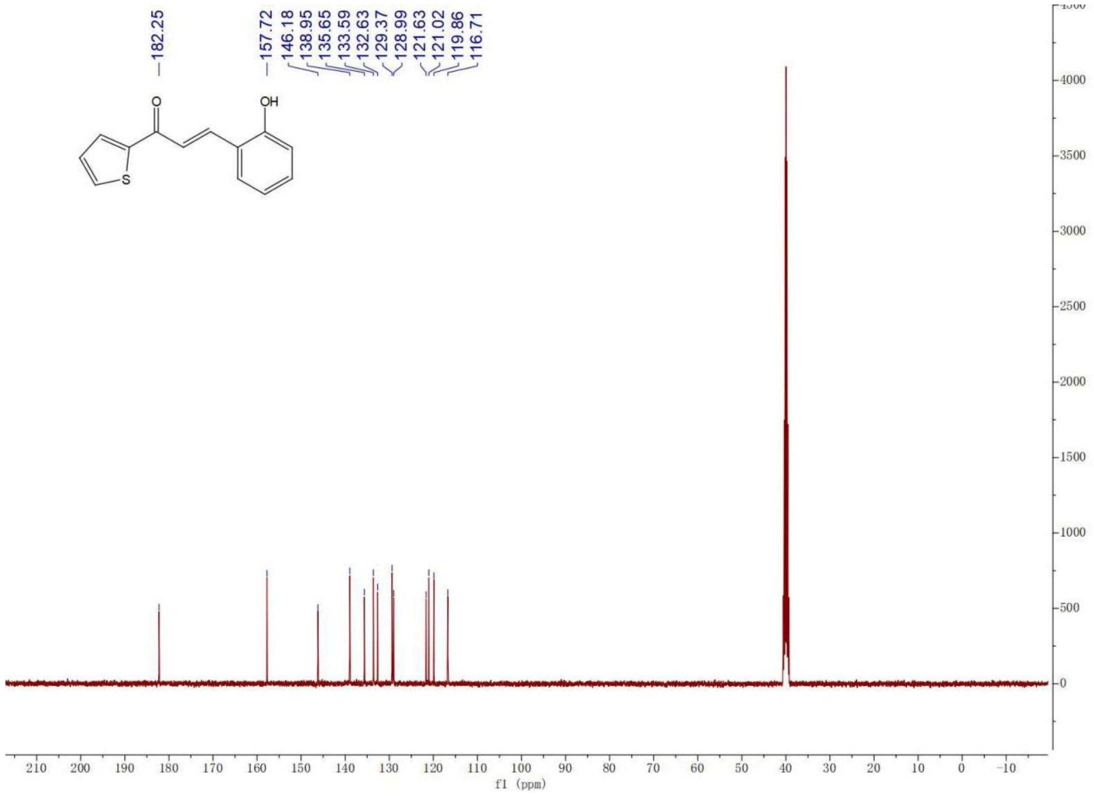

^{13}C NMR spectrum

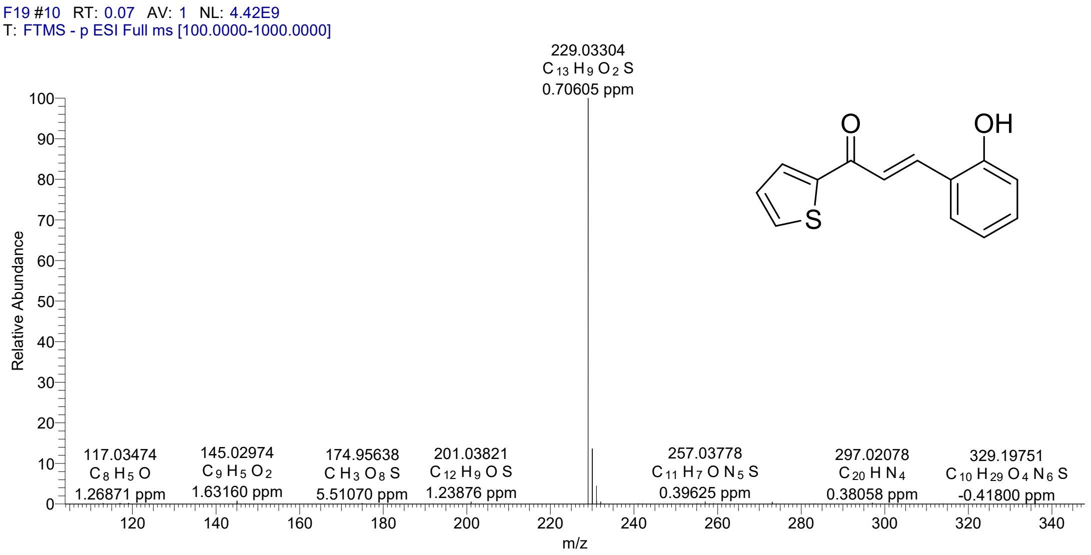

HRMS (ESI) spectrum

The C=O stretching vibration peak at 1636 cm^{-1} and the vibration peaks of O–H groups at 3333 cm^{-1}. The peak at 3096 cm^{-1} is assigned to the stretching vibration of C–H in thiophene. Peaks at 1601, 1572, and 1510 cm^{-1} could be assigned to the stretching vibration of CH_2 in the aromatic nucleus. The peak at 859 cm^{-1} results from the out-of-plane bending vibration of C–H. The peaks at 1322 and 1304 cm^{-1} are ascribed to the stretching vibration of the C-C benzenoid ring. These observations confirmed that the structure is (E)-3-(2-hydroxyphenyl)-1-(thiophene-2-yl)propion-2-en-1-one (**F16**).

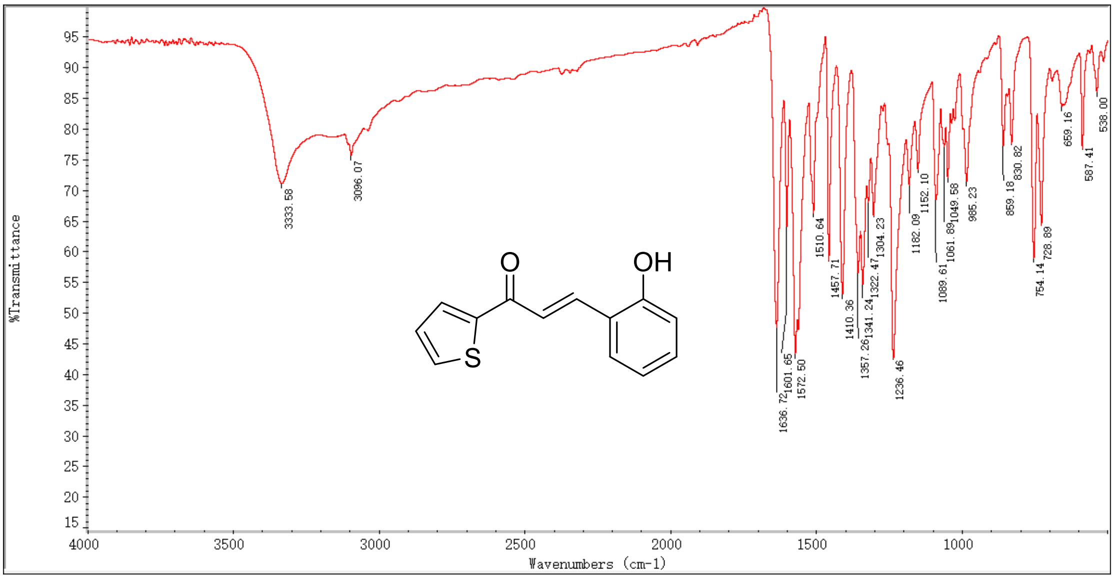

IR spectrum
